# Versatile Stimuli-Responsive Controlled Release of Pinanediol-Caged Boronic Esters for Spatiotemporal and Nitroreductase-Selective Glucose Bioimaging

**DOI:** 10.1021/acssensors.4c02811

**Published:** 2025-01-03

**Authors:** Chih-Yao Kao, Ying-Wei Chen, Yu-Cheng Liu, Jen-Hsuan Wei, Tsung-Shing Andrew Wang

**Affiliations:** † Department of Chemistry and Center for Emerging Material and Advanced Devices, National Taiwan University, Taipei 106319, Taiwan (R.O.C.); ‡ Institute of Molecular Biology, Academia Sinica, Nankang, Taipei 115201, Taiwan (R.O.C.)

**Keywords:** boronic acids, pinanediol, stimuli-responsive, self-immolative, glucose sensing

## Abstract

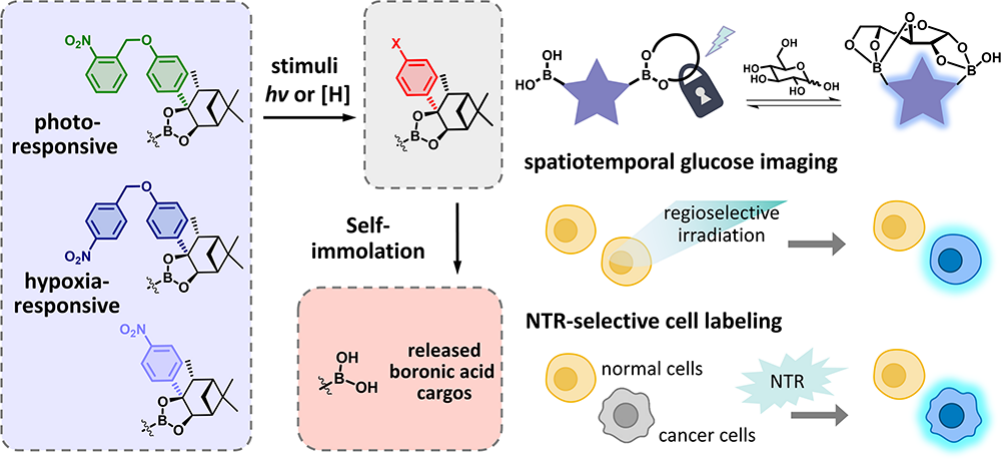

Boronic acids have been widely applied in various biological fields, particularly achieving significant practical progress in boronic acid–based glucose sensing. However, boronic acids exhibit nonspecific binding to other nucleophiles, and the inherent lability of boronic esters in biological systems limits their further applications. Herein, we developed a stimuli-responsive controllable caging strategy to achieve photoresponsive spatiotemporally and nitroreductase-responsive cancer cell-selective glucose sensing. We introduced *o*-/*p*-nitroaryl-containing self-immolative linkers onto δ-pinanediol derivatives, effectively caging boronic acids and blocking glucose recognition. Upon triggering by specific stimuli, the caged boronic esters decompose, releasing boronic acids and thereby restoring glucose recognition of the diboronic acid–based sensor. The proof of concept was confirmed through intracellular glucose bioimaging in living cells. Upon regional UV irradiation, we could monitor intracellular glucose with excellent spatiotemporal selectivity. Furthermore, we used the cancer biomarker nitroreductases as the internal stimuli and utilized the caged glucose sensor to selectively label hypoxic cancer cells in a cocultured living cell sample. We believe that our stimuli-responsive caging strategies will hold promising potential for the controlled release of other boronic acids in various biological contexts.

Boronic acids have garnered significant attention in chemical biology due to their promising applications in biological systems.^1−10^ Their selective reactivity with diol- and triol-containing compounds makes boronic acids highly attractive for the design of biological sensors targeting specific diol species.^11−15^ In particular, their reversible covalent binding mechanism with glucose enables boronic acids to be used in continuous glucose monitoring applications and has become a popular topic in recent decades.^16^ To achieve selectivity to glucose, the structure of diboronic acid (DBA) has been explored to differentiate glucose from other monosaccharides.^17^ The design of DBA derivatives matches both the distance between the two potential binding sites of glucose and the orientation of the hydroxyl groups, thereby providing strong binding affinity to glucose and inducing a rearrangement into the thermodynamically more stable sensor-glucopyranose complex under aqueous conditions, enabling selective glucose recognition.^18^ Subsequent research further optimized glucose selectivity and biocompatibility, facilitating boronic acid–based glucose sensing in living cells and even in living animals.^19^

Nevertheless, boronic acid–based glucose sensing remains largely unexplored and faces significant challenges. For instance, cancer cells are reported to have an increased glucose demand under hypoxic conditions, and several studies have suggested that glucose metabolism in cancerous cells is altered.^20,21^ Therefore, it is important for glucose sensors to distinguish between different cell types or cells in varying physiological states to better understand cellular heterogeneity. On the other hand, the lack of spatiotemporal selectivity poses challenges as well.^22,23^ Due to the inherent lability of boronic esters in biological systems,^24^ current probes may struggle to meet the requirements for both high temporal and spatial resolution simultaneously, making precise spatiotemporal measurements in complex biological systems difficult.^25,26^

To address these challenges, we introduced light and nitroreductases (NTR) as external and internal stimuli, respectively, to achieve controllable activity manipulation. Light has been regarded as an attractive trigger to achieve that due to its ability to precisely induce reactions within specific spatial and temporal ranges.^27^ Meanwhile, NTR, which can be overexpressed in hypoxic cancer cells,^28−31^ serve as an internal stimulus ideal for selectively distinguishing between normal and tumor cells.^32,33^ On the other hand, reactive oxygen species (ROS) is also recognized as an important potential trigger.^34^ Although ROS-responsive strategies for boronic acids and esters are already well-established and widely applied, the reaction with ROS ultimately converts the boronic group into a hydroxyl group, resulting in the loss of glucose recognition capability. Therefore, both light and NTR were incorporated into our strategy to achieve spatiotemporal and cellular selectivity. We aim to improve the spatiotemporal selectivity of boronic acid–based glucose sensing through regioselective irradiation and differentiate normal and tumor cells under complex conditions using NTR overexpressed in cancer cells (Figure 1a).

**Figure 1:**
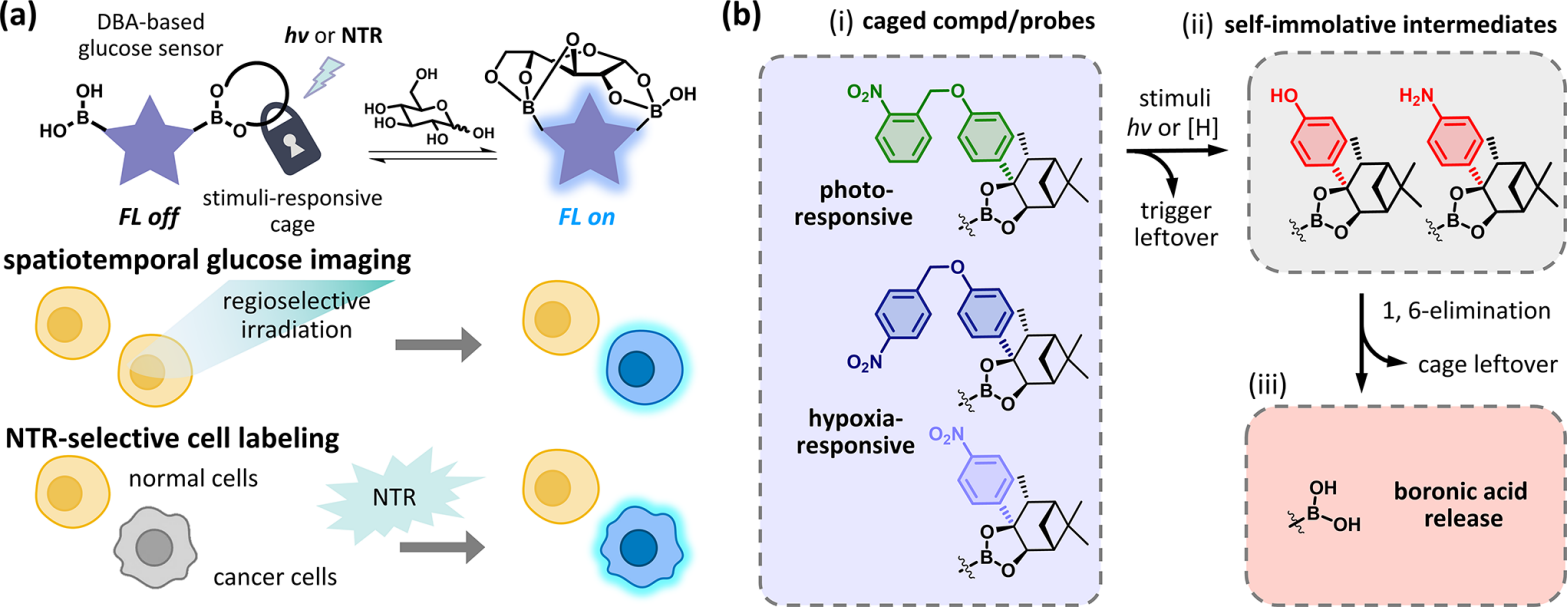
(a) Our stimuli-responsive controllable strategy for DBA-based glucose sensors to achieve spatiotemporal and NTR-selective glucose bioimaging. The five-pointed star represents a DBA-based glucose sensor. The lock symbol represents a stimuli-responsive pinanediol cage. (b) Schematic of the stimuli-responsive release of the pinanediol-caged boronic esters: (i) structures of boronic esters caged by photo-/NTR-responsive pinanediol cages; (ii) self-immolative phenyl/anilino moieties revealed upon exposure to specific stimuli; (iii) release of boronic acids via 1,6-elimination.

Herein, we selected pinanediol as the cage scaffold to design cages to block glucose recognition of the DBA-based glucose sensor, because it is known to form kinetically and thermodynamically stable boronic esters.^24,35^ Furthermore, *o*-/*p*-nitroaryl groups were introduced as stimuli-responsive self-immolative linkers. Upon irradiation with UV light^36,37^ or reduced by NTR,^38,39^ the self-immolative phenyl/anilino moieties are exposed, leading the caged boronic ester to convert to a less stable intermediate that is prone to elimination, thereby releasing the boronic acid cargo and restoring glucose recognition (Figure 1b).

## Results and Discussion

### Using δ-Pinanediol as the Cage Scaffold

According to previous research, steric hindrance around the boron atom might be the most important factor in the stability of boronic esters.^35^ Pinanediol is considered a strong candidate for the main scaffold of the cage because of its bulky structure.^24^ Therefore, we prepared α-pinanediol and its regioisomer, δ-pinanediol, as cage scaffolds to investigate the stability of boronic esters (Scheme S1).

We first evaluated the hydrolytic stability of **tPin-EtPh (2)**, the boronic esters derived from α-pinanediol, and **k/tPin-EtPh (7/2)**, the boronic esters derived from a synthesized pinanediol mixture of δ-pinanediol and α-pinanediol (the molar ratio of **7** and **2** = 3:1). Hydrolysis assays were carried out in a PBS/DMSO mixture. Both boronic esters demonstrated incomplete hydrolysis (Figure S1a,b). As observed by high-performance liquid chromatography (HPLC), **tPin-EtPh (2)** remained at 20%, and **k/tPin-EtPh (7/2)**, the mixture of boronic esters remained 40% boronic esters (Figure S1c). Similar results were observed in the dynamic assembly assays (Figure S1d), in which the formation and hydrolysis of the boronic esters reached equilibrium within 4 h. These results indicate that both α-pinanediol and δ-pinanediol can lead to incomplete hydrolysis and the dynamic formation of boronic esters. Moreover, the configuration of δ-pinanediol seems to be more conducive to the dynamic condensation of boronic esters. Furthermore, the property of dynamic assembly between pinanediols and boronic acids shows potential for developing a strategy that enables late-stage installation^40^ of a diol cage to avoid the loss of purification of boronic esters and provides a convenient method to evaluate various diol cages for boronic acid cargos.

### Design and Characterization of the Substituted Pinanediol Cages

**Figure 2:**
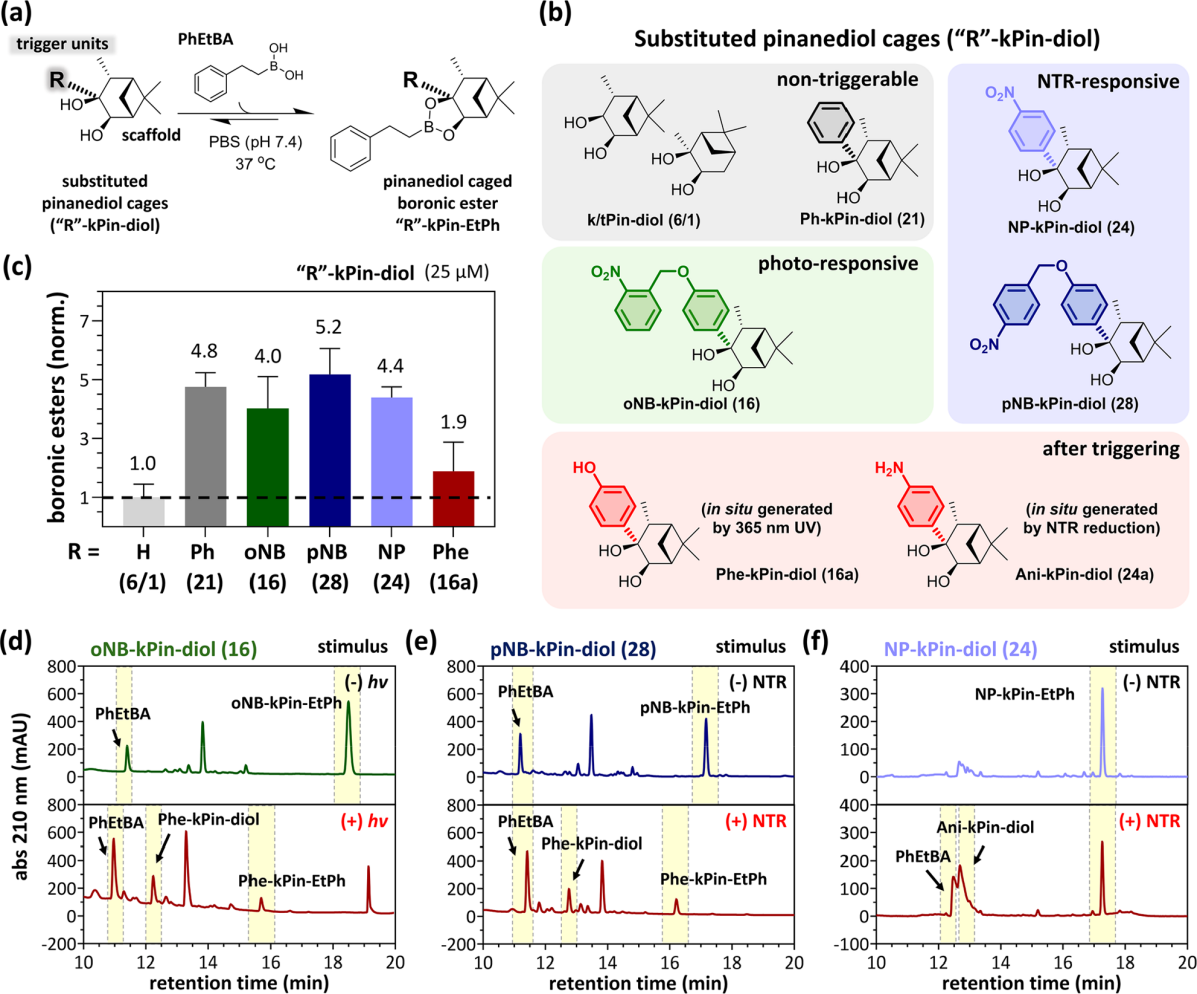
Validation studies of the dynamic assembly and controlled release of pinanediol cages. (a) Schematic of the assembly reaction between the pinanediol cages and **PhEtBA**. (b) Chemical structures of the substituted pinanediol cages (**“R”-kPin-diol**). (c) Quantification results of the assembly assays of the **k/tPin-diol (6/1)** (molar ratio = 3:1), **Ph-kPin-diol (21)**, **oNB-kPin-diol (16)**, **Phe-kPin-diol (16a)**, **pNB-kPin-diol (28)**, and **NP-kPin-diol (24)** with **PhEtBA** (25 μM each) in PBS with 2% DMSO at 37 °C for 4 h by HPLC. HPLC of the assemblies of (d) **16** (25 μM, (−) *hv*) and **16a** (25 μM, (+) *hv*), (e) **28** (25 μM, (±) NTR), and (f) **24** (25 μM, (±) NTR) with **PhEtBA** (25 μM) in PBS with 2% DMSO at 37 °C for 4 h. (+) NTR conditions: NTR (25 μM) and NADH (250 μM).

To achieve the controllable release of boronic acids, we introduced substituents as trigger units onto the pinanediol scaffolds. Several δ-pinanediol cages were prepared (Schemes S2–S5) to investigate the effects of substitution on the hydrolytic stability of boronic esters. Dynamic assembly assays were carried out with these pinanediol cages with different substituents and the boronic acid **PhEtBA** (Figure 2a,b). We used the pinanediol mixture **k/tPin-diol (6/1)** (the molar ratio of **6** and **1** = 3:1) to represent the pinanediol cage without a bulky substituent and compared the boronic ester formation with that of **Ph-kPin-diol (21)** containing one aryl group. As a result, **21** formed a 4.8-fold greater amount of boronic ester than did **k/tPin-diol (6/1)** within 4 h, indicating that sufficient steric hindrance is beneficial to the hydrolytic stability of boronic esters (Figures 2c and S2).

We hypothesized that self-immolative chemistry could be utilized to weaken the bonding of boronic esters to achieve a controllable release of boronic acids. Three kinds of trigger units were introduced onto the pinanediol scaffolds (Figure 2b). The trigger unit on **oNB-kPin-diol (16)** comprises a phenoxy linker and an *o*-nitrobenzyl moiety, which is photoresponsive and undergoes photolysis upon 365 nm irradiation; this prompts the exposure of the self-immolative phenol group (Scheme S6a).^36,37^ In the case of **pNB-kPin-diol (28)**, the trigger unit similarly contains a phenoxy linker and carries an NTR-responsive *p*-nitrobenzyl moiety. Moreover, in **NP-kPin-diol (24)**, a *p*-nitrophenyl moiety was directly introduced into the pinanediol scaffold. Both pinanediol cages were expected to respond to NTR. Upon reduction, they were shown to undergo 1,6-elimination, leading to the release of their cargo (Scheme S6b,c).^38,39^ As observed by HPLC, **16**, **28**, and **24** demonstrated similar results as **21**, with 4.0-, 5.2-, and 4.4-fold boronic ester formation as the mixture of **k/tPin-diol (6/1)** within 4 h, respectively. This enhanced formation is attributed to the steric hindrance provided by the bulky trigger units (Figure 2c). Notably, **24** generated boronic esters in similar yields as **21**. Although the incorporation of electron-withdrawing groups was considered to affect the hydrolytic stability of boronic esters,^41^ the results indicated that the electronic properties of the substituents might not be crucial factors in our design. On the other hand, the triggerable α-pinanediol derivative **oNB-tPin-diol (13)** was synthesized and subjected to an assembly assay. However, no corresponding boronic esters were observed after 4 h of incubation (Figure S3). The crowdedness around the diol moieties ultimately hindered the condensation of boronic acids.

The responsiveness of **oNB-kPin-diol (16)** toward UV light and the reactivities of **pNB-kPin-diol (28)** and **NP-kPin-diol (24)** toward nitroreductase NfsB were confirmed via LC–MS.^42,43^ Upon irradiation, **16** converted to pinanediol with a phenol group, identified as **Phe-kPin-diol (16a)** (Figure S4). The photouncaged pinanediol, **16a**, was subjected to a dynamic assembly assay. Consequently, **16a** formed only 1.9-fold more boronic esters than did the mixture of **k/tPin-diol (6/1)** after 4 h of incubation, which was much lower than that of the untriggered pinanediol cage, **16** (4.0-fold) (Figure 2c,d). On the other hand, in the presence of NTR and the coenzyme NADH, similar results were observed. **pNB-kPin-diol (28)** was fully reduced, while **NP-kPin-diol (24)** was partially reduced, yielding **Phe-kPin-diol (16a)** and **Ani-kPin-diol (24a)**, respectively (Figures 2e,f, S5, and S6). Boronic esters formed by reduced **28** were observed via HPLC and LC–MS. Although the boronic ester formed by reduced **24** was not detected, the generation of **24a** was accompanied by increased free boronic acid, as observed via HPLC (Figure 2f). In summary, in the dynamic assembly assay with a triggered pinanediol cage, the formation of boronic esters by **16a** (1.9-fold) was lower than that of **21** (4.8-fold), despite both pinanediol cages containing an aryl group (Figure 2c). These results support our hypothesis that the phenol or aniline moiety on a pinanediol cage can act as a self-immolative linker, destabilizing the boronic ester and leading to the release of the boronic acid.

### Mechanistic Study of the Release Strategies of Pinanediol-Caged Boronic Esters

**Figure 3:**
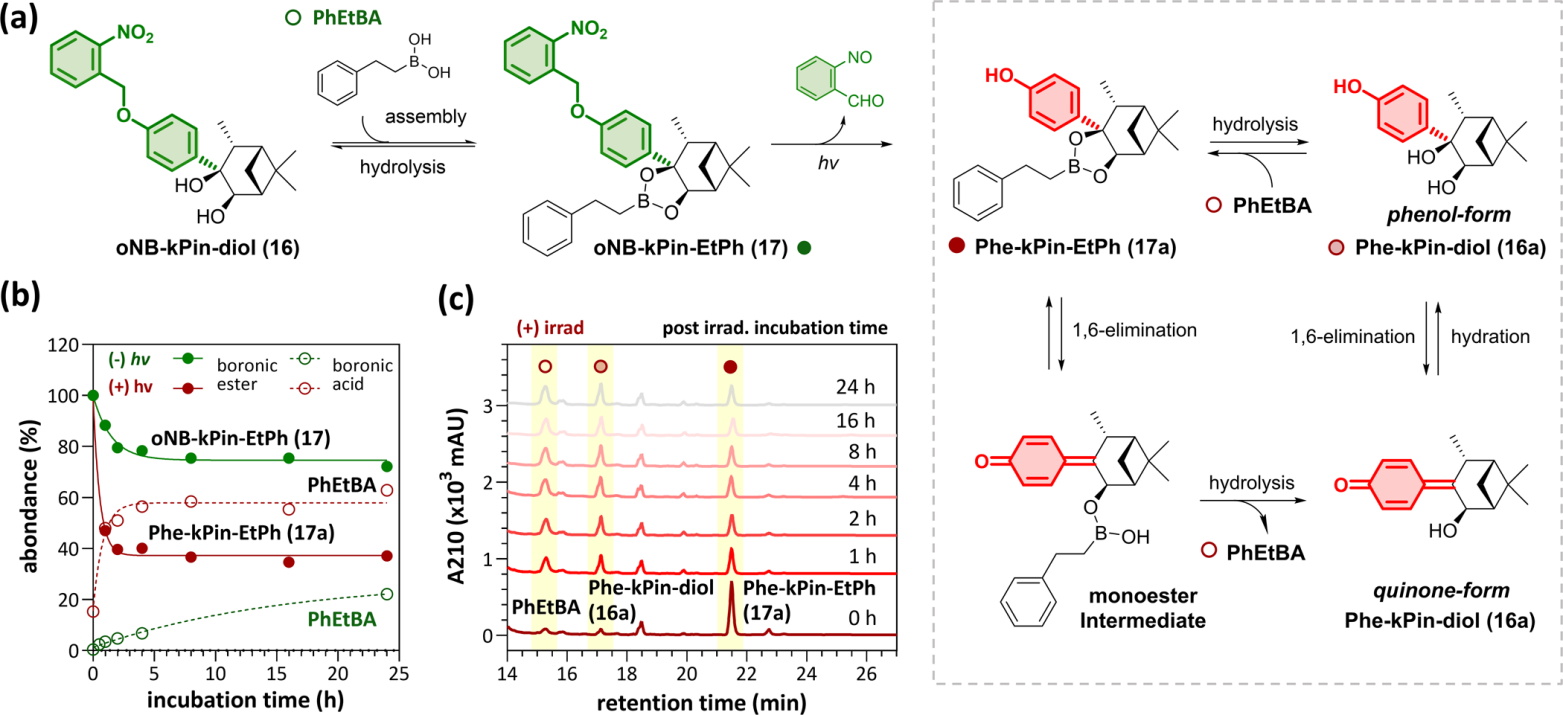
Mechanistic study of the release strategy of **oNB-kPin-EtPh (17)**. (a) Assembly/hydrolysis and photolysis of **17**. Proposed hydrolysis mechanism after photolysis (dashed gray box). (b) Quantification results of the time course of the hydrolysis assays of **17** (250 μM) (±) under 365 nm irradiation in PBS with 50% DMSO via HPLC. (c) HPLC of the time course of the subsequent hydrolysis of **Phe-kPin-EtPh (17a)** (250 μM, +365 nm irradiation) in PBS with 50% DMSO.

To further understand the release mechanism of a caged boronic acid, we prepared **oNB-kPin-EtPh (17)** by condensing **oNB-kPin-diol (16)** and **PhEtBA** and then evaluated the hydrolytic stability with or without UV irradiation via LC–MS (Figure 3a). The hydrolysis reaction reached equilibrium within 4 h, and 80% of the boronic ester remained after 24 h of incubation (Figure 3b). Upon irradiation, **17** was converted to the expected photouncaged phenol intermediate, **Phe-kPin-EtPh (17a)**. During postirradiation incubation (0–24 h), subsequent hydrolysis of **17a** was observed, affording **Phe-kPin-diol (16a)** and **PhEtBA** (Figure 3c), and only 37% of the boronic ester remained after 24 h. Similarly, the presence of the free phenol moiety caused a decrease in the hydrolytic stability of the caged boronic ester. Although a phenol group provides considerable steric hindrance, we believe that 1,6-elimination predominantly decreases the stability of the boronic ester. Thus, through 1,6-elimination, **17a** could form a less sterically hindered monoester intermediate, which is more prone to hydrolysis. Under LC–MS monitoring, purified **16a** was observed as two close peaks. Both peaks displayed MS signals corresponding to their phenol and quinone forms (Figure S7), generated from **16a** through 1,6-elimination. We believe that the quinone form of **16a** interferes with the formation of boronic esters during dynamic assembly. Although the detailed mechanism is worthy of further studies, our self-immolation strategy has been shown to effectively release boronic acid, enabling further applications.

### Pinanediol-Caged Boronic Esters Show High Tolerance to Environmental pH

The environmental pH is known to be one of the most important factors affecting the formation of boronic esters.^44−46^ In particular, the acid instability of boronic esters limits their application under acidic conditions. Therefore, we evaluated the ability of our stimuli-responsive pinanediol cages, **16**, **28**, and **24**, and the photouncaged pinanediol, **16a**, with boronic acid **PhEtBA** over a wide range of pH values, to be dynamically assembled and monitored by HPLC (Figure S8). **16a** was selected as the representative triggered pinanediol cage because its high photolysis efficiency facilitates rapid preparation and ensures reliable comparisons. In contrast, the low conversion of **24**–**24a** by NTR made the assembly assays for **24a** difficult and inconclusive (Figure 2f).

For all the cages, a pH dependency was observed, in which the formation of boronic esters reached their maximum at approximately pH 7 and decreased under acidic or basic conditions. Notably, a considerable portion of boronic esters remained even at pH 3 or pH 11. We believe that the limited pH dependency of boronic ester formation is due to the large steric hindrance of the pinanediol cages, which dominates the stability of the boronic esters. Therefore, our cages showed high tolerance toward hydrolysis over a wide range of pH. We believe that the 1,6-elimination occurring after triggering dominates the decrease in the stability of the boronic esters, making our pinanediol cages applicable for the controlled release of boronic acids and enabling control in glucose detection.

### Employment of Our Controllable Strategies for Glucose-Sensitive DBA-Based Sensors

**Figure 4:**
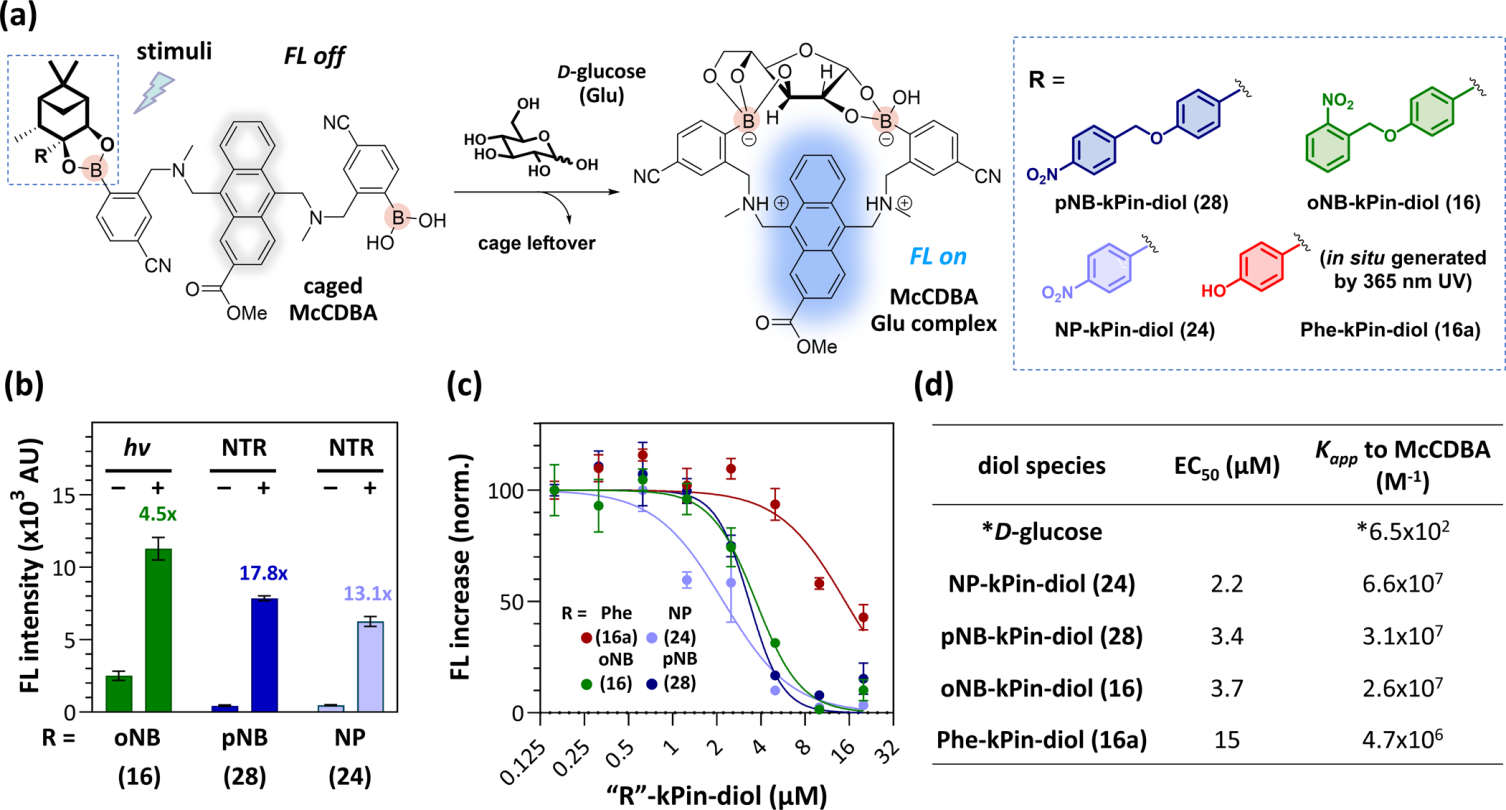
Demonstration of stimuli-responsive pinanediol cages for glucose sensor **McCDBA**. (a) Schematic of the stimuli-responsive release strategy for **McCDBA** and chemical structures of the substituted pinanediol cages. (b) Quantification of the fluorescence intensity at 450 nm of **McCDBA** (5 μM) with **oNB-kPin-diol (16)**, **Phe-kPin-diol (16a)**, **pNB-kPin-diol (28)**, or **NP-kPin-diol (24)** (12.5 μM each) in PBS with 1% DMSO at 37 °C for 2 h. NADH (250 μM) and NTR (25 μM) were added as stimuli for NTR-responsive pinanediol cages **28** and **24**. (c) Glucose-competitive titrations of **McCDBA** (5 μM) with **16**, **16a**, **28**, or **24** (0–25 μM) in DMEM with 1% DMSO. (d) EC_50_ (μM) and apparent binding constants (M^–1^) of **16**, **16a**, **28**, or **24** to **McCDBA** determined from the titrations in (c). * The binding constant (M^–1^) of glucose to **McCDBA** was determined via a Benesi–Hildebrand plot.

Our pinanediol cages have been confirmed to be applicable under physiological conditions. Subsequently, we aimed to integrate pinanediol cages with a DBA-based glucose sensor to achieve glucose detection with precise control and cell specificity (Figure 4a). The DBA-based glucose sensor **McCDBA**, which incorporates anthracene as the fluorescent core and two boronic acid moieties, shows improved water solubility and fluorescence sensitivity.^19^ In the absence of glucose, photoinduced electron transfer (PET) between the nitrogen atom and anthracene quenches fluorescence, but glucose binding enhances the B–N interaction, inhibiting PET and restoring fluorescence.^47^

**McCDBA** was prepared,^19^ and its glucose-dependent fluorogenic property was confirmed by emission spectroscopy (Figure S9). As shown in Figure 4b, when **McCDBA** was incubated with the pinanediol cages, **oNB-kPin-diol (16)**, **pNB-kPin-diol (28)**, or **NP-kPin-diol (24)**, its fluorescence intensity remained low. In contrast, when incubated with the photouncaged pinanediol, **Phe-kPin-diol (16a)**, the fluorescence of **McCDBA** was significantly enhanced. Similarly, in the case of **24** and **28**, the addition of NTR after 2 h of incubation also resulted in stronger fluorescence. Additionally, the formation of the corresponding boronic esters was confirmed by LC–MS (Figure S10). These results are consistent with our previous observations (Figure 2c) and support the idea that pinanediol cages can be applied via dynamic assembly to caged boronic esters. The pH dependency of **McCDBA** with the four pinanediol cages was also examined in the presence of 0.1 M glucose from pH 3 to 11. Similarly, **16**, **28**, or **24** strongly suppressed the fluorescence of **McCDBA** (Figure S11). In contrast, the fluorescence remained in the presence of compound **16a**. Taken together, our observations not only highlight the feasibility of implementing our strategies for the controlled release of boronic acids under physiological conditions but also suggest their potential for broader applications.

### Binding Affinity of the Pinanediol Cages for McCDBA

At low concentrations, glucose prefers to form a 1:1 complex with **McCDBA**,^17,48^ and our pinanediol cage can compete with glucose to form a **McCDBA** boronic ester, which can be simplified as a 1:1 competition model to better understand the applicability of our stimuli-responsive glucose detection (Figure S12). Moreover, the binding affinity between **McCDBA** and our four pinanediol cages was estimated via a glucose-competitive titration assay in Dulbecco’s Modified Eagle Medium (DMEM) (Figure 4c,d). First, the binding constant (*K*_a_) of **McCDBA** to glucose was calculated as 6.5 × 10^2^ M^–1^ via a B–H plot (Figure S13). Afterward, the apparent binding constant (*K*_app_) of our four pinanediol cages to **McCDBA** can be determined in the presence of 0.1 M glucose (see Section 4 in the Supporting Information for details). The *K*_app_ of **16** to **McCDBA** (2.6 × 10^7^ M^–1^) is similar to those of **28** (3.1 × 10^7^ M^–1^) and **24** (6.6 × 10^7^ M^–1^) but higher than the *K*_app_ of **16a** (4.6 × 10^6^ M^–1^). In summary, the *K*_app_ of **16a** is apparently lower than those of the other three pinanediol cages, which suggested that our stimuli-responsive cages decreased their binding affinity after triggering, enabling us to tune the binding affinity via irradiation or reduction by NTR. Notably, although the photouncaged pinanediol **16a** exhibited lower binding affinity toward **McCDBA**, the binding constant (4.6 × 10^6^ M^–1^) is still much greater than that of glucose. Owing to the abundance but weaker binding affinity of glucose, **McCDBA** with our pinanediol cages can exhibit good sensitivity at a lower concentration upon exposure to stimuli.

### Our Photoresponsive Strategy Provides Glucose Detection with Spatiotemporal Selectivity

**Figure 5:**
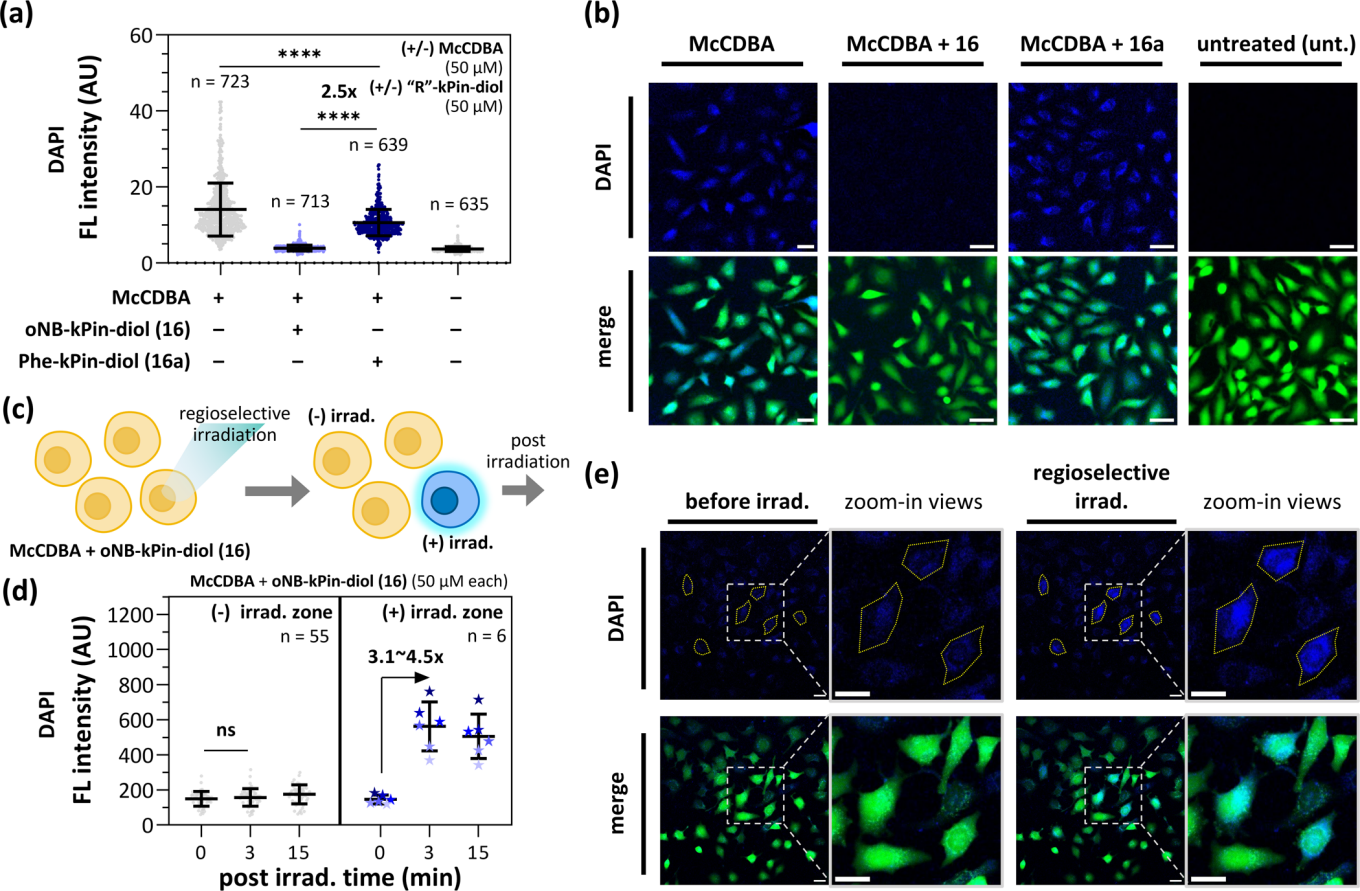
Demonstration of controllable cellular glucose detection by **oNB-kPin-diol (16)** with **McCDBA**. (a) Scatter plots, each dot represents an individual cell, and (b) fluorescence microscopy images of calcein AM-stained HeLa cells treated with **McCDBA** alone (50 μM), **McCDBA**+**16** (50 μM each), **McCDBA**+**16a** (50 μM each), or DMSO (unt.) in DMEM with 1% DMSO at 37 °C for 30 min. (c) Controllable cellular glucose detection by regioselective irradiation. (d) Scatter plots of HeLa cells before and after regioselective irradiation with a 405 nm laser. Calcein AM-stained HeLa cells were treated with **McCDBA**+**16** (50 μM each) in DMEM with 1% DMSO at 37 °C for 30 min before irradiation. After regioselective irradiation, the cells in the (−) and (+) zones were further monitored for 15 min. (e) Fluorescence microscopy images of the cells in (d). Yellow enclosed zones (six cells) indicate the regioselective irradiation areas, and the white boxes indicate the zoomed-in views. DAPI channel: fluorescence of **McCDBA**; merge channel: fluorescence of **McCDBA** and calcein AM. Scale bars: 50 μm.

To evaluate the ability of our photoresponsive strategy to detect cellular glucose, calcein AM-stained HeLa cells were treated with **McCDBA** alone, **McCDBA**+**16**, or **McCDBA**+**16a** and monitored via fluorescence microscopy. Here, **McCDBA** was first added to the cells, followed by the addition of pinanediol cages to facilitate dynamic assembly to form boronic esters with **McCDBA**. Within 30 min of incubation with **McCDBA** alone, increased fluorescence was observed in the cytoplasm, indicating that HeLa cells can efficiently take up **McCDBA** and that glucose is predominantly located in the cytoplasm, which is consistent with previous reports (Figure S14).^49^ In contrast, the fluorescence was dramatically suppressed in cells treated with **16**. On the other hand, apparent fluorescence remained in cells treated with **16a** (∼2.5-fold greater than that of **McCDBA**+**16**) (Figure 5a,b). Notably, no significant morphological changes or cell death were observed after treatment, indicating the high biocompatibility of our cages used for glucose detection. The Alamar Blue assay also indicated that **McCDBA** or the two caged **McCDBA** complexes showed no significant cytotoxicity at a working concentration of 50 μM (Figure S15a). Afterward, we demonstrated the direct photoactivation of cellular-caged **McCDBA** by confocal microscopy (Figure 5c). Similarly, the fluorescence of **McCDBA** was dramatically suppressed by the use of **16**. Subsequently, six cells were regioselectively photouncaged by a 405 nm laser. A significant increase in **McCDBA** fluorescence was observed immediately after laser irradiation, without notable morphological changes in these six cells (Figure 5d,e). Moreover, there was no further significant change in fluorescence observed within 15 min, and other unirradiated cells were unaffected, indicating the potential for continued glucose detection at the single-cell level. Notably, the average fluorescence intensity in the irradiated cells was even stronger than that in the cells treated with **McCDBA** alone (Figure S16), suggesting that the boronic ester, formed by dynamic assembly, has better cellular uptake due to increased lipophilicity. In summary, our photoresponsive release strategy can be effectively applied in living cells. Furthermore, the combination of photochemistry and boronic acid chemistry provides an attractive option for conducting versatile biological research involving boronic acids.

### Our Nitroreductase-Responsive Strategy Assists Glucose Sensors in the Selective Labeling of Cancer Cells

**Figure 6:**
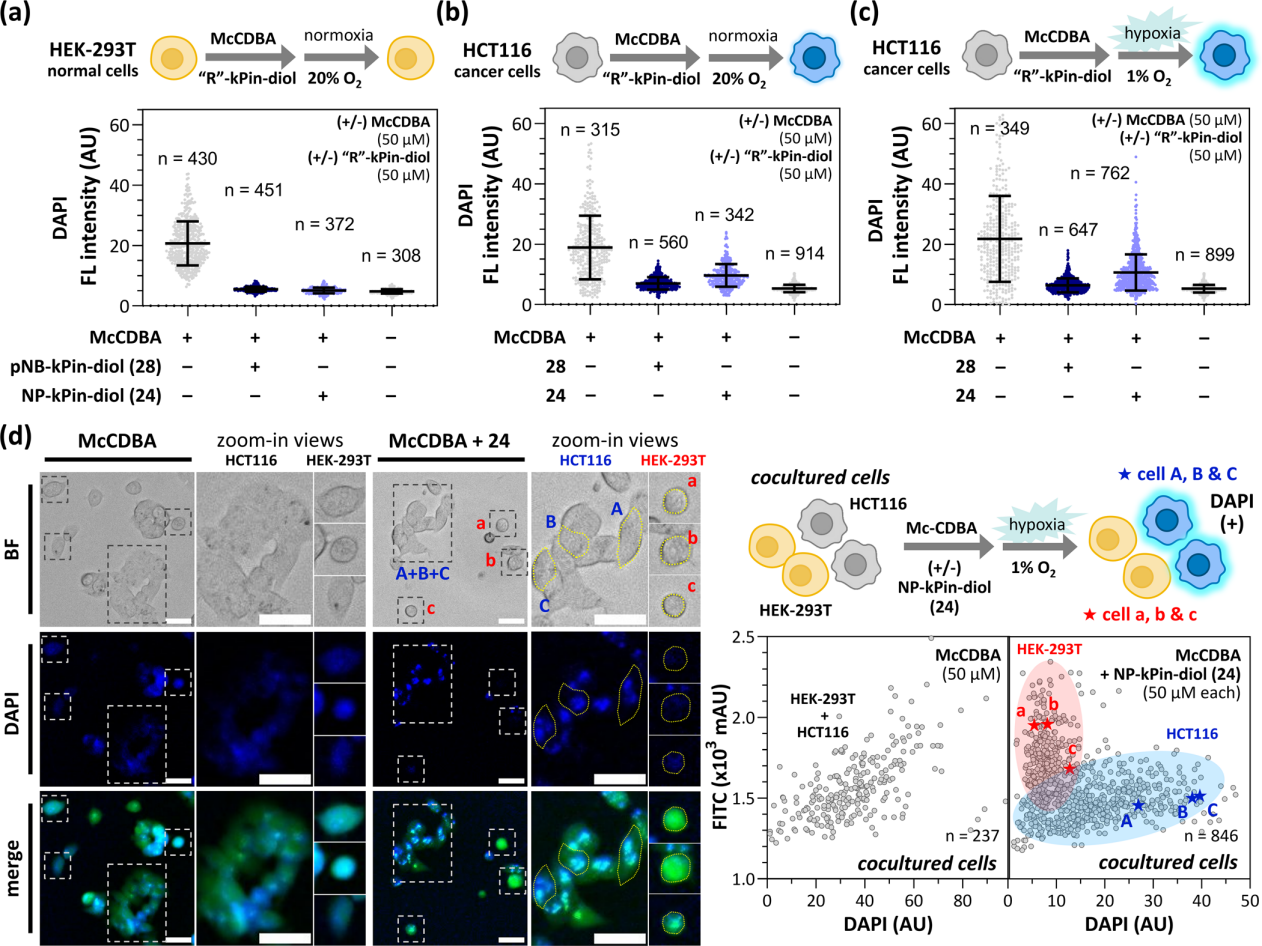
Demonstration of cancer cell-selective labeling by **pNB-kPin-diol (28)** and **NP-kPin-diol (24)** with **McCDBA**. Scatter plots show fluorescence intensities of calcein AM-stained (a) HEK293T cells under 20% O_2_, (b) HCT116 cells under 20% O_2_, and (c) HCT116 cells under 1% O_2_. In each group, the cells were treated with **McCDBA** alone (50 μM), **McCDBA**+**28** (50 μM each), **McCDBA**+**24** (50 μM each), or DMSO (unt.) in DMEM with 1% DMSO at 37 °C for 30 min and further incubated for 8 h. (d) Fluorescence microscopy images and scatter plots of calcein AM-stained cocultured HEK293T and HCT116 cells treated with **McCDBA** alone (50 μM) or **McCDBA**+**24** (50 μM each) in DMEM with 1% DMSO at 37 °C for 30 min and further incubated under 1% O_2_ for 8 h. The white boxes indicate the zoomed-in views of selected HCT116 and HEK293T cells. The yellow lined enclosed zones indicate the selected HCT116 (blue stars A–C) and HEK293T cells (red stars a, b, and c). The red and blue shaded areas indicate the probable distributions of HEK293T and HCT116 cells, respectively, in the scatter plots. BF channel: bright field; FITC channel: fluorescence of calcein AM; DAPI channel: fluorescence of **McCDBA**; merge channel: fluorescence of **McCDBA** and calcein AM. Scale bars: 50 μm. Each dotted line in the scatter plots represents an individual cell.

Nitroreductases are significant cancer biomarkers for therapeutic and diagnostic research,^50−52^ and we propose that our NTR-responsive cages, **28** and **24**, can differentiate cancer cells from normal cells. We chose HCT116 human colorectal cancer cells as the NTR-overexpressing model^53^ and HEK293T cells as the normal cell model. Both **McCDBA** alone or the two caged **McCDBA** complexes did not exhibit cytotoxicity in an Alamar Blue assay at a working concentration of 50 μM (Figure S15b). Afterward, **McCDBA** alone, **McCDBA**+**28**, and **McCDBA**+**24** were subjected to calcein AM-stained HCT116 or HEK293T cells and incubated under normoxic (20% O_2_) or hypoxic (1% O_2_) conditions. The cellular fluorescence was monitored via fluorescence microscopy and shown as scatter plots: (1) In HEK293T cells, the fluorescence of **McCDBA** was suppressed by **28** or **24** after 8 h of normoxia (Figures 6a and S17a). (2) In HCT116 cells treated with **McCDBA**+**28** under normoxia, a similar suppression of **McCDBA** fluorescence was observed; however, fluorescence recovery occurred in HCT116 cells treated with **McCDBA**+**24** (Figures 6b and S17b). (3) HCT116 cells treated with **McCDBA**+**24** exhibited greater fluorescence recovery under hypoxic conditions compared to normoxic conditions (Figures 6c and S17c). It is to be noted that boronic esters formed by **24** selectively labeled HCT116 cells, even under normoxia. We believe the fluorescence recovery in HCT116 cells under normoxia may be due to oxygen-independent pathways.^54^ Although NTR responsiveness is observed under normoxic conditions but appears enhanced under hypoxia, as indicated by increased fluorescence intensity and changes in cellular fluorescence distribution (Figure S18). Compared with normoxia, cancer cells under hypoxia exhibited significantly stronger fluorescence and greater separation from normal cells in scatter plots, suggesting that hypoxia improves differentiation between cell types and enhances the reduction efficiency of **24**. In contrast, no significant recovery was observed in HCT116 cells treated with **McCDBA**+**28**, suggesting that the boronic esters formed by **28** are poor substrates for the NTR in HCT116 cells.

HCT116 and HEK293T cells were prestained with calcein AM before treatment with either **McCDBA** or **McCDBA**+**24** under hypoxia. We observed different fluorescence labeling efficiencies in both the FITC and DAPI channels (calcein AM and **McCDBA**, respectively) (Figure S19a). HCT116 cells exhibited uneven fluorescence, likely due to intracellular lipid or protein accumulation,^55,56^ while HEK293T cells displayed uniform fluorescence distribution (Figure S19b). In scatter plots, HCT116 and HEK293T cells treated with **McCDBA** alone could not be distinguished. In contrast, cells treated with **McCDBA**+**24** were distinctly separated into two groups (Figure S19c). We also observed distinct morphological differences between HCT116 and HEK293T cells. HCT116 cells displayed an epithelial-like morphology and proliferated through aggregation, while HEK293T cells exhibited a rounded morphology and tended to grow in a dispersed manner under hypoxia (Figure S19b). These differences in fluorescence and morphology facilitated the straightforward sorting of HCT116 and HEK293T cells.

To evaluate the cell selectivity of **McCDBA**+**24** under hypoxia, we cocultured HCT116 and HEK293T cells were cocultured. Treatment with **McCDBA** alone resulted in fluorescence in both cell types, indicating a lack of selectivity. In contrast, fluorescence from **McCDBA**+**24** was selectively observed only in HCT116 cells. Scatter plots (Figure 6d) clearly delineated the two cell populations, allowing the identification of individual HCT116 (blue stars A–C) and HEK293T cells (red stars a–c) within the corresponding regions. Moreover, the distribution patterns in the scatter plots of the cocultured samples were similar to the digitally overlapped scatter plots of HCT116 and HEK293T cells solely treated with **McCDBA**+**24** (Figures 6d and S19c). In summary, these findings demonstrate that our NTR-responsive release strategy exhibits strong selectivity for NTR-overexpressing cancer cells and can be leveraged in studies of intracellular enzymatic reactions involving boronic acids.

## Conclusions

In this work, we reported controllable caging strategies for DBA-based glucose sensing, which respond to specific stimuli, such as UV irradiation or enzymes overexpressed in cancer cells. Unlike common approaches that rely on environmental pH or endogenous diols, our approach provides high spatiotemporal selectivity and the potential to selectively label NTR-overexpressing tumor cells. Additionally, our pinanediol cages allow for the late-stage installation of caged boronic esters, simplifying the synthesis. As a proof of concept, we developed a novel photocontrollable sensor that enhances the spatiotemporal selectivity of intracellular glucose detection and utilizes NTR as a trigger to distinguish cancer cells from normal cells.

Our photoresponsive strategy demonstrates excellent feasibility and precise control, while the NTR-responsive strategy shows potential for detecting differences in hypoxic microenvironments and reflecting nitroreductase expression. However, limitations in photoactivation wavelengths and probe fluorescence spectra pose challenges to tissue penetration. Additionally, although the NTR-responsive strategy reveals differences between cancer/normal cells and hypoxia/normoxia, there is still room for improvement in its sensitivity for further exploration.

Introducing phototriggering units responsive to longer wavelengths would enhance tissue penetration, particularly benefiting studies on animal tumor models. Furthermore, optimizing the structural design of the NTR strategy to improve its responsiveness to NTR or hypoxic environments could provide a valuable tool for researchers exploring cellular heterogeneity such as investigating the cellular behaviors of cancer/normal cell lines derived from the same organ or microenvironments under different hypoxic levels.

On the other hand, we also attempted to apply the pinanediol cages to prodrug strategies. In our preliminary works, we caged two FDA-approved boronic acid-containing anticancer drugs (bortezomib and ixazomib) and achieved photocontrollable drug release in vitro (data not shown). However, we found that the endogenous competitors (e.g., proteasomes) might cause our cages to fail to suppress drug activity. This highlights the challenge of selecting suitable boronic acid targets for activity manipulation. Nevertheless, we successfully developed a design for the stimuli-responsive release of boronic acids. We believe that our strategies can be extended to other bulky diol-based cage scaffolds for other potential boronic acid applications, including controllable hydrogel formation,^57^ saccharide-based single-cell patterning,^58^ and various protein-labeling probes.^25^

## Supporting Information Available

Detailed materials and methods, synthetic procedures and characterization of the synthetic compounds, supplementary figures (PDF)

## References

[ref1] Jabbour A.; Steinberg D.; Dembitsky V. M.; Moussaieff A.; Zaks A. B.; Srebnik M. Synthesis and Evaluation of Oxazaborolidines for Antibacterial Activity against Streptococcus mutans. J. Med. Chem. 2004, 47, 2409–2410. 10.1021/jm049899b. 15115381

[ref2] Yang W.; Gao X.; Wang B. Boronic acid compounds as potential pharmaceutical agents. Med. Res. Rev. 2003, 23, 346–368. 10.1002/med.10043. 12647314

[ref3] Antonio J. P. M.; Russo R.; Carvalho C. P.; Cal P.; Gois P. M. P. Boronic acids as building blocks for the construction of therapeutically useful bioconjugates. Chem. Soc. Rev. 2019, 48, 3513–3536. 10.1039/C9CS00184K. 31157810

[ref4] Williams G. T.; Kedge J. L.; Fossey J. S. Molecular boronic acid-based saccharide sensors. ACS Sens. 2021, 6, 1508–1528. 10.1021/acssensors.1c00462. 33844515 PMC8155662

[ref5] Baker S. J.; Tomsho J. W.; Benkovic S. J. Boron-containing inhibitors of synthetases. Chem. Soc. Rev. 2011, 40, 4279–4285. 10.1039/c0cs00131g. 21298158

[ref6] Plescia J.; Moitessier N. Design and discovery of boronic acid drugs. Eur. J. Med. Chem. 2020, 195, 112270 10.1016/j.ejmech.2020.112270. 32302879

[ref7] Malouff T. D.; Seneviratne D. S.; Ebner D. K.; Stross W. C.; Waddle M. R.; Trifiletti D. M.; Krishnan S. Boron neutron capture therapy: a review of clinical applications. Front. Oncol. 2021, 11, 601820 10.3389/fonc.2021.601820. 33718149 PMC7952987

[ref8] Kim A.; Suzuki M.; Matsumoto Y.; Fukumitsu N.; Nagasaki Y. Non-isotope enriched phenylboronic acid-decorated dual-functional nano-assembles for an actively targeting BNCT drug. Biomater. 2021, 268, 120551 10.1016/j.biomaterials.2020.120551. 33307363

[ref9] Schauenburg D.; Gao B.; Rochet L. N. C.; Schuler D.; Coelho J. A. S.; Ng D. Y. W.; Chudasama V.; Kuan S. L.; Weil T. Macrocyclic Dual-Locked “Turn-On” Drug for Selective and Traceless Release in Cancer Cells. Angew. Chem., Int. Ed. 2024, 63, e202314143 10.1002/anie.202314143. 38179812

[ref10] Manaster A. J.; Batty C.; Tiet P.; Ooi A.; Bachelder E. M.; Ainslie K. M.; Broaders K. E. Oxidation-sensitive dextran-based polymer with improved processability through stable boronic ester groups. ACS Appl. Bio Mater. 2019, 2 (9), 3755–3762. 10.1021/acsabm.9b00399. 35021349

[ref11] Lorand J. P.; Edwards J. O. Polyol complexes and structure of the benzeneboronate ion. J. Org. Chem. 1959, 24 (6), 769–774. 10.1021/jo01088a011.

[ref12] Bachelier N.; Verchere J. F. Formation of neutral complexes of boric acid with 1, 3-diols in organic solvents and in aqueous solution. Polyhedron 1995, 14 (13–14), 2009–2017. 10.1016/0277-5387(94)00451-J.

[ref13] Stubelius A.; Lee S.; Almutairi A. The chemistry of boronic acids in nanomaterials for drug delivery. Acc. Chem. Res. 2019, 52, 3108–3119. 10.1021/acs.accounts.9b00292. 31599160

[ref14] Sun X.; Zhai W.; Fossey J. S.; James T. D. Boronic acids for fluorescence imaging of carbohydrates. Chem. Commun. 2016, 52, 3456–3469. 10.1039/C5CC08633G. 26728041

[ref15] Wu X.; Li Z.; Chen X. X.; Fossey J. S.; James T. D.; Jiang Y. B. Selective sensing of saccharides using simple boronic acids and their aggregates. Chem. Soc. Rev. 2013, 42, 8032–8048. 10.1039/c3cs60148j. 23860576

[ref16] Nan K.; Jiang Y. N.; Li M.; Wang B. Recent Progress in Diboronic-Acid-Based Glucose Sensors. Biosensors 2023, 13 (6), 618 10.3390/bios13060618. 37366983 PMC10296726

[ref17] James T. D.; Sandanayake K. R. A. S.; Shinkai S. Saccharide sensing with molecular receptors based on boronic acid. Angew. Chem., Int. Ed. 1996, 35 (17), 1910–1922. 10.1002/anie.199619101.

[ref18] Norrild J. A fluorescent glucose sensor binding covalently to all five hydroxy groups of α-D-glucofuranose. A reinvestigation. J. Chem. Soc., Perkin Trans. 1999, 3, 449–456. 10.1039/a808896i.

[ref19] Wang K.; Zhang R.; Zhao X.; Ma Y.; Ren L.; Ren Y.; Chen G.; Ye D.; Wu J.; Hu X.; Guo Y.; Xi R.; Meng M.; Yao Q.; Li P.; Chen Q.; James T. D. Reversible recognition-based boronic acid probes for glucose detection in live cells and zebrafish. J. Am. Chem. Soc. 2023, 145, 8408–8416. 10.1021/jacs.2c13694. 37023253 PMC10119935

[ref20] Nascimento R. A.; Özel R. E.; Mak W. H.; Mulato M.; Singaram B.; Pourmand N. Single cell “glucose nanosensor” verifies elevated glucose levels in individual cancer cells. Nano Lett. 2016, 16 (2), 1194–1200. 10.1021/acs.nanolett.5b04495. 26752097 PMC4887140

[ref21] Pliszka M.; Szablewski L. Glucose transporters as a target for anticancer therapy. Cancers 2021, 13 (16), 4184 10.3390/cancers13164184. 34439338 PMC8394807

[ref22] Zou Z.; Luo Z.; Xu X.; Yang S.; Qing Z.; Liu J.; Yang R. Photoactivatable fluorescent probes for spatiotemporal-controlled biosensing and imaging, *TrAC*. Trends Anal. Chem. 2020, 125, 115811 10.1016/j.trac.2020.115811.

[ref23] Drewes G.; Knapp S. Chemoproteomics and chemical probes for target discovery. Trends Biotechnol. 2018, 36 (12), 1275–1286. 10.1016/j.tibtech.2018.06.008. 30017093

[ref24] Bernardini R.; Oliva A.; Paganelli A.; Menta E.; Grugni M.; Munari S. D.; Goldoni L. Stability of boronic esters to hydrolysis: a comparative study. Chem. Lett. 2009, 38, 750–751. 10.1246/cl.2009.750.

[ref25] Akgun B.; Hall D. G. Boronic Acids as Bioorthogonal Probes for Site-Selective Labeling of Proteins. Angew. Chem., Int. Ed. 2018, 57 (40), 13028–13044. 10.1002/anie.201712611. 29723444

[ref26] Yan J.; Springsteen G.; Deeter S.; Wang B. The relationship among pKa, pH, and binding constants in the interactions between boronic acids and diols—it is not as simple as it appears. Tetrahedron 2004, 60, 11205–11209. 10.1016/j.tet.2004.08.051.

[ref27] Brieke C.; Rohrbach F.; Gottschalk A.; Mayer G.; Heckel A. Light-controlled tools. Angew. Chem., Int. Ed. 2012, 51 (34), 8446–8476. 10.1002/anie.201202134. 22829531

[ref28] Patterson A. V.; Saunders M. P.; Chinje E. C.; Talbot D. C.; Harris A. L.; Strafford I. J. Overexpression of human NADPH: cytochrome c (P450) reductase confers enhanced sensitivity to both tirapazamine (SR 4233) and RSU 1069. Br. J. Cancer 1997, 76 (10), 1338–1347. 10.1038/bjc.1997.558. 9374381 PMC2228151

[ref29] Wilson W. R.; Hicks K. O.; Pullen S. M.; Ferry D. M.; Helsby N. A.; Patterson A. V. Bystander effects of bioreductive drugs: potential for exploiting pathological tumor hypoxia with dinitrobenzamide mustards. Radiat. Res. 2007, 167 (6), 625–636. 10.1667/RR0807.1. 17523848

[ref30] Wilson W. R.; Hay M. P. Targeting hypoxia in cancer therapy. Nat. Rev. Cancer 2011, 11 (6), 393–410. 10.1038/nrc3064. 21606941

[ref31] Liu F.; Zhang H.; Li K.; Xie Y.; Li Z. A novel NIR fluorescent probe for highly selective detection of nitroreductase and hypoxic-tumor-cell imaging. Molecules 2021, 26 (15), 4425 10.3390/molecules26154425. 34361578 PMC8347683

[ref32] Chen Y.; Zhang X.; Lu X.; Wu H.; Zhang D.; Zhu B.; Huang S. Ultra-sensitive responsive near-infrared fluorescent nitroreductase probe with strong specificity for imaging tumor and detecting the invasiveness of tumor cells. Spectrochim. Acta. -A: Mol. Biomol. Spectrosc. 2022, 268, 120634 10.1016/j.saa.2021.120634. 34836811

[ref33] Kong F.; Li Y.; Yang C.; Li X.; Wu J.; Liu X.; Tang B. A fluorescent probe for simultaneously sensing NTR and hNQO1 and distinguishing cancer cells. J. Mater. Chem. 2019, 7 (43), 6822–6827. 10.1039/C9TB01581G. 31608921

[ref34] Cadahía J. P.; Previtali V.; Troelsen N. S.; Clausen M. H. Prodrug strategies for targeted therapy triggered by reactive oxygen species. Med. Chem. Commun. 2019, 10, 1531–1549. 10.1039/C9MD00169G. PMC6786010 31673314

[ref35] Roy C. D.; Brown H. C. A comparative study of the relative stability of representative chiral and achiral boronic esters employing transesterification. Monatsh. Chem. 2007, 138, 879–887. 10.1007/s00706-007-0699-x.

[ref36] Fan N. C.; Cheng F. Y.; Ho J. A.; Yeh C. S. Photocontrolled targeted drug delivery: photocaged biologically active folic acid as a light-responsive tumor-targeting molecule. Angew. Chem., Int. Ed. 2012, 51, 8806–8810. 10.1002/anie.201203339. 22833461

[ref37] Okamura H.; Iida M.; Kaneyama Y.; Nagatsugi F. *o*-Nitrobenzyl Oxime Ethers Enable Photoinduced Cyclization Reaction to Provide Phenanthridines under Aqueous Conditions. Org. Lett. 2023, 25, 466–470. 10.1021/acs.orglett.2c04015. 36629406

[ref38] Cui L.; Zhong Y.; Zhu W.; Xu Y.; Du Q.; Wang X.; Qian X.; Xiao Y. A new prodrug-derived ratiometric fluorescent probe for hypoxia: high selectivity of nitroreductase and imaging in tumor cell. Org. Lett. 2011, 13 (5), 928–931. 10.1021/ol102975t. 21268631

[ref39] Luo S.; Zou R.; Wu J.; Landry M. P. A probe for the detection of hypoxic cancer cells. ACS Sens. 2017, 2, 1139–1145. 10.1021/acssensors.7b00171. 28741347 PMC10494911

[ref40] Antermite D.; Friis S. D.; Johansson J. R.; Putra O. D.; Ackermann L.; Johansson M. J. Late-stage synthesis of heterobifunctional molecules for PROTAC applications via ruthenium-catalysed C–H amidation. Nat. Commun. 2023, 14, 8222 10.1038/s41467-023-43789-9. 38086825 PMC10716378

[ref41] Achilli C.; Ciana A.; Fagnoni M.; Balduini C.; Minetti G. Susceptibility to hydrolysis of phenylboronic pinacol esters at physiological pH. Open Chem. 2013, 11, 137–139. 10.2478/s11532-012-0159-2.

[ref42] Michael N. P.; Brehm J. K.; Anlezark G. M.; Minton N. P. Physical characterisation of the Escherichia coli B gene encoding nitroreductase and its over-expression in Escherichia coli K12. FEMS Microbiol. Lett. 1994, 124 (2), 195–202. 10.1111/j.1574-6968.1994.tb07284.x. 7813889

[ref43] Anlezark G. M.; Melton R. G.; Sherwood R. F.; Coles B.; Friedlos F.; Knox R. J. The bioactivation of 5-(aziridin-1-yl)-2,4-dinitrobenzamide (CB1954)—I: Purification and properties of a nitroreductase enzyme from Escherichia coli—A potential enzyme for antibody-directed enzyme prodrug therapy (ADEPT). Biochem. Pharmacol. 1992, 44 (12), 2289–2295. 10.1016/0006-2952(92)90671-5. 1472094

[ref44] Zhao D.; Xu J. Q.; Yi X. Q.; Zhang Q.; Cheng S. X.; Zhuo R. X.; Li F. pH-activated targeting drug delivery system based on the selective binding of phenylboronic acid. ACS Appl. Mater. Interfaces. 2016, 8, 14845–14854. 10.1021/acsami.6b04737. 27229625

[ref45] Zhu J.; Huo Q.; Xu M.; Yang F.; Li Y.; Shi H.; Niu Y.; Liu Y. Bortezomib-catechol conjugated prodrug micelles: combining bone targeting and aryl boronate-based pH-responsive drug release for cancer bone-metastasis therapy. Nanoscale 2018, 10, 18387–18397. 10.1039/C8NR03899F. 30256367

[ref46] Hu X.; Chai Z.; Lu L.; Ruan H.; Wang R.; Zhan C.; Xie C.; Pan J.; Liu M.; Wang H.; Lu W. Bortezomib dendrimer prodrug-based nanoparticle system. Adv. Funct. Mater. 2019, 29 (14), 1807941 10.1002/adfm.201807941.

[ref47] Arimori S.; Bell M. L.; Oh C. S.; Frimat K. A.; James T. D. Modular fluorescence sensors for saccharides. J. Chem. Soc., Perkin Trans. 2002, 6, 803–808. 10.1039/b108998f. 12240339

[ref48] Bosch L. I.; Fyles T. M.; James T. D. Binary and ternary phenylboronic acid complexes with saccharides and Lewis bases. Tetrahedron 2004, 60 (49), 11175–11190. 10.1016/j.tet.2004.08.046.

[ref49] Tang J.; Ma D.; Pecic S.; Huang C.; Zheng J.; Li J.; Yang R. Noninvasive and highly selective monitoring of intracellular glucose via a two-step recognition-based nanokit. Anal. Chem. 2017, 89, 8319–8327. 10.1021/acs.analchem.7b01532. 28707883

[ref50] Qi Y. L.; Guo L.; Chen L. L.; Li H.; Yang Y. S.; Jiang A. Q.; Zhu H. L. Recent progress in the design principles, sensing mechanisms, and applications of small-molecule probes for nitroreductases. Coord. Chem. Rev. 2020, 421, 213460 10.1016/j.ccr.2020.213460.

[ref51] Shang B.; Yu Z.; Wang Z. Recent advances and applications of nitroreductase activable agents for tumor theranostic. Front. Pharmacol. 2024, 15, 1451517 10.3389/fphar.2024.1451517. 39101150 PMC11294179

[ref52] Boddu R. S.; Perumal O.; K D. Microbial nitroreductases: A versatile tool for biomedical and environmental applications. Biotechnol. Appl. Biochem. 2021, 68 (6), 1518–1530. 10.1002/bab.2073. 33156534

[ref53] Li T.; Gu Q. S.; Chao J. J.; Liu T.; Mao G. J.; Li Y.; Li C. Y. An intestinal-targeting near-infrared probe for imaging nitroreductase in inflammatory bowel disease. Sens. Actuators B: Chem. 2024, 403, 135181 10.1016/j.snb.2023.135181.

[ref54] Sebestyen A.; Kopper L.; Danko T.; Timar J. Hypoxia signaling in cancer: from basics to clinical practice. Pathol. Oncol. Res. 2021, 27, 1609802 10.3389/pore.2021.1609802. 34257622 PMC8262153

[ref55] Lee H. M.; Lee S. C.; He L.; Kong A. P. S.; Mao D.; Hou Y.; Chung A. C. K.; Xu G.; Ma R. C. W.; Chan J. C. N. Legacy effect of high glucose on promoting survival of HCT116 colorectal cancer cells by reducing endoplasmic reticulum stress response. Am. J. Cancer Res. 2021, 11 (12), 6004. 35018239 PMC8727802

[ref56] Hsu J. F.; Hsieh P. Y.; Hsu H. Y.; Shigeto S. When cells divide: Label-free multimodal spectral imaging for exploratory molecular investigation of living cells during cytokinesis. Sci. Rep. 2015, 5, 17541 10.1038/srep17541. 26632877 PMC4668386

[ref57] Banach Ł.; Williams G. T.; Fossey J. S. Insulin delivery using dynamic covalent boronic acid/ester-controlled release. Adv. Ther. 2021, 4 (11), 2100118 10.1002/adtp.202100118.

[ref58] Liu H.; Li Y.; Sun K.; Fan J.; Zhang P.; Meng J.; Wang S.; Jiang L. Dual-responsive surfaces modified with phenylboronic acid-containing polymer brush to reversibly capture and release cancer cells. J. Am. Chem. Soc. 2013, 135, 7603–7609. 10.1021/ja401000m. 23601154

