# Supplementary material for: Versatile Stimuli-Responsive Controlled Release of Pinanediol-Caged Boronic Esters for Spatiotemporal and Nitroreductase-Selective Glucose Bioimaging
Source: ACS Sens. 2025 Jan 3;10(1):470–9. doi: 10.1021/acssensors.4c02811 (PMC11773560; doi:10.1021/acssensors.4c02811)
Supplement: Supplementary file 1 — se4c02811_si_001.pdf [file se4c02811_si_001.pdf]

Supporting Information

Versatile Stimuli-Responsive Controlled Release of Pinanediol-Caged Boronic Esters for Spatiotemporal and Nitroreductase-Selective Glucose Bio-imaging

Chih-Yao Kao,[a] Ying-Wei Chen,[a] Yu-Cheng Liu,[b] Jen-Hsuan Wei,[b] Tsung-Shing Andrew Wang*[a]

[a]Department of Chemistry and Center for Emerging Material and Advanced Devices, National Taiwan University, Taipei, 106319, Taiwan (R.O.C.) E-mail: wangts@ntu.edu.tw.

[b]Institute of Molecular Biology, Academia Sinica, Nankang, Taipei, 115201, Taiwan (R.O.C.)

Table of contents

1. General Methods and Instrument 4
2. Synthesis and Characterization of Compounds 5
3. Hydrolysis, Assembly and Fluorescence Detection Assays 22

General methods 22

Hydrolysis and assembly assays 22

Fluorescence detection with McCDBA and pinanediol cages 23

4. Determination of the Binding Constants 24
5. Cell Experiments 27
6. Supporting Schemes and Figures 30

Scheme S1...30

Scheme S2...31

Scheme S3...32

Scheme S4...32

Scheme S5...33

Scheme S6...34

Figure S1. 35

Figure S2. 36

Figure S3. 37

Figure S4. 38

Figure S5. 38

Figure S6. 39

Figure S7. 39

Figure S8. 40

Figure S9. 41

Figure S10. 42

Figure S11. 43
Figure S12. 43
Figure S13. 44
Figure S14. 44
Figure S15. 45
Figure S16. 45
Figure S17. 46
Figure S18. 47
Figure S19. 48
7. Appendix: NMR Spectroscopic Data 49
8. References 75

1. General Methods and Instrument

Synthetic Materials. All chemicals used in the experiments were purchased from Alfa Aesar, AK Scientific, Acros, ECHO Chemical, Merck, Nova Materials, Showa, and Thermo Fisher Scientific. The anhydrous solvent used in the experiment were obtained from solvent purification system (LC Technology Solutions Inc.). Dulbecco's Modified Eagle Medium (DMEM), DMEM (without glucose), McCoy's 5A (Modified) medium, and Roswell Park Memorial Institute (RPMI) 1640 medium was purchased from GIBCO® (Thermo Fisher Scientific). Reactions were monitored by pre-coated thin layer chromatography (TLC) plates (Merck, 0.25mm silica gel 60 F_{254}), and compounds were visualized by UV lamps, phosphomolybdic acid (PMA), potassium permanganate ($KMnO_4$), and ninhydrin. The flash column chromatography was purchased from Merck Geduran silica gel 60 (particle size: 0.040-0.063 mm). The eluents used in flash column chromatography were the mixtures containing hexane and ethyl acetate.

Instrumentation. The high-performance liquid chromatography was performed on either Agilent 1260 infinity quaternary LC system or Agilent 1100 Series LC system. The purification was performed on a semi-preparative column (YMC-Triart C18, 5 µm pore size, 250x10 mm), and the analyses were performed on either YMC-Triart C18 column (5 µm pore size, 4.6x250 mm) or Agilent Eclipse XDB-C18 column (5 µm pore size, 4.6x150 mm). All HRMS experiments was performed on Dionex Ultimate 3000 UHPLC coupled with a Bruker maxis mass spectrometer. The NMR spectrum were obtained with either Bruker AVIII HD 400MHz or Varian Unity Plus-400 (1H: 400 MHz, 13C: 100 MHz, 19F: 376 MHz). Chemical shifts (δ) were recorded in part per million (ppm), the residuals of non-deuterated solvents were used as references. Coupling constants (J) were recorded in hertz (Hz), and the splitting patterns were reported as s (singlet), d (doublet), t (triplet), q (quartet), dd (double doublet), dt (double triplet), td (triple doublet), qd (quartet doublet), ddd (double double doublet), qdd (quartet double doublet), and m (multiplet). The microscopic images were taken using ImageXpress Pico Automated Cell Imaging System and Zeiss LSM 980. The 365 nm LED (16 LED chips arranged in a 4x4 array inside a 1.5 cmx1.5 cm square, 50 W total) was purchased from KOODYZ Technology (Taiwan).

2. Synthesis and Characterization of Compounds

All reactions and purifications of o-nitrobenzyl moiety-containing compounds were carried out under controlled light conditions to avoid unwanted photolysis of the products.

Synthesis of tPin-EtPh (2)

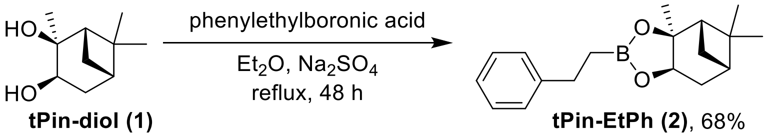

To a mixture of (1*S*,2*S*,3*R*,5*S*)-(+)-Pinanediol (**tPin-diol (1)**) (200 mg, 1.2 mmol, 1.0 equiv.) and phenylethylboronic acid (180 mg, 1.2 mmol, 1.0 equiv.) in anhydrous Et_2O (1.2 mL) was added excess Na_2SO_4 (1 g). The mixture was stirred for 48 hours at reflux temperature. After filtration to remove Na_2SO_4, the solid was washed with CH_2Cl_2 for 3 times. The combined filtrate was concentrated under reduced pressure, and purified by flash column chromatography (Hexane/EtOAc = 4/1) to give **tPin-EtPh (2)** (228 mg, 68%) as a colorless oil. R_f =0.75 (Hexane/EtOAc = 4/1). ^{1}H NMR (400 MHz, $CDCl_3$) δ 7.31 – 7.24 (m, 4H), 7.20-7.16 (m, 1H), 4.28 (dt, *J* = 8.7, 1.9 Hz, 1H), 2.80(t, *J* = 8.7Hz, 2H), 2.40 – 2.31 (m, 1H), 2.22-2.15 (m, 1H), 2.06 (td, *J* = 5.4, 1.6 Hz, 1H), 1.94 – 1.89 (m, 1H), 1.85 (ddd, *J* = 14.5, 5.2, 1.4 Hz, 1H), 1.39 (d, *J* = 1.7 Hz, 3H), 1.31 (s,3H), 1.22(t, *J* = 8.7Hz,2H), 1.04 (dd, *J* = 11.0, 1.6 Hz, 1H), 0.86 (s, 3H). ^{13}C NMR (100 MHz, $CDCl_3$) δ 144.54, 128.34, 128.14, 125.65, 85.68, 77.83, 51.40, 39.65, 38.26, 35.59, 30.25, 29.85, 28.80, 27.22, 26.52, 24.14. HRMS (ESI, m/z): $[M+H]^+$, calculated: 285.2024, found: 285.2022.

Synthesis of pinanol (3)

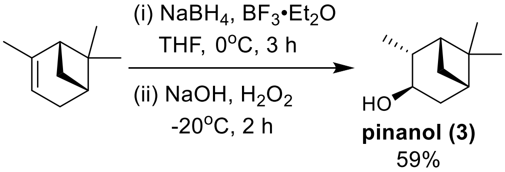

$NaBH_4$ (28 mg, 0.73 mmol, 1.0 equiv.) was dried under reduced pressure. A solution of α-pinene (100 mg, 0.73 mmol, 1.0 equiv.) in THF (0.25 mL) was added at 0°C, followed by dropwise addition of $BF_3 \cdot Et_2O$ (0.087 mL, 0.73 mmol, 1.0 equiv.) at 0°C. After stirring for 3 hours at 0°C, 3M $NaOH_{(aq)}$ (0.25

mL) and H_2O_2 (0.37 mL) was added dropwise at -20°C. The mixture was further stirred for another 2 hours. After the reaction completed, the mixture was diluted with CH_2Cl_2 and washed with $NaHCO_{3(sat.)}$ for 3 times. The combined organic layer was dried over $MgSO_4$, filtered, and then concentrated under reduced pressure. The residue was purified by flash column chromatography (Hexane/EtOAc = 6/1) to give **pinenol (3)** (67 mg, 59%) as a white solid. R_f = 0.28 (Hexane/EtOAc = 6/1). ^{1}H NMR (400 MHz, $CDCl_3$) δ 4.06 (dt, *J* = 9.5, 5.0 Hz, 1H), 2.55 - 2.47 (m, 1H), 2.40 – 2.34 (m, 1H), 1.96 – 1.89 (m, 2H), 1.80 (td, *J* = 5.9, 2.0 Hz, 1H), 1.70 (ddd, *J* = 14.2, 4.7, 2.6 Hz, 1H), 1.22 (s, 3H), 1.13 (d, *J* = 7.3 Hz, 3H), 1.03 (d, *J* = 9.8 Hz, 1H), 0.92 (s, 3H). ^{13}C NMR (100 MHz, $CDCl_3$) δ 71.84, 47.98, 47.92, 41.92, 39.19, 38.30, 34.55, 27.81, 23.83, 20.88. HRMS (ESI, m/z): $[M+H]^+$, calculated: 155.1430, found: 155.1421

Synthesis of δ-pinene (5)

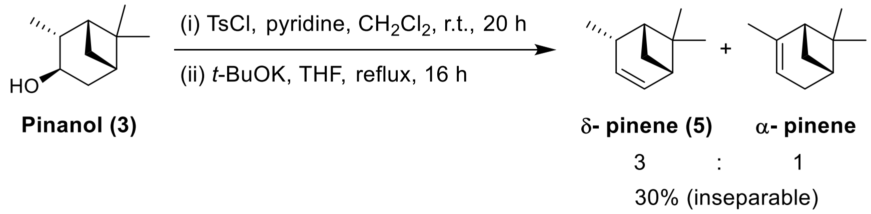

δ-Pinene (5) was synthesized as previously reported.[3] A mixture of regioisomers were obtained (**δ-pinene (5)**: **α-pinene** = 3:1) according to the NMR spectral data. ^{1}H NMR (400 MHz, $CDCl_3$) δ 6.10 (ddd, *J* = 8.8, 6.2, 2.3 Hz, 1H), 5.53 (d, *J* = 8.8 Hz, 1H), 5.20 – 5.18 (m, 1H), 2.67 – 2.61 (m, 1H), 2.45 (dt, *J* = 8.5, 5.7 Hz, 1H), 2.33 (dt, *J* = 8.4, 5.6 Hz, 1H), 2.26 – 2.13 (m, 1H), 2.12 – 2.01 (m, 3H), 1.93 (td, *J* = 5.7, 1.5 Hz, 1H), 1.66 (d, *J* = 2.0 Hz, 1H), 1.31 (d, *J* = 5.6 Hz, 1H), 1.29 (s, 3H), 1.27 (s, 1H), 1.15 (d, *J* = 8.4 Hz, 1H), 1.09 (d, *J* = 7.6 Hz, 3H), 1.00 (s, 3H), 0.84 (s, 1H). ^{13}C NMR (100 MHz, CDCl3) δ 144.68, 134.53, 129.81, 116.17, 48.37, 47.18, 42.23, 40.88, 40.46, 38.12, 38.05, 35.36, 31.61, 31.42, 27.53, 26.51, 23.91, 23.14, 20.95, 18.51.

Synthesis of kPin-diol (6)

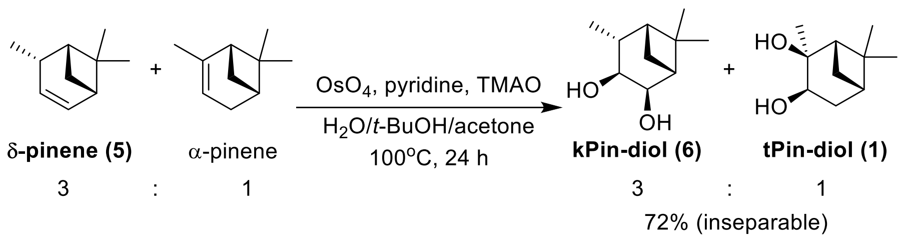

In a mixture of pinene (**δ-pinene (5)**: α-pinene = 3:1, 20 mg, 0.15 mmol, 1.0 equiv.) in *tert*-butanol (2.5 mL), acetone (1.3 mL), water (0.63 mL), trimethylamine N-oxide (23 mg, 0.30 mmol, 2.0 mmol), pyridine (12 uL), and 2.5% Osmium tetroxide in *tert*-butanol (47 uL) were added. The mixture was stirred for 24 hours at 100°C and the color of the mixture was observed turning to be dark. After cooling the mixture to room temperature, sodium sulfite (19 mg, 0.15 mmol, 1 equiv.) was added to quench osmium tetroxide. The mixture was stirred for further 30 minutes, followed by filtration through a pad of Celite. The filtrate was dried over $MgSO_4$, filtered, and concentrated under reduced pressure. The residue was purified by flash column chromatography (Hexane/EtOAc = 2/1) to give a mixture of pinanediol (**kPin-diol (6)**: **tPin-diol (1)** = 3:1, 18 mg, 72% yield) as a colorless oil. R_f = 0.20 (Hexane/EtOAc = 2/1) ^{1}H NMR (400 MHz, $CDCl_3$) δ 4.26 (ddd, *J* = 7.8, 3.6, 1.9 Hz, 1H), 4.02 – 3.95 (m, 1H), 2.83 (s, 2H), 2.49 – 2.41 (m, 1H), 2.23 – 2.17 (m, 3H), 2.00 (t, *J* = 5.8 Hz, 1H), 1.94 – 1.84 (m, 2H), 1.81 (td, *J* = 6.0, 2.1 Hz, 1H), 1.63 (ddd, *J* = 14, 5.2, 2.4 Hz, 1H), 1.36 (d, *J* = 10.4 Hz, 1H), 1.30 (s, 1H), 1.26 (s, 4H), 1.15 (d, *J* = 7.2 Hz, 3H), 0.95 (s, 3H), 0.93 (s, 1H). ^{13}C NMR (100 MHz, $CDCl_3$) δ 74.03, 72.00, 69.92, 69.40, 54.13, 48.16, 47.75, 46.76, 40.66, 39.35, 39.13, 38.32, 29.70, 28.40, 28.16, 28.13, 27.96, 24.26, 23.34, 20.74. HRMS (ESI, m/z): $[M+Na]^+$, calculated: 193.1199, found: 193.1187

Synthesis of kPin-EtPh (7)

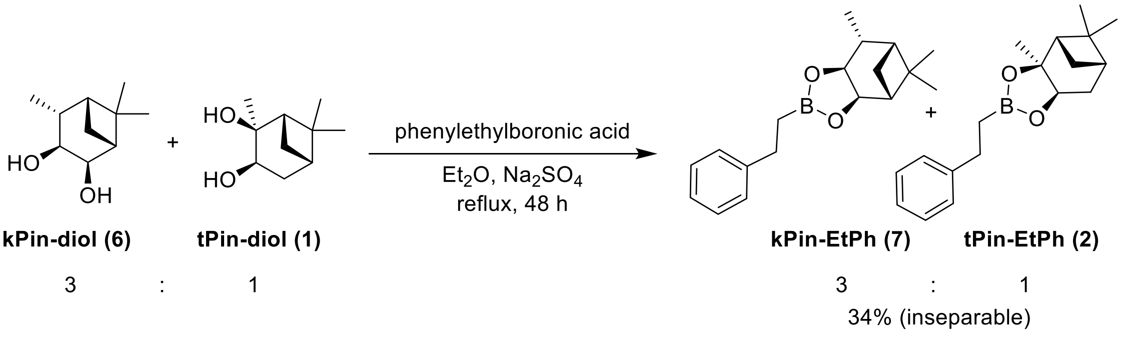

A mixture of pinanediol (**kPin-diol (6)**: **tPin-diol (1)** = 3:1, 20 mg, 0.12 mmol, 1.0 equiv.) and

phenylethylboronic acid (18 mg, 0.12 mmol, 1.0 equiv.) in anhydrous Et_2O (1.2 mL) was added excess Na_2SO_4 (1 g). The mixture was stirred for 48 hours at reflux temperature. After filtration to remove Na_2SO_4, the solid was washed with CH_2Cl_2 for 3 times. The combined filtrate was concentrated under reduced pressure, and purified by flash column chromatography (Hexane/EtOAc = 4/1, R_f =0.75). Next, the product was further purified by reverse phase HPLC to give a mixture pinanediol boronic ester (**kPin-EtPh (7)**: **tPin-EtPh (2)** = 3:1, 11.2 mg, 34% yield) as a colorless oil. HPLC method: column, YMC-Triart C18 (5 µm pore size, 250x10 mm); flow rate, 3.0 mL/min; wavelength,254 nm; mobile phase, water and ACN. Elution gradient was: 0-6 min, 100% water; 6-18min, 100% water–100% ACN; 18-30 min, 100% ACN

^{1}H NMR (400 MHz, $CDCl_3$) δ 7.28 – 7.20 (m, 6H), 7.18 – 7.13 (m, 1H), 4.74 (ddd, *J* = 8.4, 3.5, 1.6 Hz, 1H), 4.39 (dd, *J* = 8.3, 3.1 Hz, 1H), 4.25 (dd, *J* = 8.7, 2.0 Hz, 1H), 2.77 (t, *J* = 8.2 Hz, 3H), 2.36 – 2.29 (m, 1H), 2.22 – 2.11 (m, 2H), 2.09 – 2.02 (m, 1H), 1.91 – 1.87 (m, 1H), 1.84 – 1.80 (m, 1H), 1.36 (s, 1H), 1.28 (s, 4H), 1.20 (t, *J* = 8.2 Hz, 3H), 1.16 (d, *J* = 7.6 Hz, 3H), 1.00 (d, *J* = 10.9 Hz, 1H), 0.92 (d, *J* = 10.4 Hz, 1H), 0.90 (s, 3H), 0.84 (s, 1H). ^{13}C NMR (100 MHz, $CDCl_3$) δ 144.55, 128.38, 128.35, 128.15, 128.11, 125.67, 85.69, 80.34, 79.82, 77.82, 51.38, 47.04, 45.29, 44.55, 39.63, 39.02, 38.26, 35.59, 30.24, 30.21, 28.79, 27.43, 27.21, 26.76, 26.51, 24.14, 23.11, 20.77. HRMS (ESI, m/z): $[M+Na]^+$, calculated: 307.1843, found: 307.1848

Synthesis of oNB-Br (8)

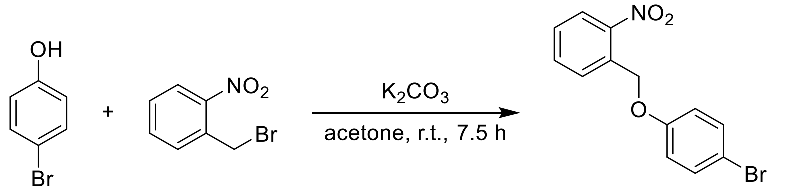

oNB-Br (8), 79%

To a solution of 2-nitrobenzyl bromide (210 mg, 0.96 mmol, 1.0 equiv.) and 4-bromophenol (500 mg, 2.9 mmol, 3.0 equiv.) in acetone (1.9 mL) was added potassium carbonate (266 mg, 1.9 mmol, 2.0 equiv.). The mixture was stirred for 7.5 hours at room temperature. After the reaction completed, the mixture was extracted with EtOAc and brine for 3 times. The combined organic layer was dried over $MgSO_4$, filtered, and concentrated under reduced pressure. The residue was purified by flash

column chromatography (Hexane/EtOAc = 4/1) to give **oNB-Br (8)** (238 mg, 79%) as a white solid. R*f* = 0.55 (Hexane/EtOAc = 4/1). ^{1}H NMR (400 MHz, $CDCl_3$) δ 8.17 (d, *J* = 8.2 Hz, 1H), 7.85 (d, *J* = 7.8 Hz, 1H), 7.69 (dd, *J* = 7.8, 7.6 Hz, 1H), 7.50 (dd, *J* = 8.2, 7.6 Hz, 1H), 7.40 (d, *J* = 9.0 Hz, 2H), 6.88 (d, *J* = 9.0 Hz, 2H), 5.47 (s, 2H). ^{13}C NMR (100 MHz, $CDCl_3$) δ 157.22, 146.97, 134.02, 133.36, 132.49, 128.47, 125.06, 116.69, 113.78, 67.04. HRMS (ESI, m/z): $[M+H]^+$, calculated: 307.9917, found: 308.0012

Synthesis of oNB-Pinacol (9)

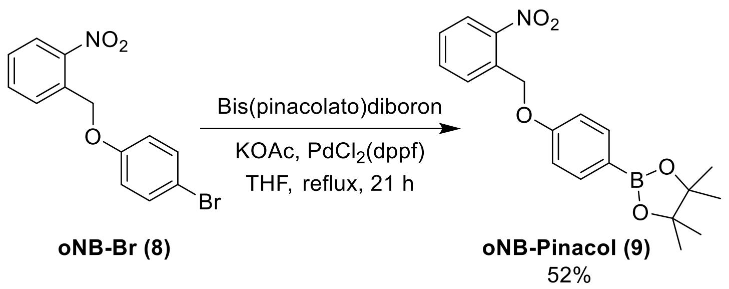

To a mixtrure of **oNB-Br (8)** (100 mg, 0.32 mmol, 1.0 equiv.), bis(pinacolato)diboron (200 mg, 0.87 mmol, 2.7 equiv.), potassium acetate (64 mg, 0.65mmol, 2.0 equiv.), and $PdCl_2$(dppf) (12 mg, 0.016 mmol, 0.05 equiv.) was added THF (1.6 mL). The mixture was stirred for 21 hours at reflux temperature. Noteworthy, the color of the mixture was observed turning to dark during the reaction. After the reaction completed, the mixture was extracted with EtOAc and brine for 3 times. The combined organic layer was dried over $MgSO_4$, filtered, and concentrated under reduced pressure. The residue was purified by flash column chromatography (Hexane/EtOAc = 19/1) to give **oNB-Pinacol (9)** (60 mg, 52%) as a white solid. R*f* =0.40 (Hexane/EtOAc = 19/1). ^{1}H NMR (400 MHz, $CDCl_3$) δ 8.17 (d, *J* = 8.2 Hz, 1H), 7.88 (d, *J* = 7.8 Hz, 1H), 7.77 (d, *J* = 8.6 Hz, 2H), 7.67 (dd, *J* = 7.8, 7.6 Hz, 1H), 7.48 (dd, *J* = 8.2, 7.6 Hz, 1H), 6.98 (d, *J* = 8.6 Hz, 2H), 5.53 (s, 2H), 1.34 (s, 12H). ^{13}C NMR (100 MHz, $CDCl_3$) δ 160.56, 146.84, 136.65, 134.03, 133.79, 128.47, 128.29, 125.00, 114.17, 83.64, 66.46, 24.84. HRMS (ESI, m/z): $[M+H]^+$, calculated: 356.1667, found: 356.1664

Synthesis of pinanone (10)

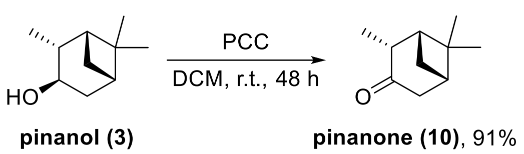

Pyridinium chlorochromate (84 mg, 0.39 mmol, 3.0 equiv.) was dried under reduced pressure, and **pinanol (3)** (20 mg, 0.13 mmol, 1.0 equiv.) in CH_2Cl_2 (70 uL) was added slowly at 0°C. The mixture warmed to room temperature then stirred for 48 hours. After the reaction completed, the mixture was filtered through a pad of Celite then washed with CH_2Cl_2 for 3 times. The combined filtrate was further washed with $NaHCO_{3(aq)}$ for 3 times, and the organic layer was concentrated under reduced pressure, to give **pinanone (10)** (18 mg, 91%) as a color less oil. **pinanone (10)** can be removed in a high-pressure vacuum system, so only rotavapor was used to remove the solvent, and some DCM remains in this step. R_f = 0.58 (Hexane/EtOAc = 6/1). ^{1}H NMR (400 MHz, $CDCl_3$) δ 2.67 – 2.59 (m, 2H), 2.54 – 2.53 (m, 1H), 2.49 – 2.43 (m, 1H), 2.14 – 2.11 (m, 1H), 2.05 (td, *J* = 6.3, 1.9 Hz, 1H), 1.31 (s, 3H), 1.21 (d, *J* = 7.3, 3H), 1.18 (d, *J* = 10, 1H), 0.87 (s, 3H). ^{13}C NMR (100 MHz, $CDCl_3$) δ 215.32, 51.45, 45.15, 44.89, 39.33, 39.10, 34.50, 27.16, 22.05, 16.95. HRMS (ESI, m/z): $[M+H]^+$, calculated: 153.1274, found: 153.1265

Synthesis of OTf-tPin (11)

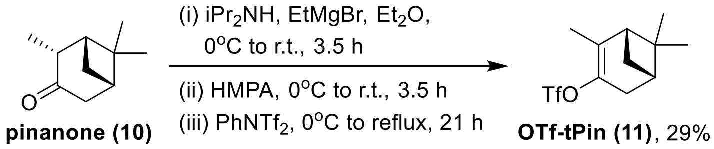

To a dried round-bottom flask was added anhydrous diisopropylamine (0.70 mL, 4.9 mmol, 1.5 equiv.) and anhydrous Et_2O (5.5 mL), followed by dropwise addition of 3.0 M ethylmagnesium bromide in Et_2O (1.6 mL, 4.9 mmol, 1.5 equiv.) at 0°C. After stirring for 30 minutes at 0°C, the mixture was warmed to room temperature then stirred for further 3 hours. The mixture was cooled to 0°C then added HMPA (1.6 mL, 8.9 mmol, 2.7 equiv.) and **pinanone (10)** (0.50 g, 3.3 mmol, 1.0 equiv.). After stirring for 30 minutes at 0°C, the mixture was warmed to room temperature then stirred for further 3 hours. The mixture was added bis(trifluoromethanesulfonyl)aniline (1.2 g, 3.3 mmol, 1.0

equiv.) as powder in a portion then stirred for 13 hours at room temperature, followed by reflux for 8 hours. After the reaction completed, the mixture was extracted with EtOAc and 1N HCl. The combined organic layer was dried over $MgSO_4$, filtered, concentrated under reduced pressure, and purified by flash column chromatography with Hexane to give **OTf-tPin (11)** (275 mg, 29%) as a colorless oil. R_f =0.40 (hexanes 100%). ^{1}H NMR (400 MHz, $CDCl_3$) δ 2.62 – 2.51 (m, 2H), 2.41 (dt, *J* = 9.2, 5.4 Hz, 1H), 2.25 – 2.21 (m, 1H), 2.08 (dd, *J* = 6.7, 5.4 Hz, 1H), 1.77 (t, *J* = 2.2 Hz, 3H), 1.30 (s, 3H), 1.28 (d, *J* = 9.2 Hz, 1H), 0.92 (s, 3H). ^{13}C NMR (100 MHz, $CDCl_3$) δ 140.91, 135.77, 120.17, 116.99, 46.53, 41.06, 39.73, 33.31, 31.67, 26.01, 20.49, 16.85. ^{19}F NMR (376 MHz, $CDCl_3$) δ -74.77. HRMS (ESI, m/z): $[M+Na]^+$, calculated: 307.0586, found: 307.0614

Synthesis of oNB-tPin (12)

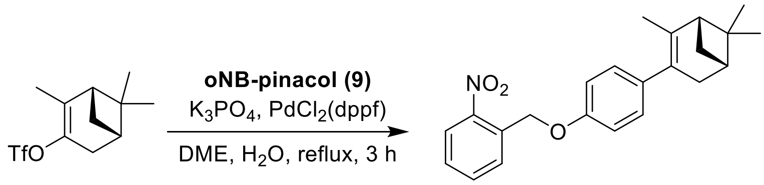

OTf-tPin (11) **oNB-tPin (12)**, 31%

A mixture of **OTf-tPin (11)** (50 mg, 0.18 mmol, 1.0 equiv.), **oNB-pinacol (9)** (78 mg, 0.22 mmol, 1.2 equiv.), potassium phosphate (76 mg, 0.36 mmol, 2.0 equiv.), and $PdCl_2$(dppf) (13 mg, 0.018 mmol, 0.10 equiv.) in dimethoxyethane (1.8 mL) and water (0.36 mL) was stirred for 3 hours at 100°C. After the reaction completed, the mixture was extracted with EtOAc and brine for 3 times. The combined organic layer was dried over $MgSO_4$, filtered, concentrated under reduced pressure, and purified by silica column chromatography (Hexanes/EtOAc = 19/1) to give **oNB-tPin (12)** (20 mg, 31%) as a yellow oil. R_f =0.48 (Hexanes/EtOAc = 19/1). ^{1}H NMR (400 MHz, $CDCl_3$) δ 8.17 (d, *J* = 8.2 Hz, 1H), 7.93 (d, *J* = 7.8 Hz, 1H), 7.69 (dd, *J* = 7.8, 7.6 Hz, 1H), 7.49 (dd, *J* = 8.2, 7.6 Hz, 1H), 7.18 (d, *J* = 8.7 Hz, 2H), 6.95 (d, *J* = 8.7 Hz, 2H), 5.50 (s, 2H), 2.52 – 2.47 (m, 2H), 2.40 (dt, *J* = 5.6, 2.6 Hz, 1H), 2.20 – 2.17 (m, 1H), 2.08 (dd, *J* = 6.3, 5.6 Hz, 1H), 1.69 (t, *J* = 2.0 Hz, 3H), 1.31 (s, 3H), 1.29 (d, *J* = 8.7 Hz, 1H), 0.92 (s, 3H). ^{13}C NMR (100 MHz, $CDCl_3$) δ 156.51, 147.02, 139.35, 135.49, 134.37, 134.13, 129.58, 128.77, 128.37, 126.79, 125.09, 114.57, 67.06, 48.18, 41.24, 38.75, 36.94, 31.50, 26.46, 20.92, 20.88. HRMS (ESI, m/z): $[M+H]^+$, calculated: 364.1907, found: 364.1843

Synthesis of oNB-tPin-diol (13)

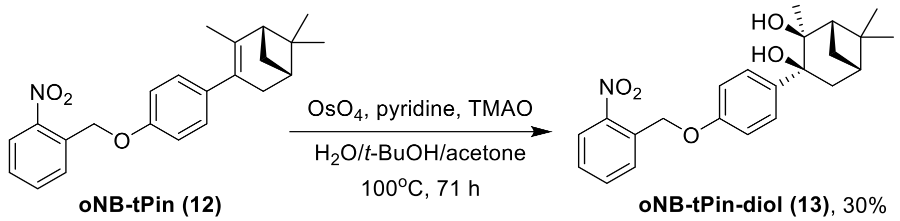

To a mixture of **oNB-tPin (12)** (20 mg, 0.055mmol, 1.0 equiv.) and trimethylamine N-oxide (8.0 mg, 0.11 mmol, 2.0 mmol) in tert-butanol (92 µL), acetone (5.7 µL), and water (28 µL) was added pyridine (4.4 µL) and 2.5% Osmium tetroxide in tert-butanol (20 µL) then stirred for 71 hours at 100°C. The mixture was cooled to room temperature then added sodium bisulfite (5.7 mg, 0.055 mmol, 1 equiv.) to workup. The mixture was stirred for further 30 minutes, followed by filtration through a pad of Celite. The filtrate was extracted with CH_2Cl_2 and brine for 3 times. The combined organic layer was dried over Na_2SO_4, filtered, and concentrated under reduced pressure. The residue was purified by flash column chromatography (Hexane/EtOAc = 4/1) to give **oNB-tPin-diol (13)** (6.5 mg, 30%) as a colorless oil. R_f = 0.20 (Hexane/EtOAc = 4/1). ^{1}H NMR (400 MHz, $CDCl_3$) δ 8.17 (d, *J* = 8.2 Hz, 1H), 7.90 (d, *J* = 7.8 Hz, 1H), 7.68 (t, *J* = 8.0, 7.8 Hz, 1H), 7.51 (d, *J* = 8.9 Hz, 2H), 7.48 (dd, *J* = 8.2, 8.0 Hz, 1H), 6.97 (d, *J* = 8.9 Hz, 2H), 5.50 (s, 2H), 3.88 (s, 1H), 2.90 (s, 1H), 2.79 (d, *J* = 14.6 Hz, 1H), 2.27 (d, *J* = 10.2 Hz, 1H), 2.22 – 2.16 (m, 1H), 2.14 – 2.07 (m, 2H), 1.96 (t, *J* = 5.5 Hz, 1H), 1.33 (s, 3H), 1.01 (s, 3H), 0.85 (s, 3H). ^{13}C NMR (100 MHz, $CDCl_3$) δ 157.18, 147.10, 139.34, 134.14, 134.10, 128.73, 128.44, 127.87, 125.12, 114.42, 78.37, 75.22, 67.00, 55.91, 41.24, 40.22, 37.77, 28.67, 28.35, 24.52, 23.94. HRMS (ESI, m/z): $[M+Na]^+$, calculated: 420.1781, found: 420.1734

Synthesis of OTf-kPin (14)

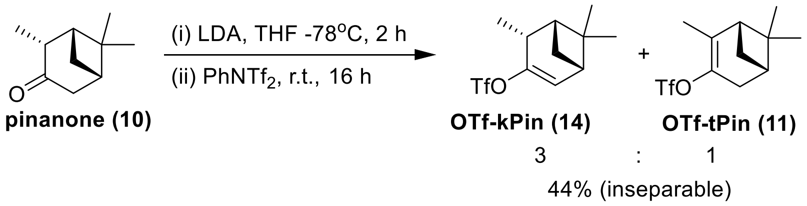

To a dried round-bottom flask was added **pinanone (10)** (0.70 g, 4.6 mmol, 1.0 equiv.) and anhydrous THF (9.2 mL). After cooling the solution to -78°C, 2M lithium diisopropylamide in THF (2.5

mL, 5.1 mmol, 1.1 equiv.) was added dropwise to the solution. The solution was stirred for 2 hours at -78°C, followed by addition of bis(trifluoromethanesulfonyl)aniline (2.5 g, 6.9 mmol, 1.5 equiv.) as powder in a portion then slowly warmed to room temperature. After stirring for 16 hours, the mixture was diluted with EtOAc and washed with brine for 3 times. The organic layer was dried over $MgSO_4$, filtered, and concentrated under reduced pressure. The residue was purified by flash column chromatography with hexane to give a mixture of triflate pinene (**OTf-kPin (14)**: **OTf-tPin (13)** = 3:1, 0.57 g, 44%, with 14% regioisomer) as a white solid. R_f =0.40 (100% Hexane). ^{1}H NMR (400 MHz, $CDCl_3$) δ 6.15 (dd, *J* = 7.2, 1.8 Hz, 1H), 2.94 (q, *J* = 7.4 Hz, 1H), 2.57 – 2.55 (m, 1H), 2.51 (dt, *J* = 9.3, 5.4 Hz, 1H), 2.41 (dt, J = 9.2, 5.4 Hz, 1H), 2.27 – 2.17 (m, 2H), 2.08 (dd, J = 6.7, 5.4 Hz, 1H), 1.77 (t, *J* = 2.2 Hz, 1H), 1.40 (d, *J* = 9.3 Hz, 1H), 1.33 (s, 3H), 1.30 (s, 1H), 1.23 (d, *J* = 7.4 Hz, 3H), 1.07 (s, 3H), 0.92 (s, 1H). ^{13}C NMR (100 MHz, $CDCl_3$) δ 150.79, 140.92, 135.77, 128.40, 125.01, 120.31, 117.12, 113.94, 48.06, 46.54, 41.52, 41.08, 40.67, 39.63, 34.83, 33.31, 31.68, 26.98, 26.02, 23.41, 20.49, 16.85, 14.80. ^{19}F NMR (376 MHz, $CDCl_3$) δ -73.96, -74.77. HRMS (ESI, m/z): $[M+Na]^+$, calculated: 307.0586, found: 307.0614

Synthesis of oNB-kPin (15)

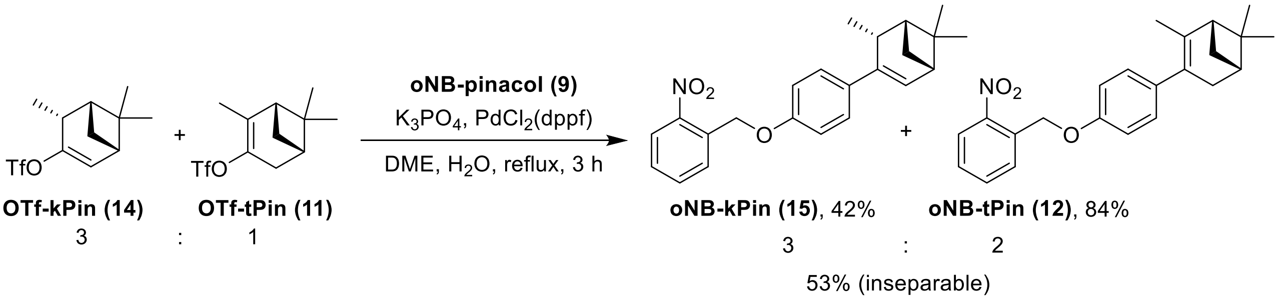

A mixture of triflate pinene (**OTf-kPin (14)**: **OTf-tPin (13)** = 3:1, 200 mg, 0.70 mmol, 1.0 equiv.), **oNB-pinacol (9)** (370 mg, 1.1 mmol, 1.5equiv.), potassium phosphate (300 mg, 1.4 mmol, 2.0 equiv.), and $PdCl_2$(dppf) (50 mg, 0.070 mmol, 0.1 equiv.) were dissolved in dimethoxyethane (7.0 mL) and water (1.4 mL) and stirred for 3 hours at 100°C. Noteworthy, the color of the solution was observed turning to dark during the reaction. After the reaction completed, the solution was diluted with EtOAc and washed with brine for 3 times. The organic layer was dried over $MgSO_4$, filtered, and

concentrated under reduced pressure. The residue was purified by flash column chromatography (Hexane/EtOAc = 19:1) to give a mixture of **oNB-Pin** (**oNB-kPin (15)**: **oNB-tPin (12)** = 3:2, 135 mg, total yield 53%, 42% for **(15)** and 84% for **(12)**) as a yellow solid. R_f = 0.48 (Hexane/EtOAc = 19:1). ^{1}H NMR (400 MHz, $CDCl_3$) δ 8.17 (d, *J* = 8.3 Hz, 2H), 7.94 – 7.90 (m, 2H), 7.68 (dd, *J* = 7.8, 7.6 Hz, 2H), 7.49 (dd, *J* = 8.3, 7.8 Hz, 2H), 7.28 (d, *J* = 8.9 Hz, 2H), 7.18 (d, *J* = 8.8 Hz, 1H), 6.95 (dd, *J* = 8.9, 2.4 Hz, 3H), 6.33 (dd, *J* = 6.3, 1.8 Hz, 1H), 5.50 (s, 4H), 3.16 (qdd, *J* = 7.5, 2.1, 1.9 Hz, 1H), 2.53 – 2.46 (m, 3H), 2.40 (dt, *J* = 8.6, 5.6 Hz, 1H), 2.29 – 2.16 (m, 3H), 2.08 (t, *J* = 5.6 Hz, 1H), 1.70 (t, *J* = 2.1 Hz, 2H), 1.35 (d, *J* = 7.8 Hz, 1H), 1.34 (s, 3H), 1.31 (s, 2H), 1.28 (d, *J* = 8.5 Hz, 1H), 1.06 (d, *J* = 7.4 Hz, 3H), 1.04 (s, 3H), 0.92 (s, 2H). ^{13}C NMR (100 MHz, $CDCl_3$) δ 157.00, 156.48, 147.05, 139.34, 138.59, 135.46, 134.37, 134.32, 134.16, 131.98, 129.57, 128.74, 128.72, 128.37, 127.38, 126.76, 125.10, 114.77, 114.54, 67.03, 48.54, 48.12, 42.64, 41.19, 40.09, 38.73, 38.68, 36.90, 34.65, 31.48, 27.42, 26.43, 24.08, 20.90, 20.88, 17.65. HRMS (ESI, m/z): $[M+H]^+$, calculated: 364.1907, found: 364.1843

Synthesis of oNB-kPin-diol (16)

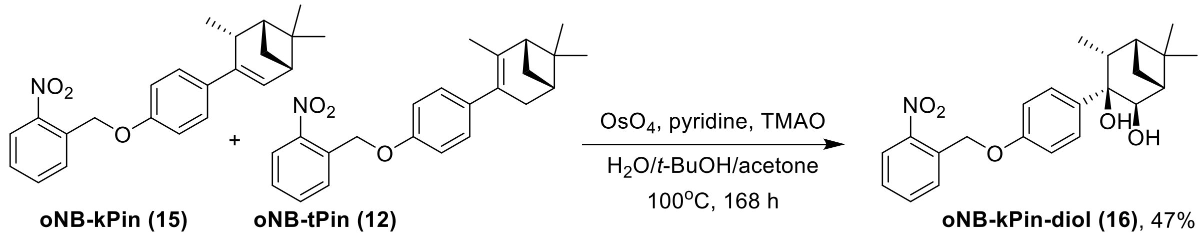

oNB-kPin (15) **oNB-tPin (12)** **oNB-kPin-diol (16)**, 47%

3 : 2

A mixture of **oNB-Pin** (**oNB-kPin (15)**: **oNB-tPin (12)** = 3:2, 27 mg, 0.074 mmol, 1.0 equiv.) in *tert*-butanol (1.2 mL), acetone (600 µL), water (300 µL) was added trimethylamine N-oxide (11 mg, 0.15 mmol, 2.0 equiv.), pyridine (6.0 µL), and 2.5% Osmium tetroxide in *tert*-butanol (47 uL). The resulting solution was stirred for 168 hours at 100°C and the color of the solution was observed turning to be dark. After cooling the reaction to room temperature, sodium sulfite (19 mg, 0.15 mmol, 1 equiv.) was added to quench osmium tetroxide. The mixture was stirred for further 30 minutes, followed by filtration through a pad of Celite. The filtrate was diluted with CH_2Cl_2 then washed with brine for 3 times. The organic layer was dried over Na_2SO_4, filtered, and concentrated under reduced pressure. The residue was purified by flash column chromatography (Hexane/EtOAc = 4/1) to separate **oNB-**

kPin-diol (16) (14 mg, 47%) from the mixture. R_f = 0.25 (Hexane/EtOAc = 4/1). ^{1}H NMR (400 MHz, $CDCl_3$) δ 8.17 (d, *J* = 8.2 Hz, 1H), 7.91 (d, *J* = 7.5 Hz, 1H), 7.69 (dd, *J* = 7.8, 7.5 Hz, 1H), 7.56 (d, *J* = 8.9 Hz, 2H), 7.49 (dd, *J* = 8.2, 7.8 Hz, 1H), 6.98 (d, *J* = 8.9 Hz, 2H), 5.50 (s, 2H), 4.77 (s, 1H), 3.66 (s, 1H), 2.54 (qd, *J* = 7.9, 3.5 Hz, 1H), 2.31 (d, *J* = 9.9 Hz, 1H), 2.15 (m, 1H), 2.07 (t, *J* = 5.2 Hz, 2H), 2.02 (m, 1H), 1.33 (s, 3H), 1.06 (s, 3H), 0.58 (d, *J* = 7.9 Hz, 3H). ^{13}C NMR (100 MHz, $CDCl_3$) δ 157.15, 147.09, 139.31, 134.14, 128.73, 128.43, 128.09, 125.11, 114.53, 76.55, 73.09, 67.01, 48.52, 47.99, 46.16, 42.32, 28.43, 25.24, 23.61, 18.98. HRMS (ESI, m/z): $[M+Na]^+$, calculated: 420.1735, found: 420.1781

Synthesis oNB-kPin-EtPh (17)

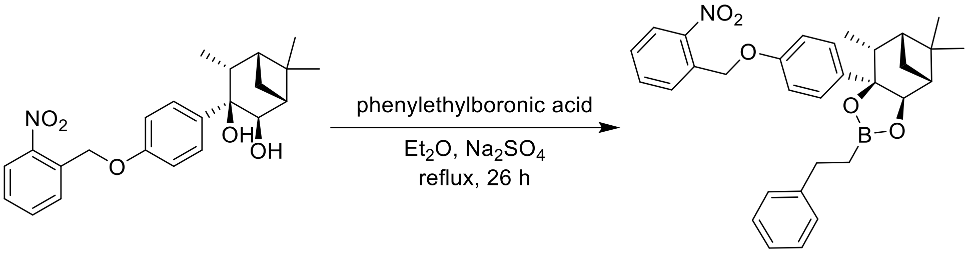

oNB-kPin-diol (16) **oNB-kPin-EtPh (17)**, 39%

To a mixture of **oNB-kPin-diol (16)** (6.0 mg, 0.015 mmol, 1.0 equiv.) and phenylethyl boronic acid (2.2 mg, 0.015 mmol, 1.0 equiv.) in anhydrous Et_2O (1.2 mL) was added Na_2SO_4 (1 g, excess). The mixture was stirred for 26 hours at reflux temperature. After filtration to remove Na_2SO_4, the solid was washed with CH_2Cl_2 for 3 times. The combined filtrate was concentrated under reduced pressure, and purified by flash column chromatography (Hexane/EtOAc = 2/1, R_f =0.73). Next, the product was further purified by reverse phase HPLC to give **oNB-kPin-EtPh (17)** (3.0 mg, 39%) as a colorless oil. HPLC method: column, YMC-Triart C18 (5 µm pore size, 250x10 mm); flow rate, 3.0 mL/min; wavelength,254 nm; mobile phase, water and ACN. Elution gradient was: 0-15 min, 100% water; 15-20min, 100% water–100% ACN; 20-35 min, 100% ACN

^{1}H NMR (400 MHz, $CDCl_3$) δ 8.20 (d, *J* = 8.2 Hz, 1H), 7.94 (d, *J* = 7.8 Hz, 1H), 7.72 (dd, *J* = 7.8, 7.6 Hz, 1H), 7.52 (dd, *J* = 8.2, 7.6 Hz, 1H), 7.50 (d, *J* = 8.9 Hz, 2H), 7.24 – 7.17 (m, 2H), 7.17 – 7.10 (m, 3H), 6.99 (d, *J* = 8.9 Hz, 2H), 5.53 (s, 2H), 5.16 (s, 1H), 2.81 – 2.69 (m, 3H), 2.24 (td, *J* = 5.4, 2.6 Hz, 1H), 2.13 (dt, *J* = 10.5, 5.4 Hz, 1H), 2.03 (m, 1H), 1.47 (d, *J* = 10.5 Hz, 1H), 1.33 (s, 3H), 1.20 (t, *J* = 8.1 Hz, 2H),

1.07 (s, 3H), 0.62 (d, *J* = 7.9 Hz, 3H). ^{13}C NMR (100 MHz, $CDCl_3$) δ 157.17, 147.01, 144.33, 137.39, 134.02, 133.96, 128.60, 128.33, 128.15, 127.94, 127.77, 125.42, 125.00, 114.17, 85.98, 82.03, 66.90, 48.49, 46.71, 45.15, 39.52, 29.97, 27.90, 25.30, 23.33, 19.13. HRMS (ESI, m/z): $[M+K]^+$, calculated: 550.2167, found: 550.2161

Synthesis of Ph-kPin (19)

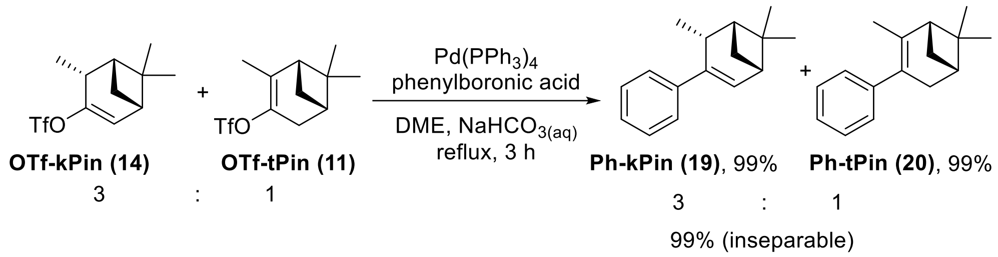

A mixture of triflate pinene (**OTf-kPin (14)**: **OTf-tPin (13)** = 3:1, 50 mg, 0.18 mmol, 1.0 equiv.), phenyl boronic acid (27 mg, 0.22 mmol, 1.2 equiv.), $NaHCO_{3(sat.)}$ (1.8 mL), and $Pd(PPh_3)_4$ (20 mg, 0.018 mmol, 0.1 equiv.) were dissolved in dimethoxyethane (1.8 mL) and stirred for 3 hours at 100°C. Noteworthy, the color of the mixture was observed turning to dark during the reaction. After the reaction completed, the solution was diluted with EtOAc and washed with brine for 3 times. The organic layer was dried over $MgSO_4$, filtered, and concentrated under reduced pressure. The residue was purified by flash column chromatography with hexane to give a mixture of **Ph-Pin** (**Ph-kPin (19)**: **Ph-tPin (20)** = 3:1, 37 mg, yield 99%) as a color less oil. R_f = 0.70 (100% Hexane). ^{1}H NMR (400 MHz, $CDCl_3$) δ 7.36 – 7.30 (m, 5H), 7.26 – 7.21 (m, 2H), 6.41 (d, *J* = 6.4 Hz, 1H), 3.22 (q, *J* = 7.6 Hz, 1H), 2.54 – 2.49 (m, 2H), 2.41 (dt, *J* = 8.2, 5.8 Hz, 1H), 2.31 – 2.21 (m, 2H), 2.1 (t, *J* = 5.6 Hz, 1H), 1.71 (s, 1H), 1.38 (s, 1H), 1.35 (s, 3H), 1.32 (s, 1H), 1.07 (d, *J* = 7.6 Hz, 3H), 1.06 (s, 3H), 0.95 (s, 1H). ^{13}C NMR (100 MHz, $CDCl_3$) δ 142.14, 140.71, 139.68, 139.34, 133.11, 128.45, 128.31, 128.08, 127.52, 126.51, 126.23, 126.06, 48.53, 48.10, 42.68, 41.19, 40.05, 38.70, 38.60, 36.84, 34.62, 31.45, 27.43, 26.45, 24.07, 20.89, 20.85, 17.58.

Synthesis of Ph-kPin-diol (21)

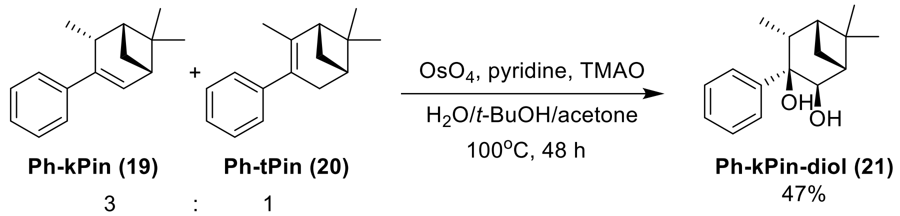

A mixture of **Ph-Pin** (**Ph-kPin (19)**: **Ph-tPin (20)** = 3:1, 20 mg, 0.094 mmol, 1.0 equiv.) in *tert*-butanol (1.0 mL), water (250 µL) was added trimethylamine N-oxide (7.0 mg, 0.094 mmol, 1 equiv.), pyridine (2.0 µL), and 2.5% Osmium tetroxide in *tert*-butanol (30 µL). The mixture was stirred for 48 hours at 100°C and the color of the mixture was observed turning to be dark. After cooling the mixture to room temperature, sodium sulfite (13 mg, 0.094 mmol, 1 equiv.) was added to quench osmium tetroxide. The mixture was stirred for further 30 minutes, followed by filtration through a pad of Celite. The filtrate was diluted with CH_2Cl_2 then washed with brine for 3 times. The organic layer was dried over Na_2SO_4, filtered, and concentrated under reduced pressure. The residue was purified by flash column chromatography (Hexane/EtOAc = 4/1) to give **Ph-kPin-diol (21)** (11 mg, 47%) as a colorless oil. R_f = 0.30 (Hexane/EtOAc = 4/1). x^{1}H NMR (400 MHz, $CDCl_3$) δ 7.63 (d, *J* = 7.4 Hz, 2H), 7.37 (t, *J* = 7.4 Hz, 2H), 7.28– 7.24 (m, 1H), 4.83 (d, *J* = 5.2 Hz, 1H), 3.72 (s, 1H), 2.56 (qd, *J* = 8.2, 3.5 Hz, 1H), 2.34 (d, *J* = 9.8 Hz, 1H), 2.19 – 2.14 (m, 1H), 2.09 (td, *J* = 5.8, 1.6 Hz, 1H), 2.05 – 2.00 (m, 2H), 1.34 (s, 3H), 1.09 (s, 3H), 0.58 (d, *J* = 8.2 Hz, 3H). ^{13}C NMR (100 MHz, $CDCl_3$) δ 146.16, 128.23, 127.04, 126.77, 73.02, 48.50, 47.95, 46.03, 42.35, 28.45, 25.28, 23.59, 18.89. HRMS (ESI, m/z): $[M+Na]^+$, calculated: 269.1512, found: 269.1414

Synthesis of Np-kPin (22)

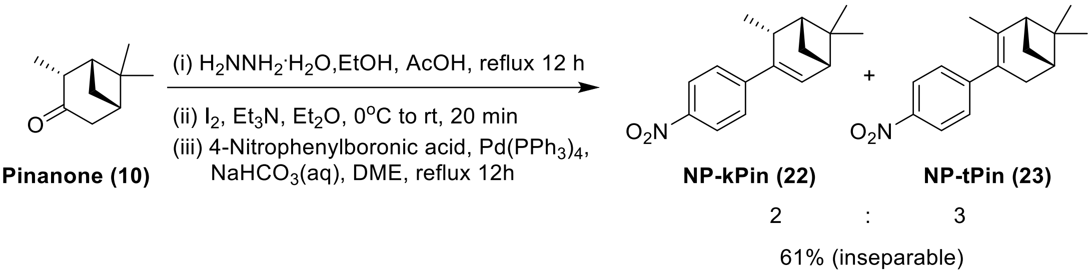

To a solution of **Pinanone (10)** (347.1 mg, 2.3 mmol, 1.0 equiv.) in EtOH (8.0 mL, 0.3M) was added hydrazine monohydrate (0.44 mL, 9.1 mmol, 4.0 equiv.) and acetic acid (0.13 mL, 2.3 mmol,

1.0 equiv.). After stirring for 12 hours at 70°C, the mixture was cooled to room temperature. The mixture was concentrated under reduced pressure to remove EtOH then briefly purified by extracting with CH_2Cl_2 and H_2O to give the hydrazone crude product as a colorless oil. The crude product was dissolved with anhydrous Et_2O (11.5 mL, 0.2M) then cooled to 0°C. To the solution was added iodine as powder in a portion (3.15g, 6.9 mmol, 3.0 equiv.) and Et_3N (2.89 mL, 20.7 mmol, 9.0 equiv.). After warming to room temperature, the mixture was stirring for 20 minutes, followed by working up by $Na_2S_2O_{3(sat.)}$ (10 mL). The mixture was further extracted with Et_2O and $Na_2S_2O_{3(sat.)}$ to give the iodide crude product as an amorphous brown solid. The crude product was suspended in dimethoxyethane (6 mL) and $NaHCO_{3(sat.)}$ (6 mL), and 4-nitrophenylboronic acid (576 mg, 3.5 mmol, 1.5 equiv.) and $Pd(PPh_3)_4$ (133 mg, 0.12 mmol, 0.05 equiv.) were added to the mixture. The mixture was stirred for 2 hours at 100°C. After the reaction completed, the mixture was extracted with EtOAc and H_2O. The combined organic layer was dried over $MgSO_4$, filtered, concentrated under reduced pressure, and purified by flash column chromatography (Hexane/CH_2Cl_2 = 9/1) to give a mixture of **NP-Pin** (**NP-kPin (22)**: **NP-tPin (23)** = 2:3, 355 mg, 61%, with 36% regioisomer) as a yellow solid. R_f =0.30 (Hexane/CH_2Cl_2 = 19/1). ^1H NMR (400 MHz, $CDCl_3$) δ 8.18 (dd, *J* = 8.9, 8.8 Hz, 3H), 7.47 (d, *J* = 8.9 Hz, 1H), 7.38 (d, *J* = 8.8 Hz, 2H), 6.63 (d, *J* = 6.4 Hz, 1H), 3.06 (q, *J* = 7.0 Hz, 1H), 2.55 (s, 2H), 2.49 – 2.37 (m, 2H), 2.32 (dt, *J* = 9.0, 5.7 Hz, 1H), 2.27 – 2.23 (m, 1H), 2.16 (t, *J* = 5.6 Hz, 1H), 2.08 (td, *J* = 5.8, 2.7 Hz, 1H), 1.74 (s, 3H), 1.36 (s, 2H), 1.33 (s, 3H), 1.32 – 1.27 (m, 2H), 0.99 (d, *J* = 7.0 Hz, 2H), 0.92 (s, 3H), 0.90 (s, 2H). ^{13}C NMR (100 MHz, $CDCl_3$) δ 149.21, 143.54, 129.13, 126.30, 123.89, 123.51, 48.45, 41.03, 38.74, 36.34, 31.19, 26.33, 21.09, 20.89. HRMS (ESI, m/z): $[M+H]^+$, calculated: 258.1489, found: 258.1517

Synthesis of Np-kPin-diol (24)

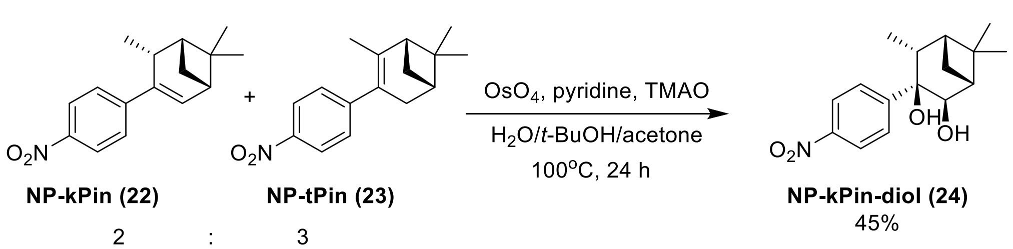

A mixture of **NP-Pin** (**NP-kPin (22)**: **NP-tPin (23)** = 2:3, 40 mg, 0.16 mmol, 1.0 equiv.) in *tert*-

butanol (750 μL), acetone (250 μL), water (25 μL) was added trimethylamine N-oxide (12.3 mg, 0.17 mmol, 2.0 mmol), pyridine (12.3 μL, 0.17, 1.0 equiv.), and 2.5% Osmium tetroxide in tert-butanol (100 μL). The mixture was stirred for 24 hours at 100°C. After cooling the mixture to room temperature, sodium sulfite was added to quench osmium tetroxide. The mixture was stirred for further 30 minutes, followed by filtration through a pad of Celite. The filtrate was diluted with CH_2Cl_2 then washed with H_2O for 3 times. The organic layer was dried over Na_2SO_4, filtered, and concentrated under reduced pressure. The residue was purified by flash column chromatography (Hexane/EtOAc = 6/1) to give **NP-kPin-diol (24)** (21mg, 45%) as a white amorphous solid. Rf = 0.25 (Hexane/EtOAc = 6/1). ^{1}H NMR (400 MHz, $CDCl_3$) δ 8.20 (d, *J* = 9.0 Hz, 2H), 7.86 (d, *J* = 9.0 Hz, 2H), 4.36 (d, *J* = 5.2 Hz, 1H), 3.69 (s, 1H), 2.45 (d, *J* = 10.1 Hz, 1H), 2.39 (q, *J* = 7.0 Hz, 1H), 2.18 – 2.09 (m, 2H), 2.06 (td, *J* = 5.5, 1.4 Hz, 1H), 1.81 (t, *J* = 5.7 Hz, 1H), 1.36 (s, 3H), 1.09 (s, 3H), 0.95 (d, *J* = 7.0 Hz, 3H). ^{13}C NMR (100 MHz, $CDCl_3$) δ 155.70, 147.05, 127.02, 123.47, 76.02, 74.93, 48.83, 48.20, 41.68, 39.22, 27.53, 22.81, 20.71, 13.62. HRMS (ESI, m/z): $[M+NH_4]^+$, calculated: 309.1809, found: 309.1725

Synthesis of pNB-Br (25)

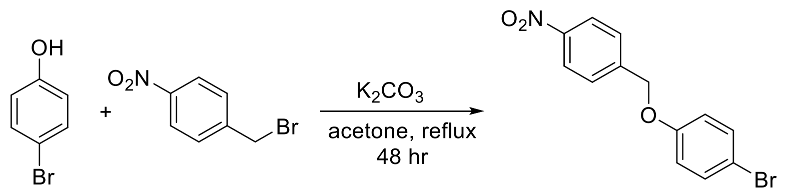

pNB-Br (25), 70 %

To a solution of 4-nitrobenzyl bromide (832.5 mg, 3.9 mmol, 1.0 equiv.) and 4-bromophenol (2.0 g, 11.6 mmol, 3.0 equiv.) in acetone (20 mL) was added potassium carbonate (1.07 g, 7.6 mmol, 2.0 equiv.). The mixture was stirred for 48 hours at 60°C. After the reaction completed, the mixture was diluted with CH_2Cl_2 and washed with 2N $NaOH_{(aq)}$ for 3 times. The organic layer was dried over $MgSO_4$, filtered, and concentrated under reduced pressure. The residue was purified by flash column chromatography (Hexane/EtOAc = 19/1 to 9/1) to give **pNB-Br (25)** (831.7 mg, 70%) as a pale-yellow solid. R_f = 0.55 (Hexane/EtOAc = 4/1). ^{1}H NMR (400 MHz, $CDCl_3$) δ 8.25 (d, *J* = 8.8 Hz, 2H), 7.59 (d, *J* = 8.8 Hz, 2H), 7.40 (d, *J* = 9.0 Hz, 2H), 6.85 (d, *J* = 9.0 Hz, 2H), 5.15 (s, 2H) ^{13}C NMR (100 MHz, $CDCl_3$) δ 157.39, 147.87, 144.12, 132.67, 127.75, 124.04, 116.79, 114.02, 69.06. $[M+H]^+$, calculated: 307.9917,

found: 308.0012

Synthesis of pNB-Pinacol (26)

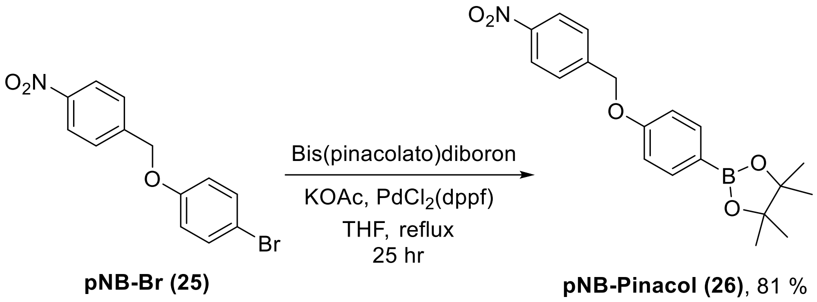

To a mixtrure of **pNB-Br (25)** (200 mg, 0.65 mmol, 1.0 equiv.), bis(pinacolato)diboron (445 mg, 1.8 mmol, 2.7 equiv.), potassium acetate (127 mg, 1.3mmol, 2.0 equiv.), and $PdCl_2$(dppf) (24 mg, 0.032 mmol, 0.05 equiv.) was added THF (5.0 mL). The mixture was stirred for 25 hours at reflux temperature. After the reaction completed, the mixture was diluted with CH_2Cl_2 and washed with H_2O for 3 times. The organic layer was dried over $MgSO_4$, filtered, and concentrated under reduced pressure. The residue was purified by flash column chromatography (Hexane/EtOAc = 19/1) to give **pNB-Pinacol (26)** (187 mg, 81%) as a white solid. R_f =0.20 (Hexane/EtOAc = 19/1). ^{1}H NMR (400 MHz, $CDCl_3$) δ 8.23 (d, *J* = 8.6 Hz, 2H), 7.77 (d, *J* = 8.6 Hz, 2H), 7.60 (d, *J* = 8.6 Hz, 2H), 6.96 (d, *J* = 8.6 Hz, 2H), 5.20 (s, 2H), 1.33 (s, 12H). ^{13}C NMR (100 MHz, $CDCl_3$) δ 160.75, 147.75,144.48, 136.82, 127.72, 123.96, 114.27, 83.83, 68.53, 25.00. HRMS (ESI, m/z): $[M+H]^+$, calculated: 356.1667, found: 356.1644

Synthesis of pNB-kPin (27)

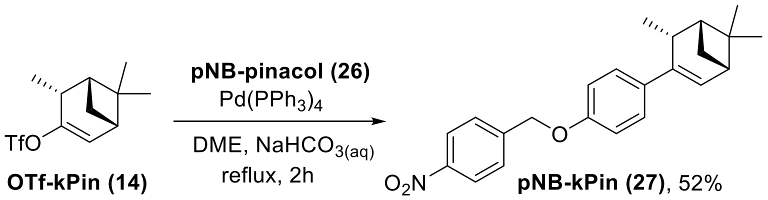

A mixture of **OTf-kPin (14)** (200 mg, 0.70 mmol, 1.0 equiv.), **pNB-pinacol (26)** (370 mg, 1.1 mmol, 1.5 equiv.), and $Pd(PPh_3)_4$ (50 mg, 0.043 mmol, 0.06 equiv.) were dissolved in dimethoxyethane (3.5 mL) and $NaHCO_{3(sat.)}$ (3.5 mL) and stirred for 2 hours at 100°C. After the reaction completed, the solution was diluted with CH_2Cl_2 and washed with H_2O for 3 times. The organic layer was dried over $MgSO_4$, filtered, and concentrated under reduced pressure. The residue was purified by flash column

chromatography (Hexane/EtOAc = 19:1) to give **pNB-kPin (27)** (132 mg, yield 52%) as a pale-yellow solid. R_f = 0.38 (Hexane/EtOAc = 19:1). ^{1}H NMR (400 MHz, $CDCl_3$) δ 8.25 (d, *J* = 8.8 Hz, 2H), 7.62 (d, *J* = 8.8 Hz, 2H), 7.28 (d, *J* = 8.8 Hz, 3H), 6.92 (d, *J* = 8.8 Hz, 2H), 6.33 (d, *J* = 6.3 Hz, 1H), 5.18 (s, 2H), 3.16 (q, *J* = 7.5 Hz, 1H), 2.52 – 2.47 (m, 1H), 2.29 – 2.21 (m, 2H), 1.35 (s, 1H), 1.34 (s, 3H), 1.05 (d, *J* = 7.5 Hz, 3H), 1.03 (s, 3H). ^{13}C NMR (100 MHz, $CDCl_3$) δ 156.99, 147.70, 144.88, 138.55, 134.27, 132.09, 127.73, 127.40, 123.97, 114.68, 68.88, 48.54, 42.64, 40.11, 38.67, 34.65, 27.41, 24.08, 17.64. HRMS (ESI, m/z): $[M+H]^+$, calculated: 364.1907, found: 364.1858

Synthesis of pNB-kPin-diol (28)

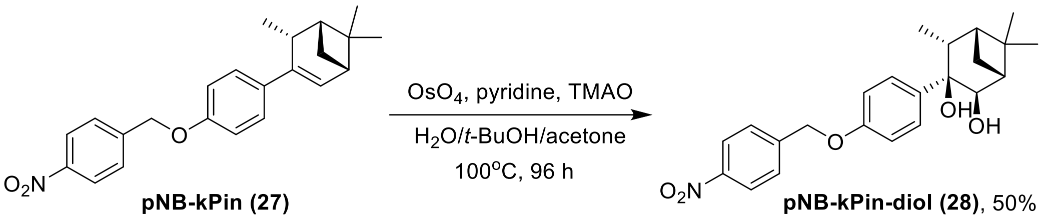

To a solution of **pNB-kPin (27)** (18.4 mg, 0.051 mmol, 1.0 equiv.) in *tert*-butanol (500 µL), acetone (200 µL), water (500 µL) was added trimethylamine N-oxide (10 mg, 0.14 mmol, 2.0 mmol), pyridine (5.0 µL, 0.074, 1.5 equiv.), and 2.5% Osmium tetroxide in *tert*-butanol (25 uL). The mixture was stirred for 96 hours at 100°C. After cooling the mixture to room temperature, sodium sulfite was added to quench osmium tetroxide. The mixture was stirred for further 30 minutes, followed by filtration through a pad of Celite. The filtrate was diluted with CH_2Cl_2 then washed with H_2O for 3 times. The organic layer was dried over Na_2SO_4, filtered, and concentrated under reduced pressure. The residue was purified by flash column chromatography (Hexane/EtOAc = 4/1) to give **pNB-kPin-diol (28)** (10 mg, 50%) as a white amorphous solid. R_f = 0.10 (Hexane/EtOAc = 4/1). ^{1}H NMR (400 MHz, $CDCl_3$) δ 8.25 (d, *J* = 8.4 Hz, 2H), 7.61 (d, *J* = 8.4 Hz, 2H), 7.56 (d, *J* = 8.7 Hz, 2H), 6.96 (d, *J* = 8.7 Hz, 2H), 5.18 (s, 2H), 4.77 (d, *J* = 5.6 Hz, 1H), 3.64 (s, 1H), 2.54 (qd, *J* = 7.9, 3.5 Hz, 1H), 2.31 (d, *J* = 9.8 Hz, 1H), 2.18 – 2.13 (m, 1H), 2.08 (t, *J* = 5.4 Hz, 1H), 2.04 – 1.97 (m, 2H), 1.33 (s, 3H), 1.07 (s, 3H), 0.59 (d, *J* = 7.9 Hz, 3H). ^{13}C NMR (100 MHz, $CDCl_3$) δ 157.13, 147.74, 144.73, 139.38, 128.12, 127.74, 123.99, 114.40, 76.51, 73.11, 68.83, 48.49, 47.98, 46.12, 42.32, 28.41, 25.23, 23.61, 18.97. HRMS (ESI, m/z): $[M+Na]^+$, calculated: 420.1781, found: 420.1726

3. Hydrolysis, Assembly and Fluorescence Detection Assays

General methods

Preparation of the stock solutions and buffers

Stock solutions were prepared by dissolving 25 mM pinanediol cages or boronic ester in DMSO and stored at -20°C until use. Before HPLC tests, the compound solution was freshly prepared by diluting the stock solution. All pH buffer solutions were prepared from a 0.1 M phosphate buffer solution and adjusted with 1 M potassium hydroxide solution or diluted phosphoric acid solution.

Methods for preparation of the photouncaged pinanediol cage or boronic ester

The stock solutions of **Phe-kPin-EtPh (17a)** and **Phe-kPin-diol (16a)** (25 mM each) were freshly prepared by in situ irradiation of **oNB-kPin-EtPh (17)** and **oNB-kPin-diol (16)** (25 mM each) with an LED at a wavelength of 365 nm for 1.5 minutes.

Methods for preparation of the NTR-reduced pinanediol cages

Nitroreductase NfsB (NTR) from *Escherichia coli* was prepared as previously reported.[1,2]

Pinanediol cages, **NP-kPin-diol (24)** or **pNB-kPin-diol (28)** (25 μM), nicotinamide adenine dinucleotide (NADH) (250 μM), and NTR (25 μM) were prepared in a solution of 2% DMSO/ PBS (500 μL). The resulting solution was incubated at 37°C for 4 hours.

Hydrolysis and assembly assays

Methods for hydrolysis assays to evaluate hydrolytic stability of boronic esters

Boronic ester (250 µM) was prepared in a mixture of 50% dimethyl sulfoxide (DMSO)/pH 7.4 phosphate buffered saline (PBS) buffer (200 µL). The solutions were incubated at 37°C for certain incubation times. After incubation, the sample was monitored by HPLC at a wavelength of 210 nm. The relative abundance of remaining boronic esters was quantified by comparing the area under the curve (AUC) of the standard solution (200 µL, 250 µM).

Methods for assembly assays to evaluate hydrolytic stability of boronic esters

A: Pinanediol cages (250 μM) were prepared in a solution of 50% dimethyl sulfoxide (DMSO)/pH 7.4 phosphate buffered saline (PBS) buffer (200 μL) with boronic acid **PhEtBA** (250 μM). (Pinanediol cages: **tPin-diol (1)** or mixture of **k/tPin-diol (6/1)** (3:1 molar ratio))

B: Pinanediol cages (25 μM) were prepared in a solution of 2% DMSO/ PBS (500 μL) with boronic acid **PhEtBA** (25 μM). (Pinanediol cages: **k/tPin-diol (6/1)** (3:1 molar ratio), **Ph-kPin-diol (21)**, **NP-kPin-diol (24)**, **oNB-kPin-diol (16)**, **pNB-kPin-diol (28)**, or **Phe-kPin-diol (16a)**)

The resulting solution was incubated at 37°C for 4 hours. After incubation, the sample was monitored by HPLC at a wavelength of 210 nm. The yield of boronic ester formation was estimated by comparing the area under the curve (AUC) of the remaining boronic acid.

Fluorescence detection with McCDBA and pinanediol cages

Methods for fluorescence detection with McCDBA and pinanediol cages

Stock solutions were prepared by dissolving 10 mM **McCDBA** and pinanediol cages in DMSO and stored at -20°C until use. Before spectroscopic tests, the probe solution was freshly prepared by diluting the stock solution to record the fluorescence spectra. The glucose detection assays were carried out in pH 7.4 PBS buffer, in pH buffer solutions prepared from a 0.1 M phosphate buffer solution, or in DMEM (without glucose).

Methods for preparation of the NTR-reduced pinanediol cages with McCDBA

McCDBA, pinanediol cages, **NP-kPin-diol (24)** or **pNB-kPin-diol (28)**, nicotinamide adenine dinucleotide (NADH) (250 μM), and NTR (25 μM) were prepared in a solution of 1% DMSO/ PBS. The resulting solution was incubated at 37°C for 2 hours and then fluorescence intensities were recorded using a microplate reader.

4. Determination of the Binding Constants

Determination of the binding constants (K_a) between *D*-glucose and McCDBA

The binding constant (K_a) between *D*-glucose and **McCDBA** was determined by Bensi—Hildebrand plot. A solution **McCDBA** (2.5 μM) was prepared in glucose free DMEM. Then glucose was added to give solutions with a range of concentrations (0 to 62.5 μM). After 2-hours incubation at 37 ^{o}C, the fluorescence intensities were measured (λex: 360 nm, λem: 450 nm) by a microplate reader. The relationship between fluorescence intensity changes and the binding constant can be expressed by Eq. (1)

$$\Delta I_F = \frac{\Delta kp_0 K_a[\mathbf{Glu}][\mathbf{McCDBA}]_0}{1 + K_a[\mathbf{Glu}]} \qquad \text{Eq.1}$$

Where $\mathbf{I_F}$ is fluorescent intensity, Δkp_0 is a constant derived from intrinsic fluorescence and the laser power, **Glu** is glucose, [**McCDBA**]$_0$ is the total concentration of **McCDBA**, and K_a is the binding constant between glucose and **McCDBA**.

The double reciprocal of Eq. (1) yields the Bensi-Hildebrand equation (Eq. (2). The binding constant (K_a) between *D*-glucose and **McCDBA** is the quotient of the intercept and the slope in the plot 1/[**Glu**] vs. 1/Δ$\mathbf{I_F}$. (**Figure S13**)

$$\frac{1}{\Delta I_F} = (\Delta kp_0 [\mathbf{McCDBA}]_0 K_a)^{-1} \frac{1}{[\mathbf{Glu}]} + (\Delta kp_0 [\mathbf{McCDBA}]_0)^{-1} \qquad \text{Eq.2}$$

Determination of the apparent binding constants (K_{app}) between pinanediol cages and McCDBA

The apparent binding constants (K_{app}) between pinanediol cages and **McCDBA** were determined by glucose-competitive titrations. The titration of pinanediol cages perturbed the equilibrium of *D*-glucose and **McCDBA** and thereby resulted in changes of the fluorescence intensities of the solutions. A solution of **McCDBA** (2.5 μM) was prepared in DMEM with 0.1M *D*-glucose. Then, the four pinanediol cages, **oNB-kPin-diol (16)**, **Phe-kPin-diol (16a)**, **pNB-kPin-diol (28)**, and **NP-kPin-diol (24)** was added to give solutions with a range of concentrations (0 to 25 μM), respectively. After a 2-hour incubation at 37°C, the fluorescence intensities were measured (λex: 360 nm, λem: 450 nm) using a

microplate reader. The relationships between *D*-glucose, pinanediol cages, and **McCDBA** was simplified and described by Eq. (3) to (5). In addition, the relationships between the concentrations of the species in these equations can be described by Eq. (6) to (8).

$$\textbf{McCDBA} + \textbf{Glu} \rightleftharpoons \textbf{Glu-McCDBA} \qquad \text{Eq.3}$$

$$\textbf{McCDBA} + \textbf{diol} \rightleftharpoons \textbf{diol-McCDBA} \qquad \text{Eq.4}$$

$$\textbf{Glu-McCDBA} + \textbf{diol} \rightleftharpoons \textbf{diol-McCDBA} + \textbf{Glu} \qquad \text{Eq.5}$$

where **Glu** is *D*-glucose, **diol** is pinanediol cage, **Glu-McCDBA** is the boronic ester complex of *D*-glucose and **McCDBA**, **diol-McCDBA** is the boronic ester complex of a pinanediol cage and **McCDBA**.

$$[\textbf{McCDBA}]_0 = [\textbf{McCDBA}] + [\textbf{Glu-McCDBA}] + [\textbf{diol-McCDBA}] \qquad \text{Eq.6}$$

$$[\textbf{Glu}]_0 = [\textbf{Glu}] + [\textbf{Glu-McCDBA}] \qquad \text{Eq.7}$$

$$[\textbf{diol}]_0 = [\textbf{diol}] + [\textbf{diol-McCDBA}] \qquad \text{Eq.8}$$

Where $[\textbf{McCDBA}]_0$ is the total concentration of **McCDBA**, $[\textbf{diol}]_0$ is the total concentration of pinanediol cage, and $[\textbf{Glu}]_0$ is the total concentration of *D*-glucose.

In the presence of a significant excess of *D*-glucose (0.1M), we assumed nearly all **McCDBA** forms **Glu-McCDBA** with *D*-glucose. Therefore, when the added pinanediol cages reaches EC_{50} in the glucose-competitive titrations, the concentration of fluorescent **Glu-McCDBA** should be approximately equal to $[\textbf{McCDBA}]_0/2$. In this way, by definition, the binding constant between *D*-glucose and **McCDBA**, *K*a, can be written as Eq. (9). After rearranging, [**McCDBA**] can be described as Eq. (10), and [**diol-McCDBA**] can be described as Eq. (11).

$$K_a = \frac{[\textbf{Glu-McCDBA}]}{[\textbf{McCDBA}][\textbf{Glu}]} = \frac{[\textbf{McCDBA}]_0/2}{[\textbf{McCDBA}]([\textbf{Glu}]_0 - [\textbf{McCDBA}]_0/2)} \qquad \text{Eq.9}$$

$$[\textbf{McCDBA}] = \frac{[\textbf{McCDBA}]_0/2}{K_a\ ([\textbf{Glu}]_0 - [\textbf{McCDBA}]_0/2)} \qquad \text{Eq.10}$$

$$[\textbf{diol-McCDBA}] = [\textbf{McCDBA}]_0/2 - [\textbf{McCDBA}] \qquad \text{Eq.11}$$

Where K_a is the binding constant between glucose and **McCDBA**.

Take Eq. (10) and (11) together, the apparent binding constants (K_{app}) between pinanediol cages and **McCDBA** can be expressed as a function of the concentrations of substances and K_a. The K_{app} values for various pinanediol cages and **McCDBA** can be determined through their respective EC_{50} in glucose-competitive titrations.

$$K_{\mathrm{app}} = \frac{[\textbf{diol-McCDBA}]}{[\textbf{McCDBA}][\textbf{diol}]} = \frac{[\textbf{McCDBA}]_0/2 - [\textbf{McCDBA}]}{[\textbf{McCDBA}]([\textbf{diol}]_0 - [\textbf{McCDBA}]_0/2 + [\textbf{McCDBA}])} \qquad \text{Eq.12}$$

Where K_{app} is the apparent binding constants between pinanediol cages and **McCDBA**, **[McCDBA]** is expressed by Eq. (10), and [**diol**]$_0$ is the EC_{50} concentration of titrations of each pinanediol cage.

5. Cell Experiments

Cell line and cell culture

HeLa, HEK293T, and HCT116 cells were purchased from BCRC. HeLa and HEK293T cells were cultured in DMEM supplemented with 10% FBS and 1% penicillin-streptomycin at 37°C with 5% CO_2. HCT116 cells were cultured in McCoy's 5A medium supplemented with 10% FBS, 100 units/mL penicillin, and 100 μg/mL streptomycin at 37°C with 5% CO_2.

HeLa, HEK293T, or HCT116 cells were seeded into a poly-*L*-lysine-coated 96-well plate at a density of 10,000 cells per well in 100 μL DMEM or McCoy's 5A medium with 10% FBS, 100 units/mL penicillin, and 100 μg/mL streptomycin. The 96-well plates were incubated at 37°C with 5% CO_2 for 24 hours before conducting the following experiments.

For the cocultured cell experiments, HEK293T and HCT116 cells were seeded into the same wells of a poly-*L*-lysine-coated 96-well plate at a density of 5,000 cells per well for each cell type (total 10,000 cells per well) in 100 μL RPMI 1640 medium with 10% FBS, 100 units/mL penicillin, and 100 μg/mL streptomycin.

Cytotoxicity assay

Alamar Blue assays were conducted to determine the cytotoxicity to HeLa, HEK293T, and HCT116 cells. After a 24-hour incubation in 96-well plates, the adherent state of the cells was observed under a microscope. The medium was then replaced with 100 μL of medium containing 1% DMSO and 50 μM of the corresponding compounds (**McCDBA**, pinanediol cages, or **McCDBA** + pinanediol cages). After 24 hours, the medium was replaced with 100 μL of serum-free medium containing 0.001 mg/mL resazurin sodium and further cultured in the cell incubator. After 4 hours, the fluorescence intensities at excitation/emission 544/590 nm were measured using a microplate reader.

Cell uptake assay

Cell uptake assays were carried out in HeLa cells to determine the optimal incubation time for

the following experiments. After a 24-hour incubation in 96-well plates, the adherent state of the HeLa cells was observed under a microscope. The medium was then replaced with 100 µL of medium containing 1% DMSO and 50 µM **McCDBA**. Cell images were acquired by fluorescence microscopy (ImageXpress Pico Automated Cell Imaging System, DAPI, λ_{ex}: 350−390 nm, λ_{em}: 410−482 nm) at 0, 5, 15, and 30 minutes, respectively. Images were processed using ImageJ software.

Method for fluorescent imaging of cells with the photo-responsive pinanediol cage

Cellular fluorescence detection with the photo-responsive pinanediol cage was carried out in HeLa cells. After a 24-hour incubation in 96-well plates, the adherent state of the HeLa cells was observed under a microscope. The cells were stained with 1 µM calcein AM in 100 µL of medium for 30 minutes. The medium was then replaced with 100 µL of medium containing 1% DMSO and 50 µM of the corresponding compounds (**McCDBA**, **McCDBA** + **oNB-kPin-diol (16)**, **McCDBA + Phe-kPin-diol (16a)**, or DMSO (untreated). For **McCDBA** + pinanediol cages, **McCDBA** was first added to the cells for 15 minutes, followed by the addition of diol cages to the medium for another 15 minutes. After a total of 30 minutes of incubation, the medium was removed, and the cells were washed with PBS three times. Cell images were acquired by fluorescence microscopy (ImageXpress Pico Automated Cell Imaging System, DAPI, λ_{ex}: 350−390 nm, λ_{em}: 410−482 nm; FITC, λ_{ex}: 445−485 nm, λ_{em}: 509−539 nm). Images were processed using ImageJ software. The fluorescence intensities of each cell were shown as scatter plots.

Method for fluorescent imaging of cells with regioselective irradiation

Direct photo-activation of cellular caged **McCDBA** was demonstrated in HeLa cells using the ROI Bleaching function of Zeiss LSM 980 confocal microscope. After a 24-hour incubation in 96-well plates, the adherent state of the HeLa cells was observed under a microscope. The cells were stained with 1 µM calcein AM in 100 µL of medium for 30 minutes. The medium was then replaced with 100 µL of medium containing 1% DMSO and 50 µM of the corresponding compounds (**McCDBA**, **McCDBA** + **oNB-kPin-diol (16)**, or DMSO (untreated)). For **McCDBA** + pinanediol cages, **McCDBA** was first added

to the cells for 15 minutes, followed by the addition of diol cages to the medium for another 15 minutes. After a total of 30 minutes of incubation, the medium was removed, and the cells were washed with PBS three times. Cell images were acquired using fluorescence microscopy (Zeiss LSM 980, DAPI, λ_ex: 405 nm, λ_em: 410−470 nm; FITC, λ_ex: 488 nm, λ_em: 500−550 nm). Six cells in the **McCDBA** + **oNB-kPin-diol (16)** group were selected and irradiated (405 laser: 30 mW nominal power, 14 mW ex fiber; Experimental conditions: 30% intensity, 45 pulses, 0.1 ms per pulse) using the ROI Bleaching function of Zeiss LSM 980 confocal microscope software. The selected cells were further monitored for 15 minutes. Images were processed using ImageJ software. The fluorescence intensities of each cell were presented as scatter plots.

Method for fluorescent imaging of normal and tumor cells with the NTR-responsive pinanediol cage

Cellular fluorescence detection with the NTR-responsive pinanediol cages was carried out in HEK293T, HCT116 cells, or cocultured samples of HEK293T and HCT116 cells. After a 24-hour incubation in 96-well plates, the adherent state of the cells was observed under a microscope. The cells were stained with 1 µM calcein AM in 100 µL of medium for 30 minutes. The medium was then replaced with 100 µL of medium containing 1% DMSO and 50 µM of the corresponding compounds (**McCDBA**, **McCDBA** + **pNB-kPin-diol (28)**, **McCDBA** + **NP-kPin-diol (24)**, or DMSO (untreated)). For **McCDBA** + pinanediol cages, **McCDBA** was first added to the cells for 15 minutes, followed by the addition of diol cages to the medium for another 15 minutes. After a total of 30 minutes of incubation, the 96-well plates were incubated at 37°C with 5% CO_2 under normoxia (20% O_2) or hypoxia (1% O_2) for an additional 8 hours. The medium was then removed, and the cells were washed with PBS three times. Cell images were acquired using fluorescence microscopy (ImageXpress Pico Automated Cell Imaging System, DAPI, λ_ex: 350−390 nm, λ_em: 410−482 nm; FITC, λ_ex: 445−485 nm, λ_em: 509−539 nm). Images were processed using ImageJ software. The fluorescence intensities of each cell were presented as scatter plots.

6. Supporting Schemes and Figures

(a)

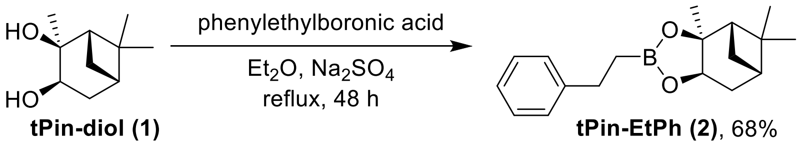

(b)

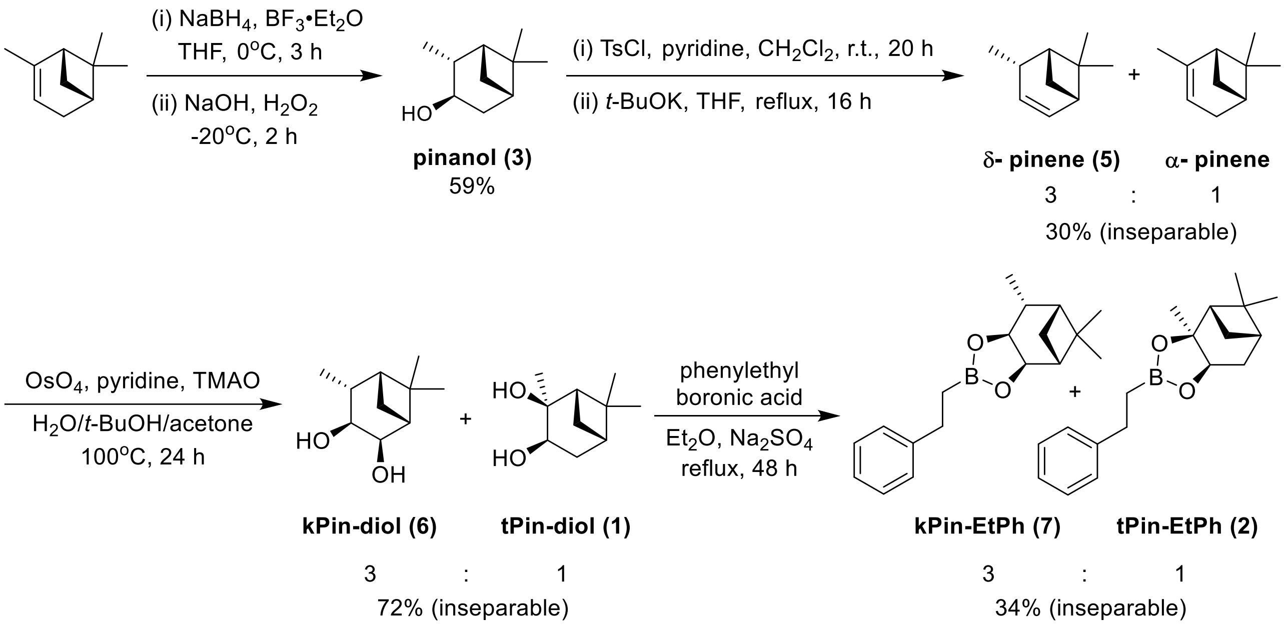

Scheme S1. Synthesis of (a) **tPin-EtPh (2)** and (b) **kPin-EtPh (7)**

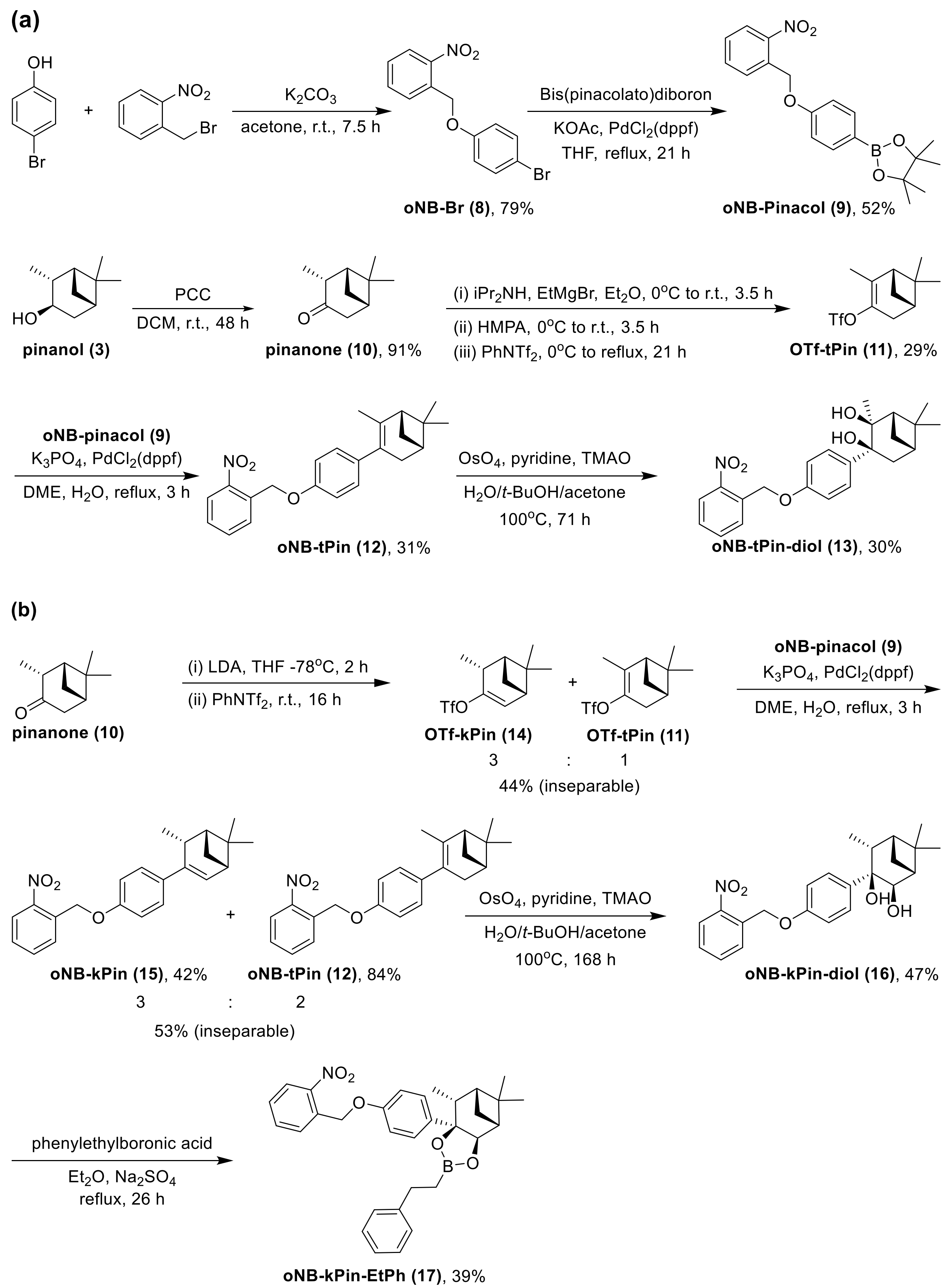

Scheme S2. Synthesis of (a) **oNB-tPin-diol (13)**, (b) **oNB-kPin-diol (16)**, and **oNB-kPin-EtPh (17)**

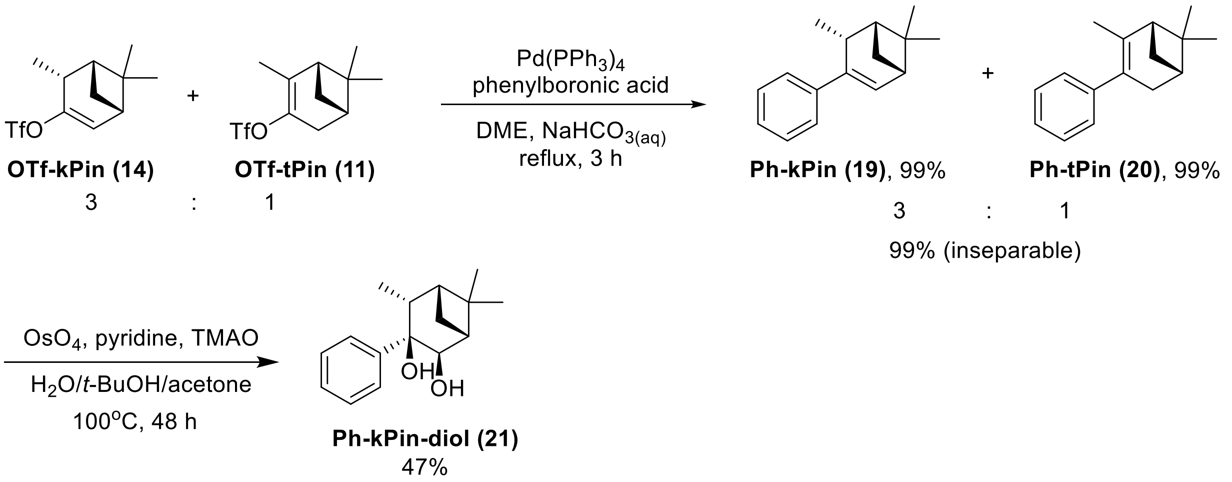

Scheme S3. Synthesis of **Ph-kPin-diol (21)**

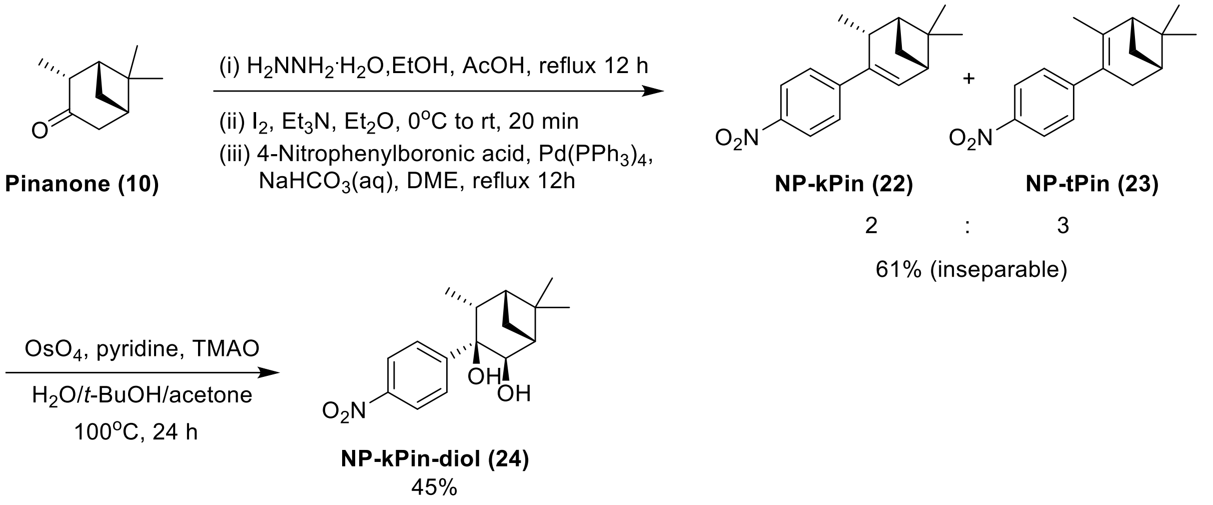

Scheme S4. Synthesis of **Np-kPin-diol (24)**

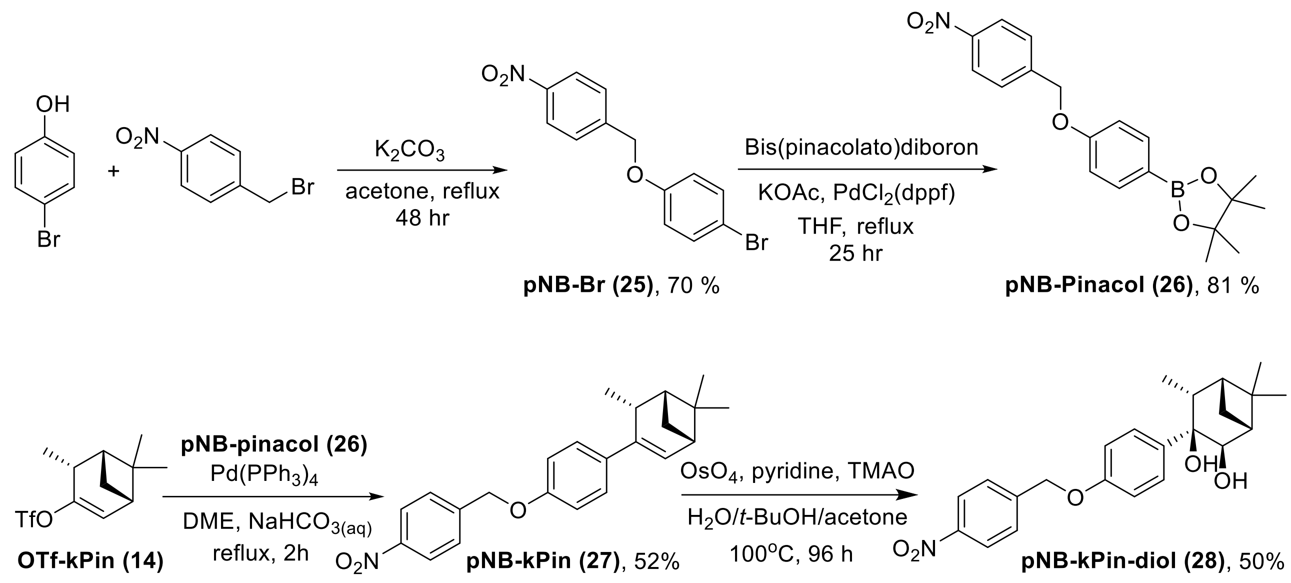

Scheme S5. Synthesis of **pNB-kPin-diol (28)**

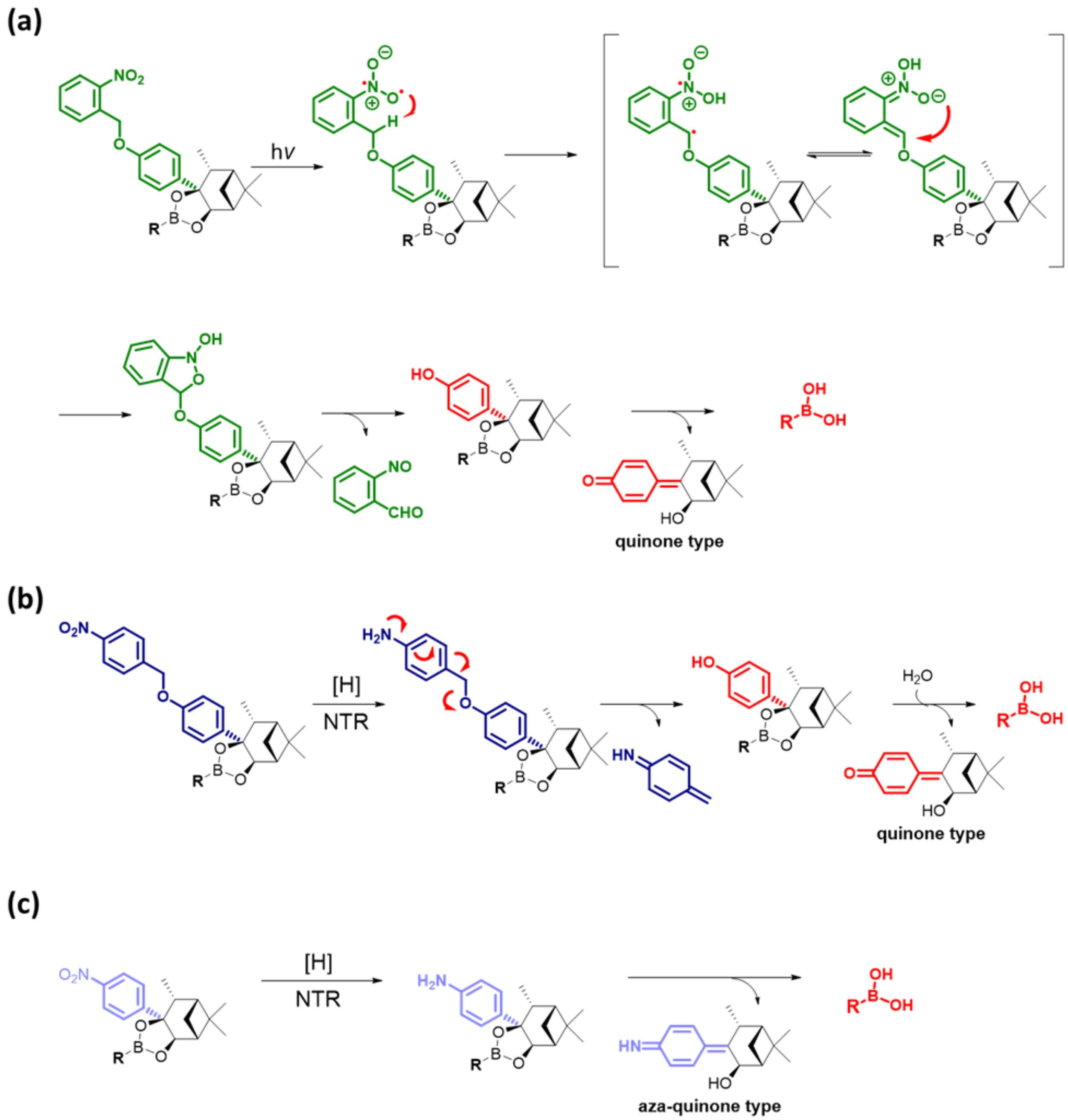

Scheme S6. Proposed releasing mechanism of stimuli-responsive releasing of boronic acids. (a) Scheme to illustrate a boronic acid releasing from a boronic ester caged by **oNB-kPin-diol (16)** upon UV irradiation (b) Scheme to illustrate a boronic acid releasing from a boronic ester caged by **pNB-kPin-diol (28)** and (c) **NP-kPin-diol (24)** upon exposure to NTR.

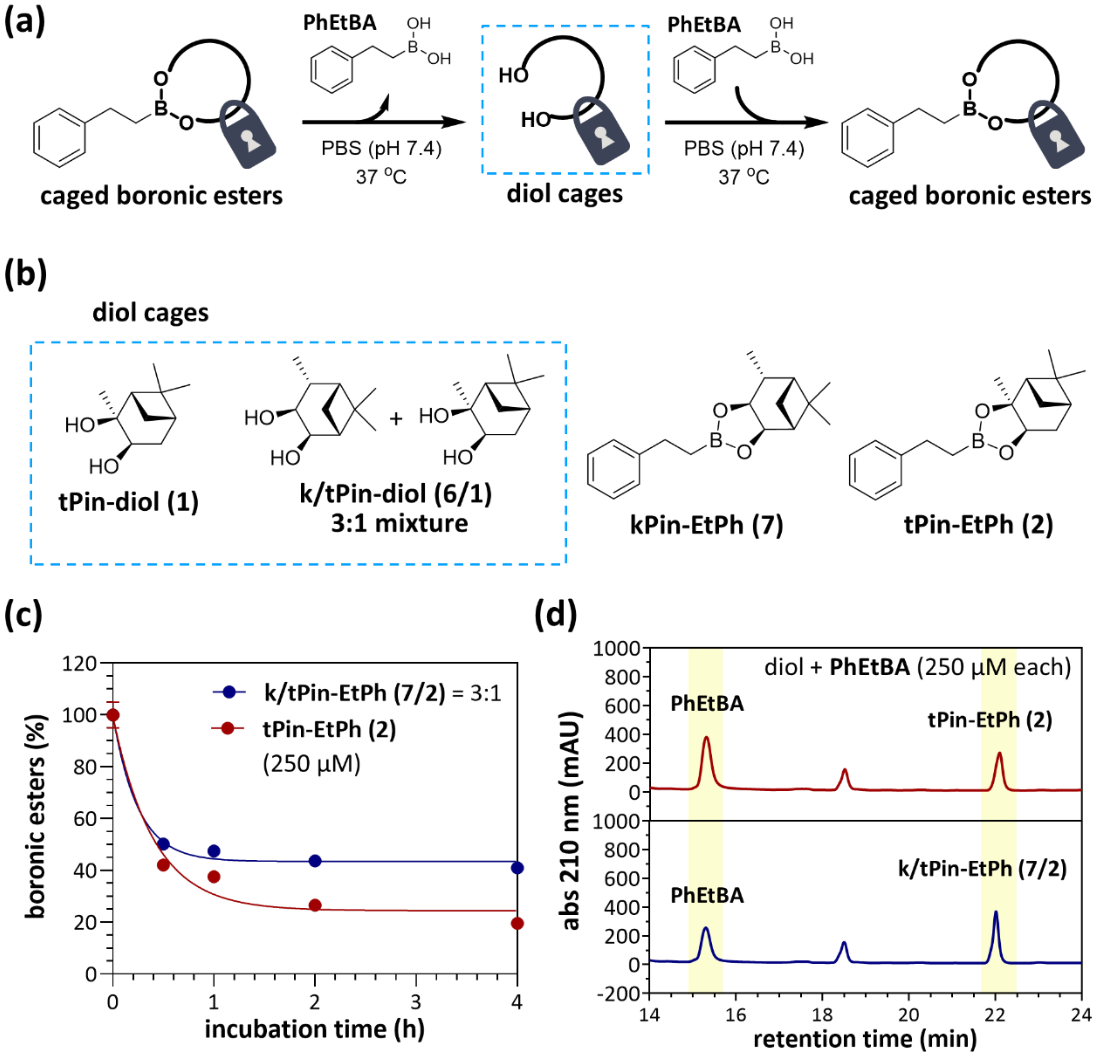

Figure S1. Validation studies of the dynamic assembly and release of pinanediol cages. (a) Schematic of the hydrolysis and assembly reactions. (b) Chemical structures of the pinanediol scaffolds and the boronic esters. (c) Quantification results of the time course of the hydrolysis assays of **tPin-EtPh (2)** and **k/tPin-EtPh (7/2)** (molar ratio of (**7**/**2**) = 3:1) (250 μM each) in PBS with 50% DMSO by HPLC. (d) HPLC of the assembly assays of **tPin-diol** and **k/tPin-diol (6/1)** (molar ratio of (**6**/**1**) = 3:1) with the boronic acid **PhEtBA** (250 μM each) in PBS with 50% DMSO at 37 °C for 4 hours.

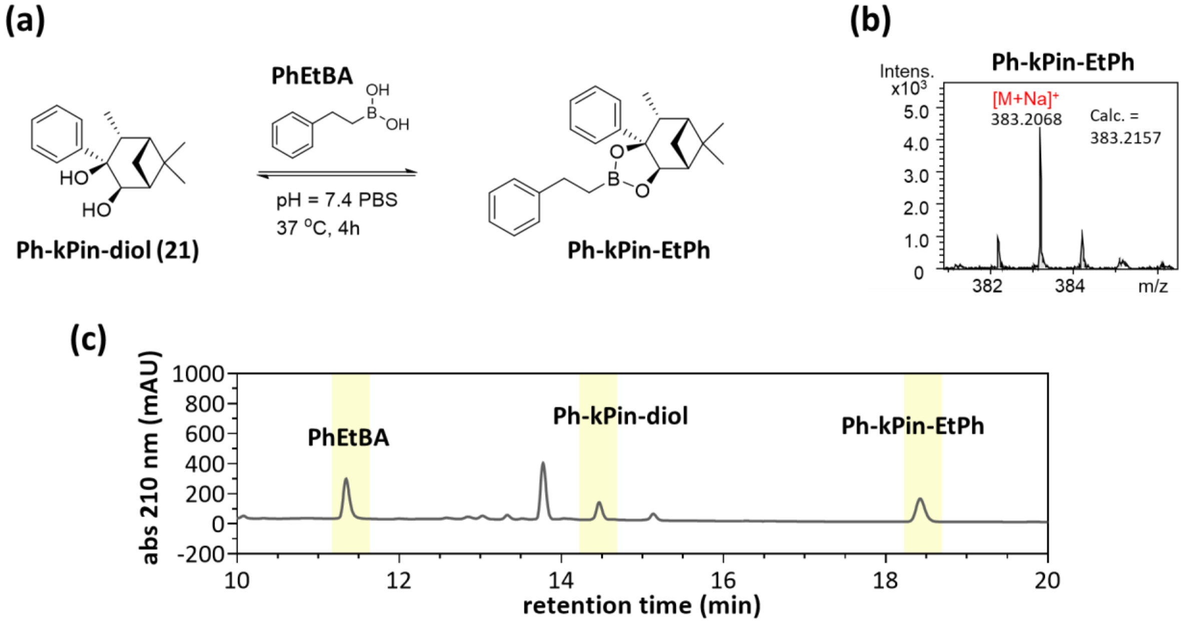

Figure S2. (a) Reactions, (b) mass spectrum, and (c) HPLC profile of assembly assay of **Ph-kPin-diol (21)** with **PhEtBA** (25 μM each) in PBS with 2% DMSO at 37°C for 4 hours.

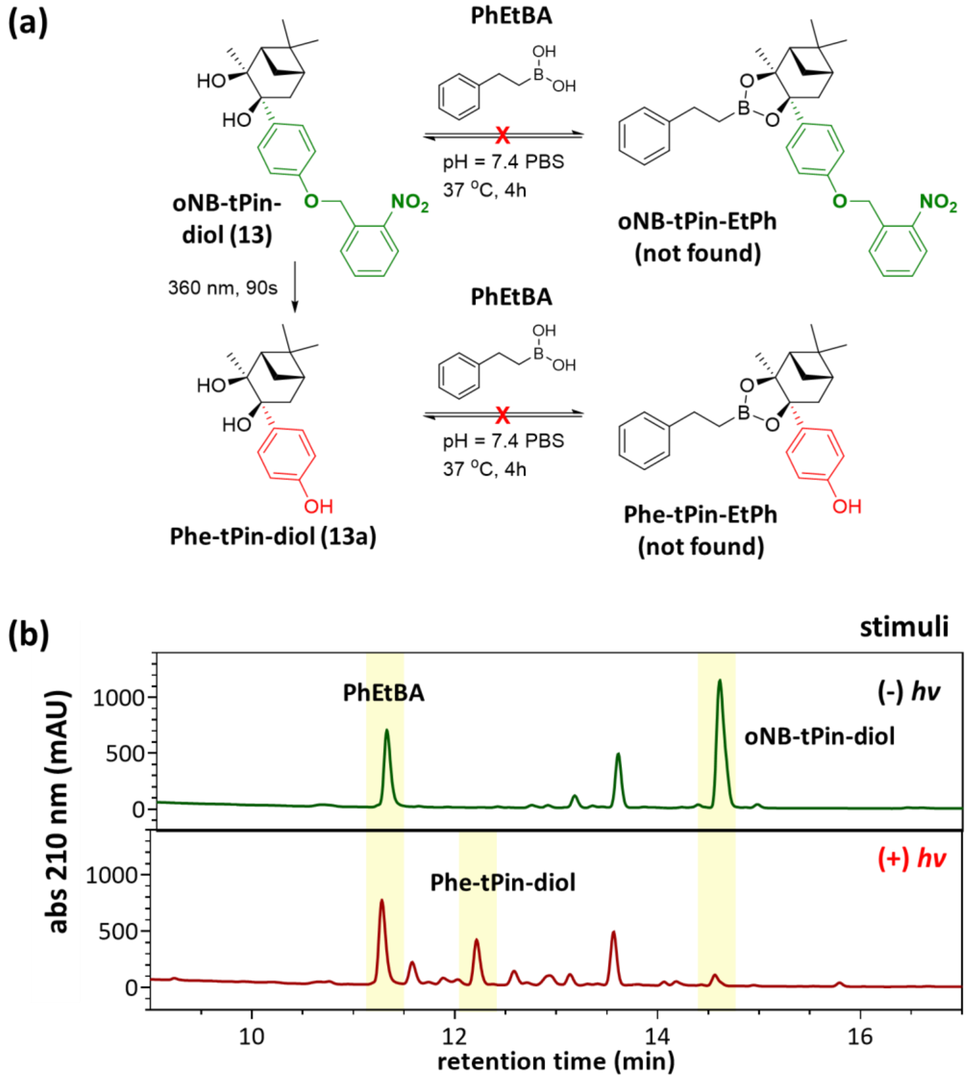

Figure S3. (a) Reactions and (b) HPLC profile of assembly assay of **oNB-tPin-diol (13)** and **Phe-tPin-diol (13a)** with **PhEtBA** (25 µM each) in PBS with 2% DMSO at 37°C for 4 hours. **13a** was obtained by *in situ* irradiation of **13** with 365 nm for 1.5 minutes.

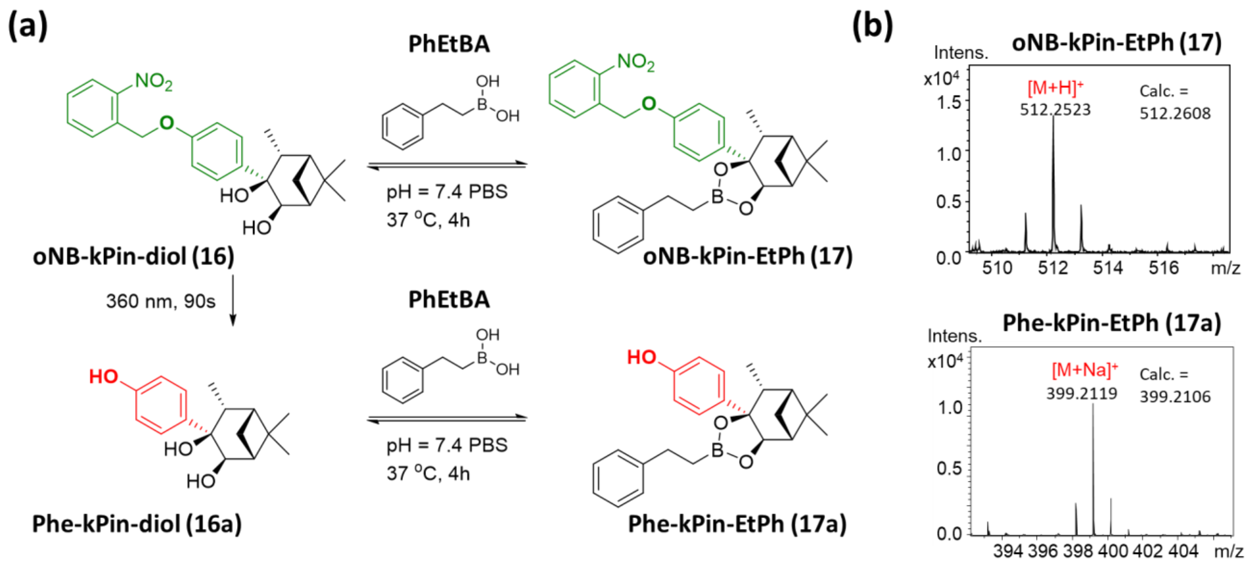

Figure S4. (a) Reactions and (b) mass spectra of assembly assay of **oNB-kPin-diol (16)** and **Phe-kPin-diol (16a)** with **PhEtBA** (25 µM each) in PBS with 2% DMSO at 37°C for 4 hours. **16a** was obtained by *in situ* irradiation of **16** with 365 nm for 1.5 minutes.

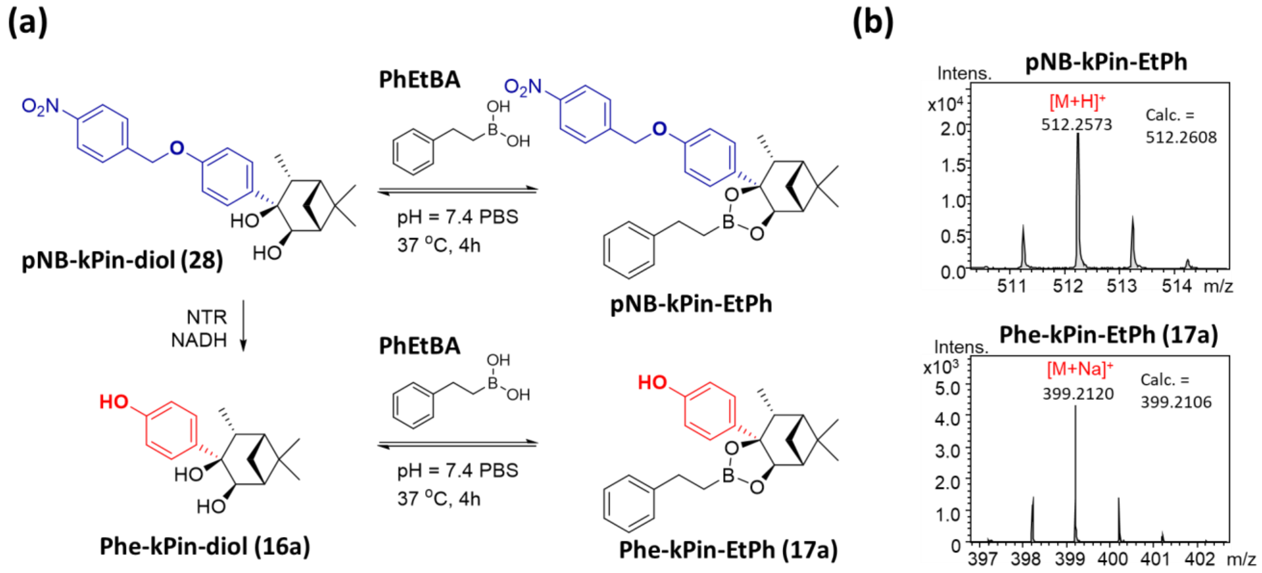

Figure S5. (a) Reactions and (b) mass spectra of assembly assay of **pNB-kPin-diol (28)** with **PhEtBA** (25 µM each) with or without NADH (250 µM) and NTR (25 µM) in PBS with 2% DMSO at 37°C for 4 hours.

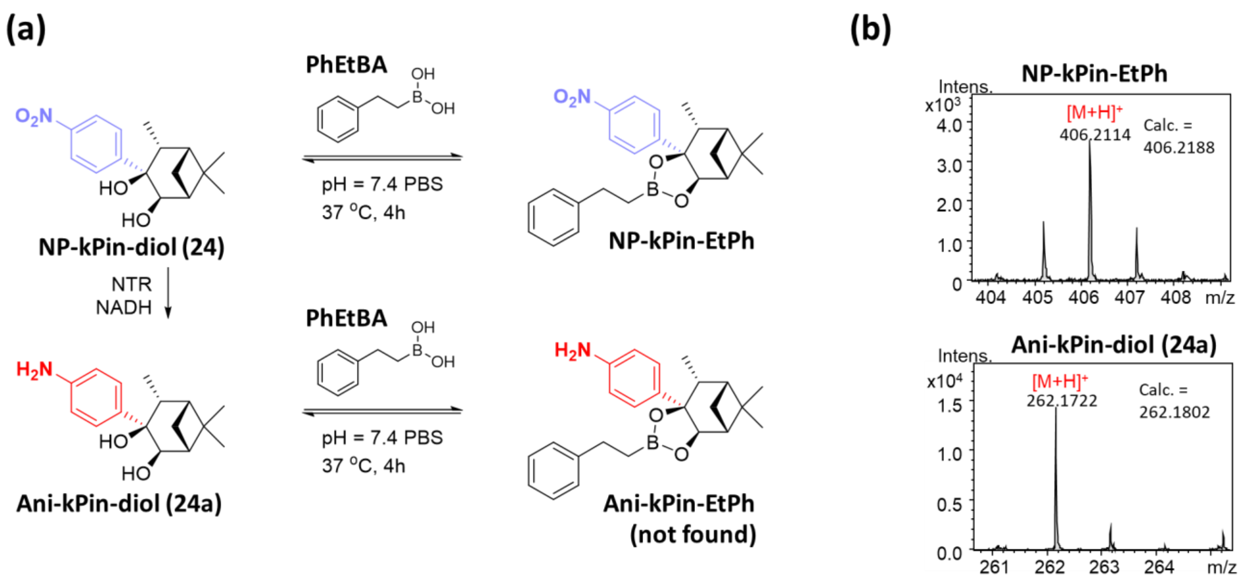

Figure S6. (a) Reactions and (b) mass spectra of **NP-kPin-diol (24)** with **PhEtBA** (25 µM each) with or without NADH (250 µM) and NTR (25 µM) in PBS with 2% DMSO at 37°C for 4 hours.

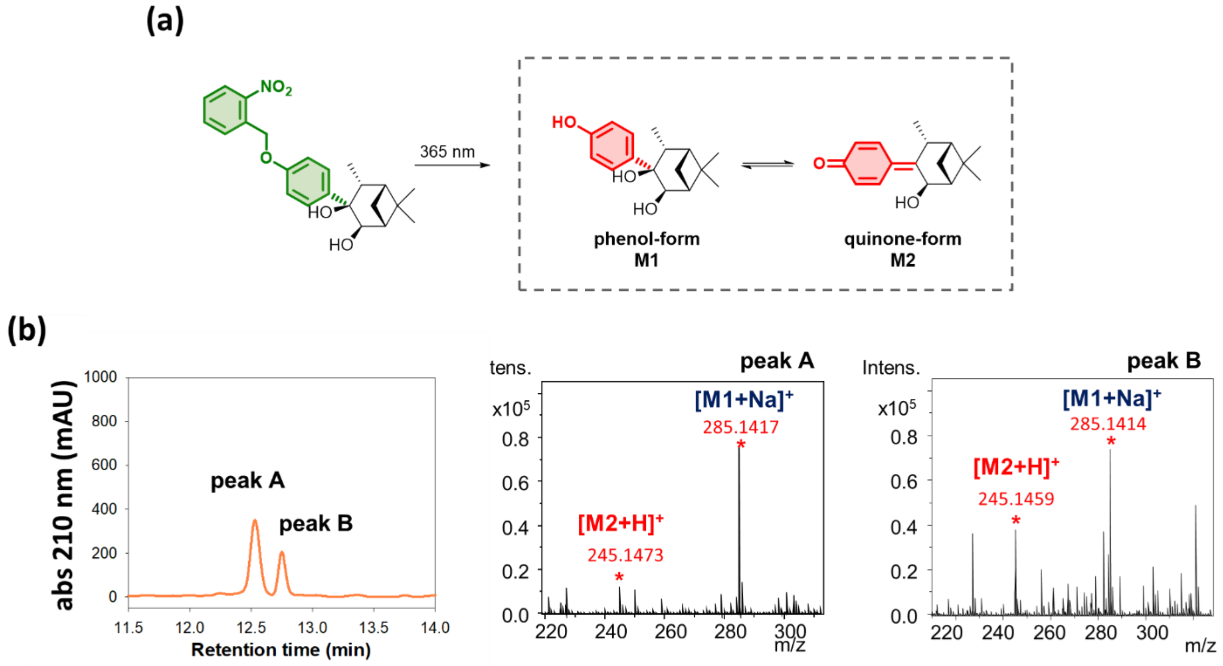

Figure S7. (a) The proposed equilibrium reaction between phenol and quinone-forms of **Phe-kPin-diol (16a)** and **(b)** LC—MS profiles of purified **16a.**

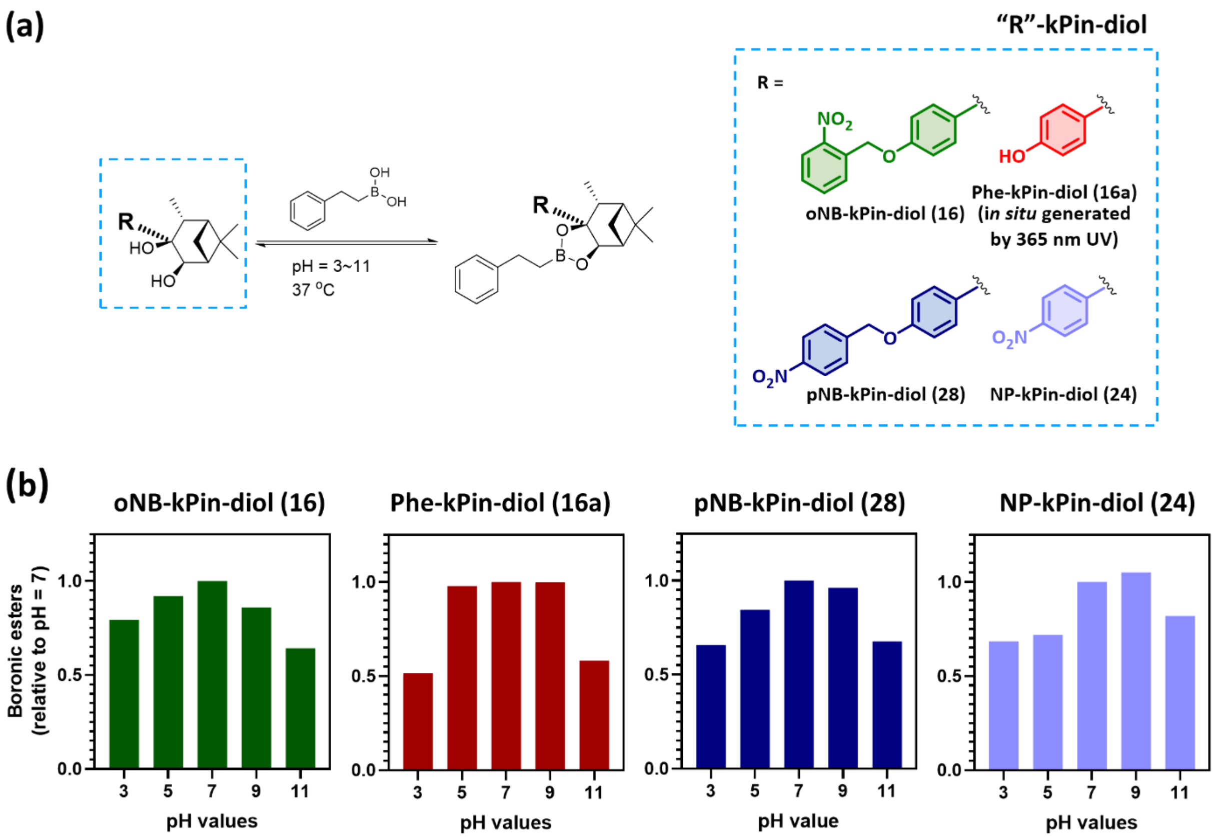

Figure S8. (a) Reactions and (b) quantification results of the assembly assays to evaluate pH-dependency of **oNB-kPin-diol (16)**, **pNB-kPin-diol (28)**, **NP-kPin-diol (24)**, and **Phe-kPin-diol (16a)** with **PhEtBA** (25 µM each) in pH buffer solutions (from pH 3 to 11) with 2% DMSO at 37°C for 4 hours.

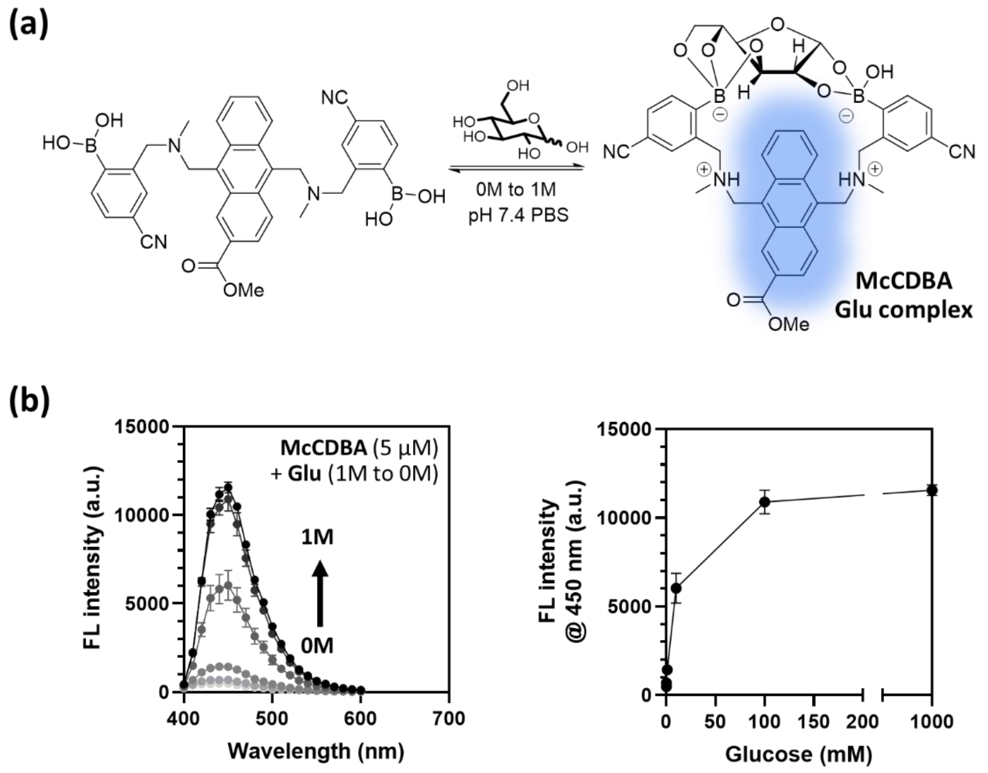

Figure S9. (a) Reactions and (b) fluorescence intensities to evaluate the fluorescence response of **McCDBA** (5 µM) with glucose (0M to 1M) in PBS with 1% DMSO at 37°C for 2 hours.

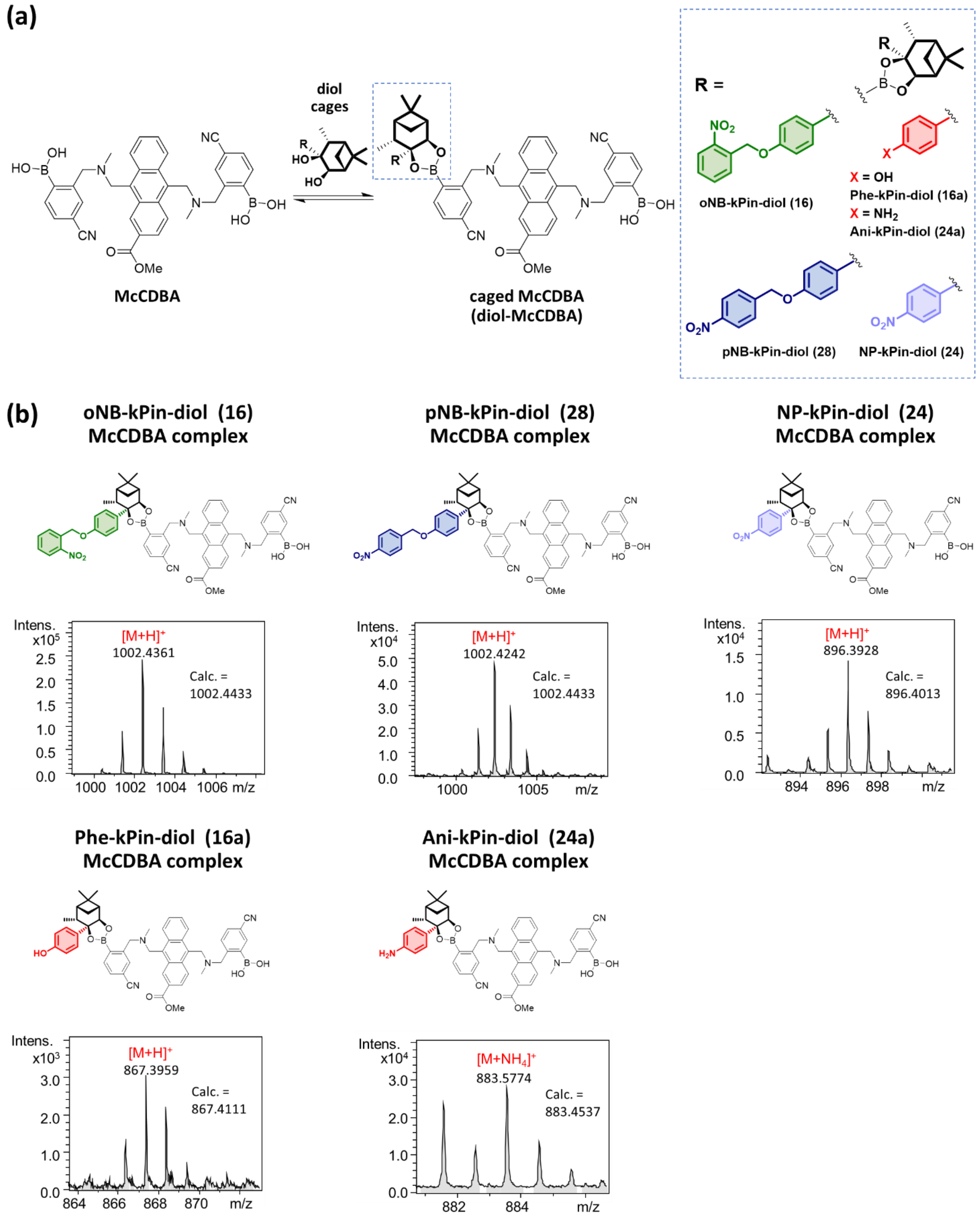

Figure S10. (a) The competition reaction between cages and glucose for forming a complex with **McCDBA**. (b) mass spectra of caged **McCDBA** with the pinanediol cages, **oNB-kPin-diol (16)**, **Phe-kPin-diol (16a)**, **pNB-kPin-diol (28)**, **NP-kPin-diol (24)**, and **Ani-kPin-diol (24a)**.

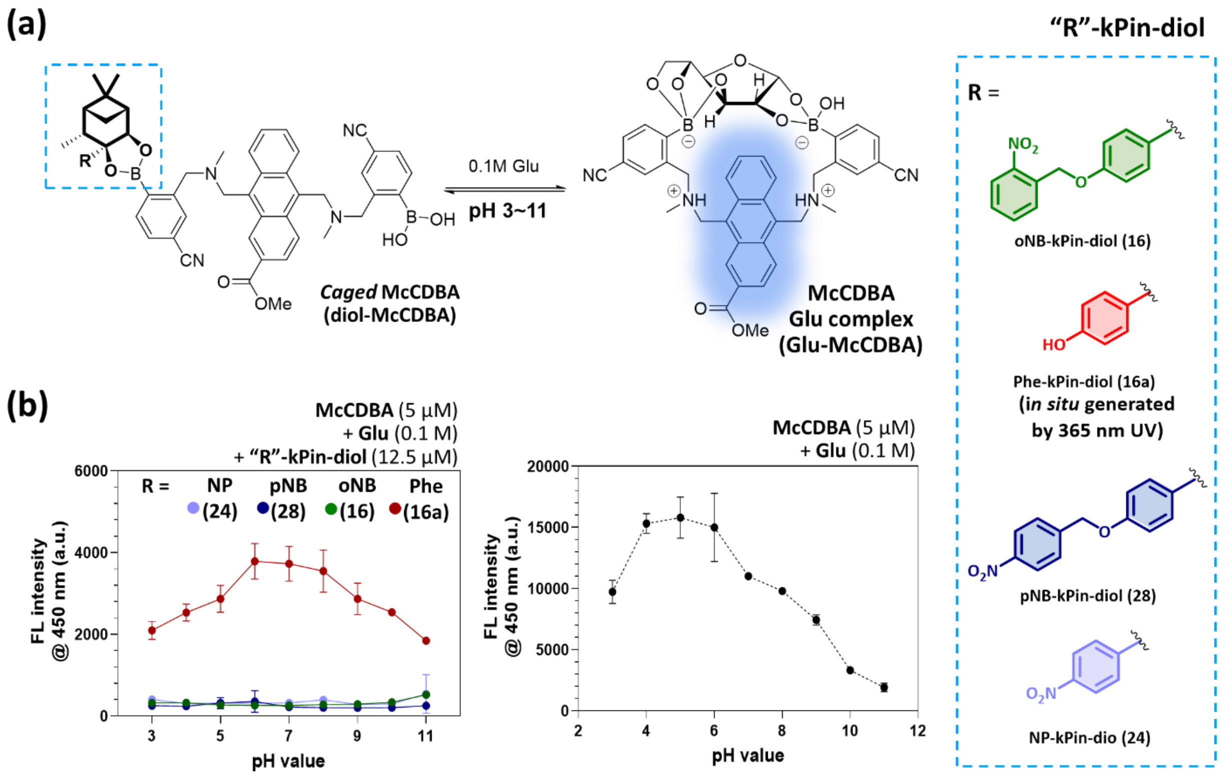

Figure S11. (a) Reactions and (b) fluorescence intensities to evaluate pH-dependency of **oNB-kPin-diol (16)**, **pNB-kPin-diol (28)**, **NP-kPin-diol (24)**, and **Phe-kPin-diol (16a)** (12.5 μM) with **McCDBA** (5 μM) in pH buffer solutions (from pH 3 to 11) with 1% DMSO and 0.1M glucose at 37°C for 2 hours.

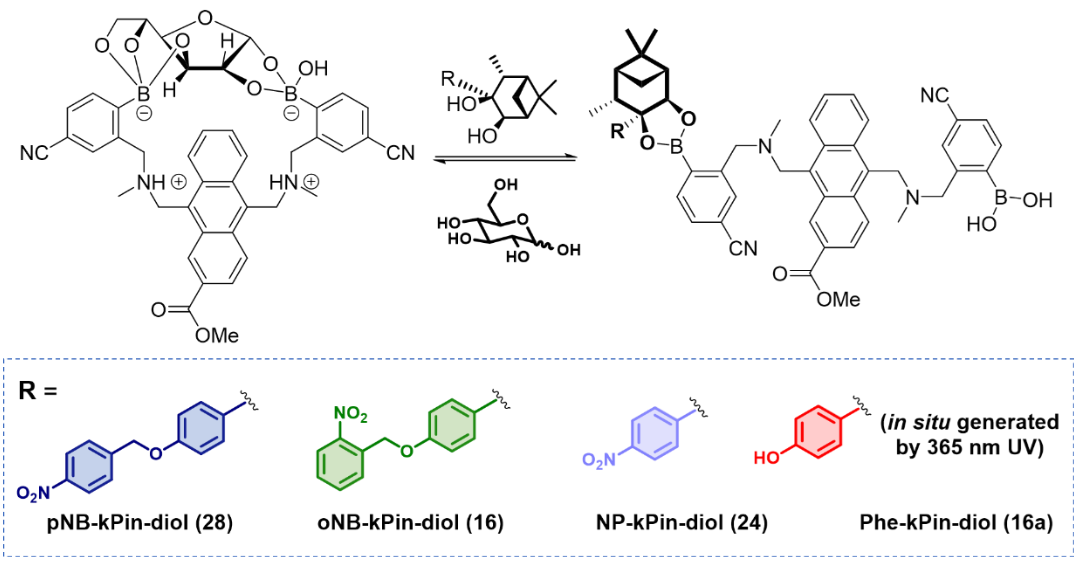

Figure S12. A simplified model of the competition between cages and glucose for the formation of a complex with **McCDBA** and chemical structures of the substituted pinanediol cages.

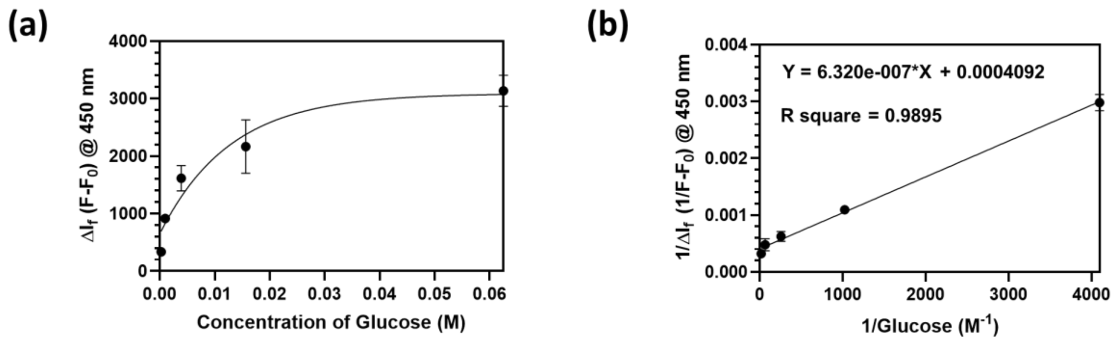

Figure S13. The fluorescence response of **McCDBA** (5 μM) with various concentrations of glucose (0 to 62.5 μM) in DMEM with 1% DMSO at 37°C for 2 hours. (a) Fluorescence changes and (b) B—H plot of the McCDBA fluorescence changes versus glucose concentrations.

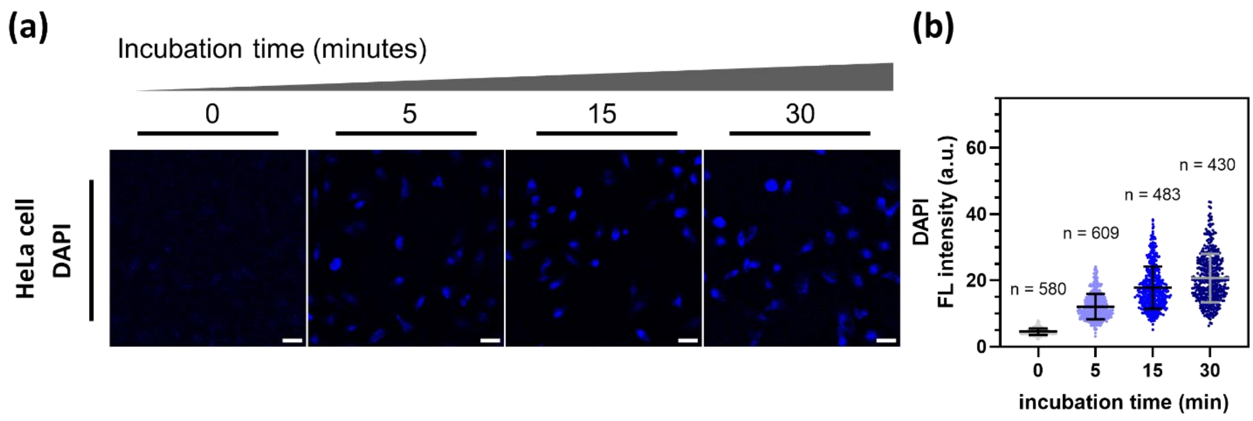

Figure S14. Evaluation of the uptake efficiency of **McCDBA** by HeLa cells. (a) Fluorescence microscopy images of HeLa cells treated with **McCDBA** (50 μM) in DMEM with 1% DMSO at 37°C (0 to 30 minutes). (b) The fluorescence intensities of **McCDBA** are shown as scatter plots, and each dot represents an individual cell. DAPI channel: fluorescence of **McCDBA**. Scale bars: 50 μm

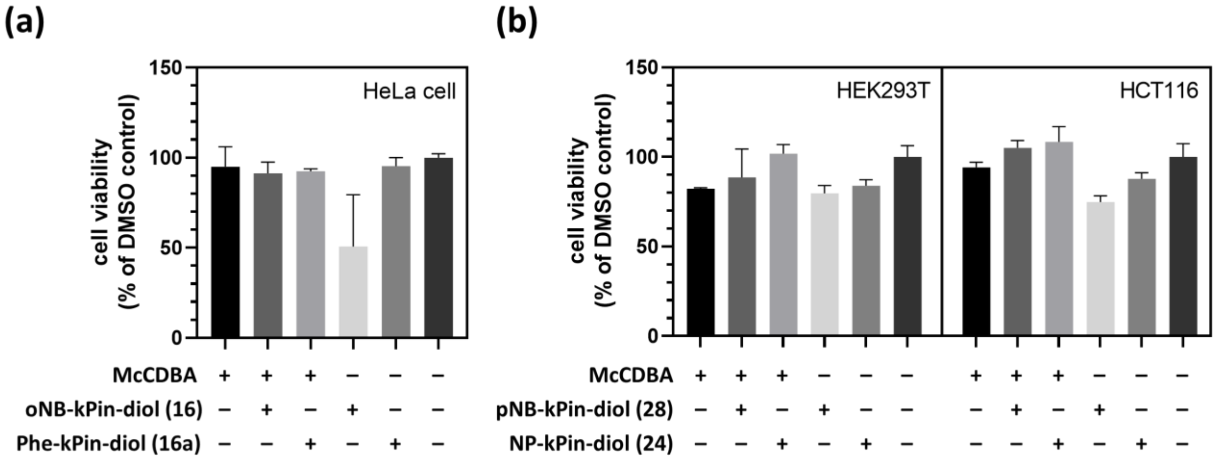

Figure S15. Cytotoxicity to HeLa, HEK293T, and HCT 116 cells. (a) Alamar Blue assay of **McCDBA**, **McCDBA**+**oNB-kPin-diol (16)**, **McCDBA**+**Phe-kPin-diol (16a)**, **16** alone, **16a** alone, and DMSO (control) (50 μM each) in HeLa cells. (b) Alamar Blue assay of **McCDBA, McCDBA**+**pNB-kPin-diol (28)**, **McCDBA**+**NP-kPin-diol (24)**, **28** alone, **24** alone, and DMSO (control) (50 μM each) in HEK293T cells and HCT116 cells.

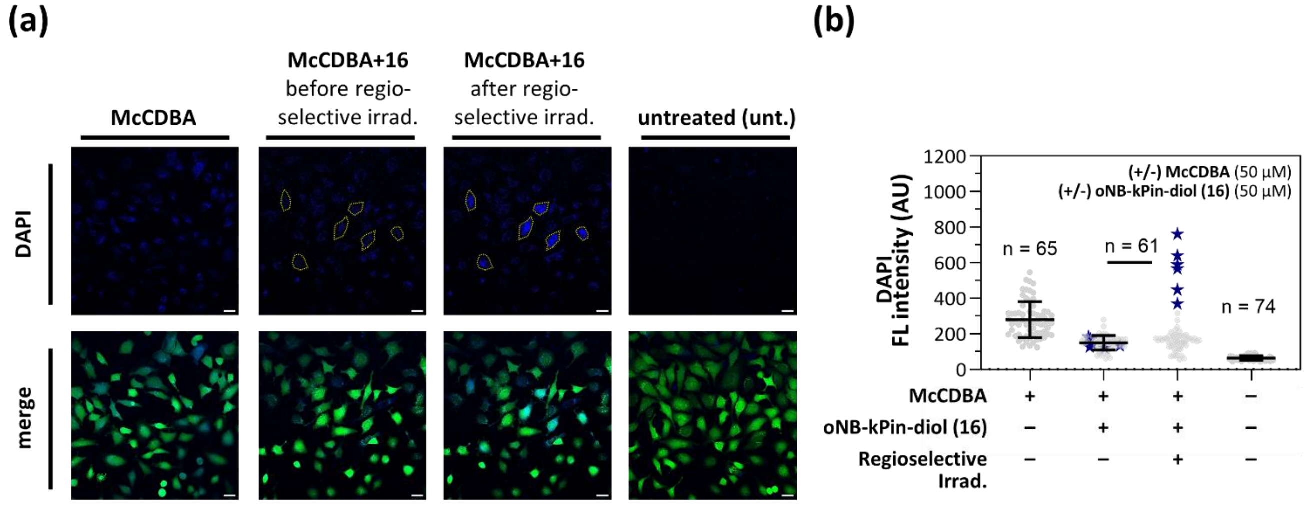

Figure S16. (a) Fluorescence microscopy images of calcein AM-stained HeLa cells treated with **McCDBA** alone (50 μM), with **McCDBA**+**oNB-kPin-diol (16)** (50 μM each) (before and after regioselective irradiation), or DMSO (unt.) in DMEM with 1% DMSO at 37°C for 30 minutes.). Yellow line-enclosed zones (six cells) indicate the regioselective irradiation areas. (b) The fluorescence intensities of **McCDBA** are shown as scatter plots, and each dot represents an individual cell. Blue stars in the scatter plots represent the six cells in yellow line-enclosed zones. DAPI channel: fluorescence of **McCDBA**. merge channel: fluorescence of **McCDBA** and calcein AM. Scale bars: 50 μm

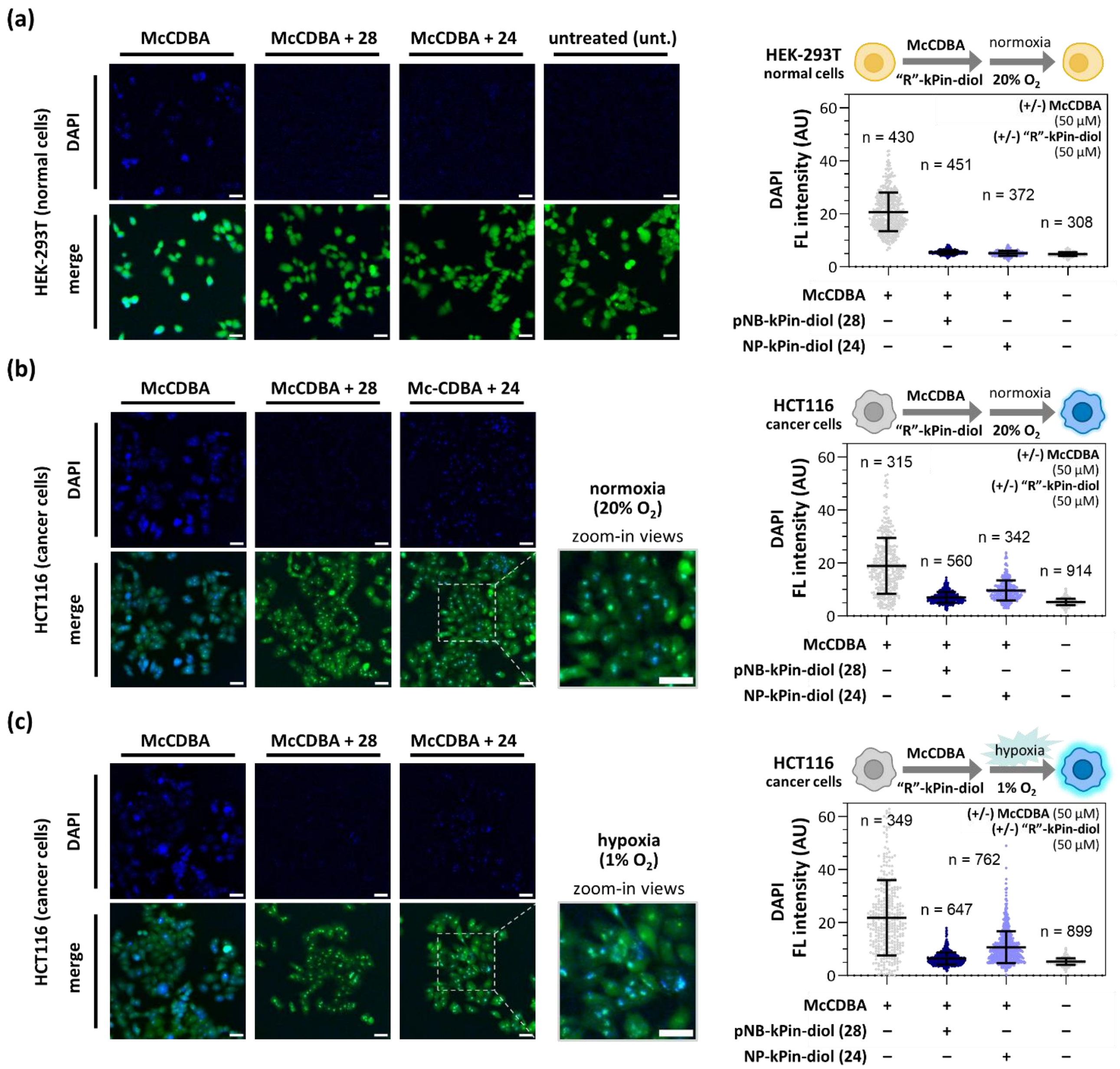

Figure S17. Demonstration of cancer cell-selective labeling by **pNB-kPin-diol (28)** and **NP-kPin-diol (24)** with **McCDBA**. Fluorescence microscopy images and scatter plots of calcein AM-stained (a) HEK293T cells under 20% O_2, (b) HCT116 cells under 20% O_2, and (c) HCT116 cells under 1% O_2. In each group, the cells were treated with **McCDBA** alone (50 µM), **McCDBA**+**28** (50 µM each), **McCDBA**+**24** (50 µM each), or DMSO (unt.) in DMEM with 1% DMSO at 37 °C for 30 minutes and further incubated for 8 hours. The white boxes indicate the zoomed-in views of selected HCT116 and HEK293T cells. BF channel: bright field; DAPI channel: fluorescence of **McCDBA**; merge channel: fluorescence of **McCDBA** and calcein AM. Scale bars: 50 µm. Each dot in the scatter plots represents an individual cell.

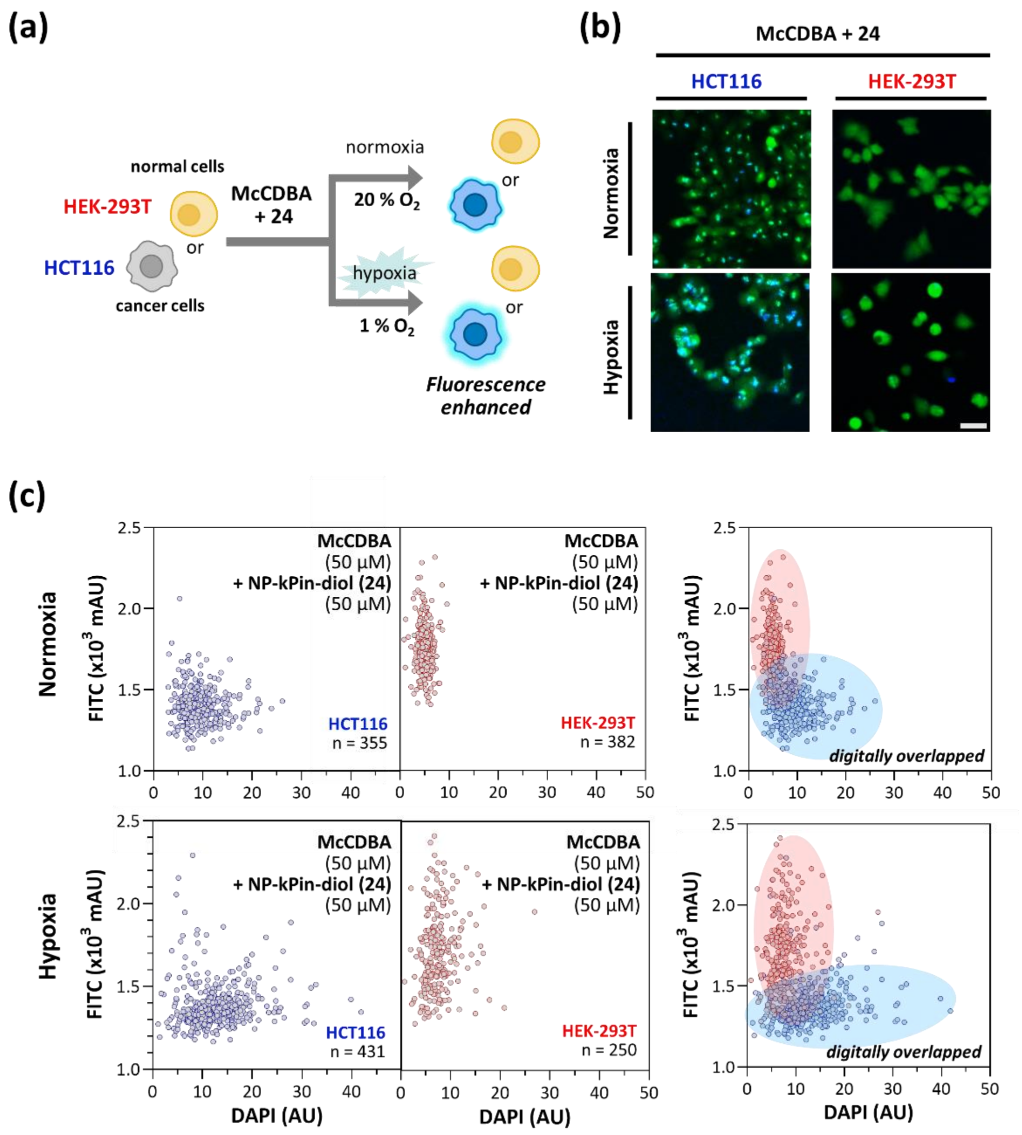

Figure S18. (a) Schematic to illustrate the fluorescence enhancement under hypoxia by the NTR-responsive pinanediol cages with **McCDBA**. (b) Fluorescence microscopy images of calcein AM-stained HEK293T and HCT116 cells treated with **McCDBA**+**NP-kPin-diol (24)** (50 µM each) in DMEM with 1% DMSO at 37°C for 30 minutes and further incubated for 8 hours (20% or 1% O_2). (c) Scatter plots of the cellular intensities, each dot represents an individual cell. FITC channel: fluorescence of calcein AM; DAPI channel: fluorescence of **McCDBA**. Red area: the distribution of HEK293T cells; Blue area: the distribution of HCT116 cells; Scale bars: 50 µm.

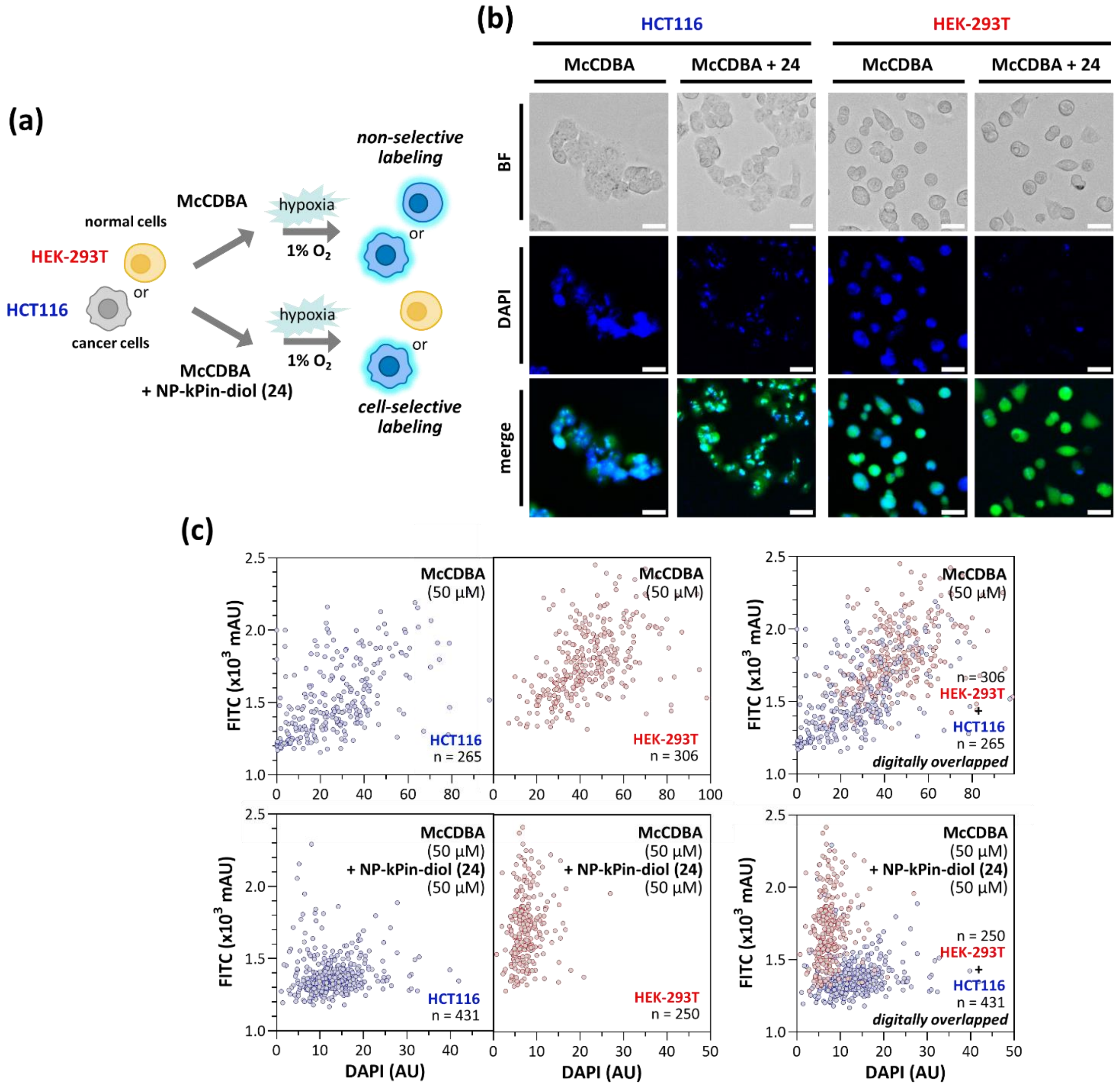

Figure S19. (a) Schematic to illustrate cancer cell-selective labeling by the NTR-responsive pinanediol cages with **McCDBA**. (b) Fluorescence microscopy images of calcein AM-stained HEK293T and HCT116 cells treated with **McCDBA** alone (50 µM) or with **McCDBA**+**NP-kPin-diol (24)** (50 µM each) in DMEM with 1% DMSO at 37°C for 30 minutes and further incubated for 8 hours (1% O_2). (c) Scatter plots of the cellular intensities, each dot represents an individual cell. BF channel: bright field; FITC channel: fluorescence of calcein AM; DAPI channel: fluorescence of **McCDBA**; merge channel: fluorescence of **McCDBA** and calcein AM. Scale bars: 50 µm.

7. Appendix: NMR Spectroscopic Data

tPin-EtPh (2) ^{1}H NMR, $CDCl_3$

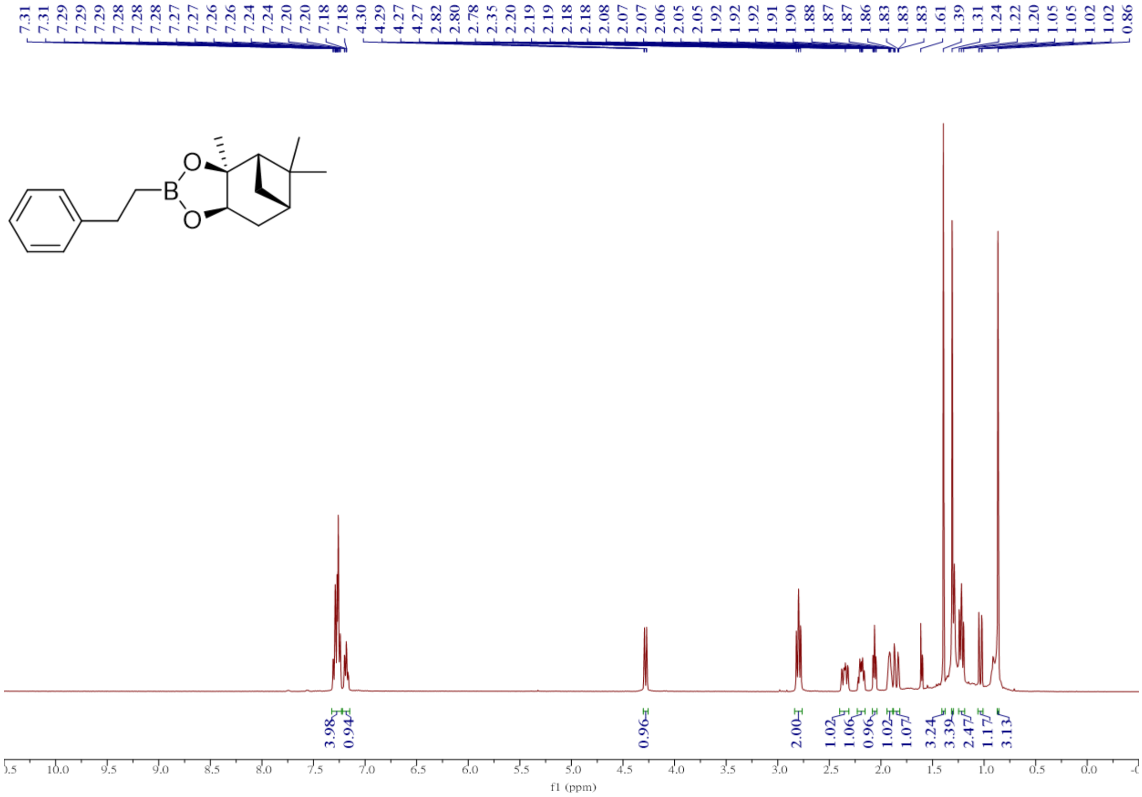

tPin-EtPh (2) ^{13}C NMR, $CDCl_3$

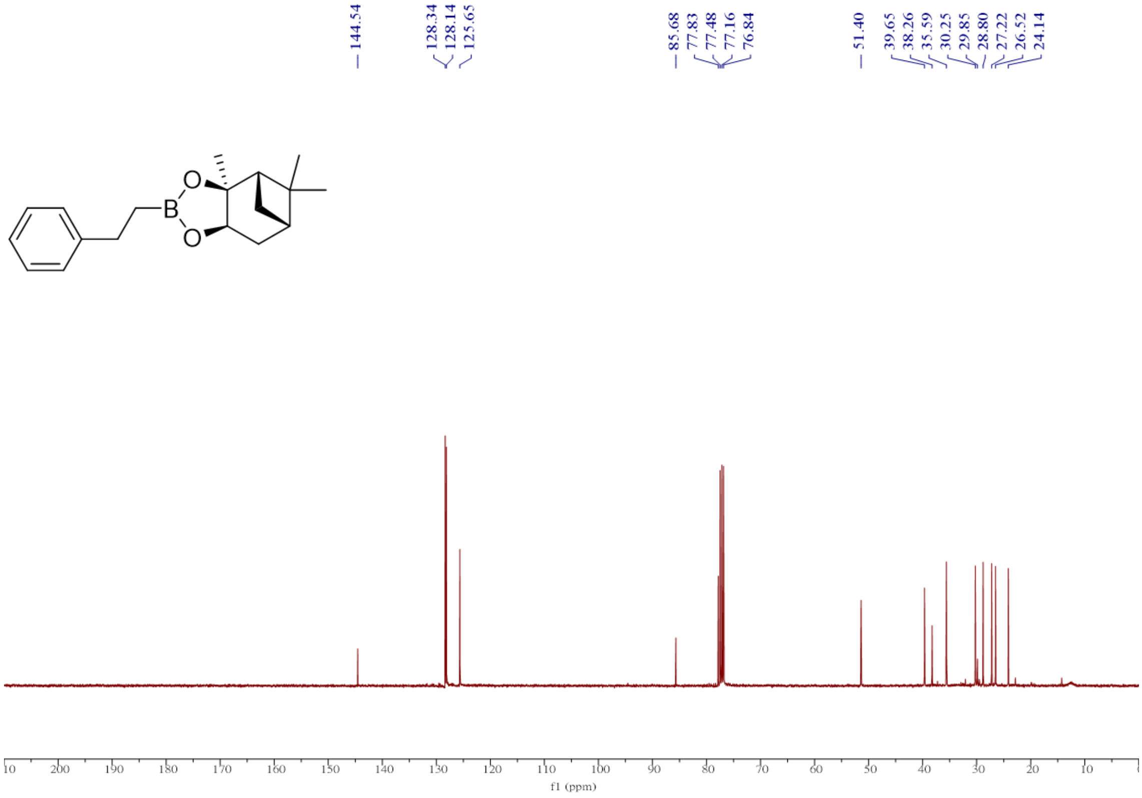

Pinanol (3) ^{1}H NMR, $CDCl_3$

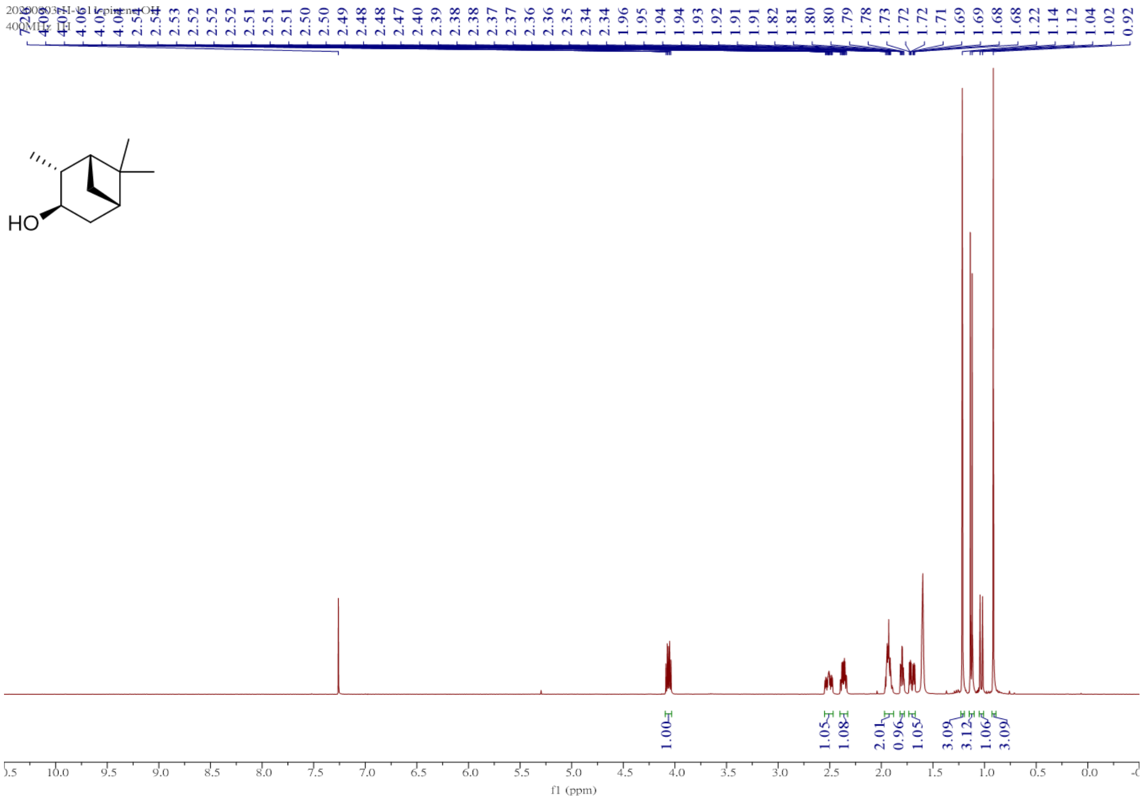

Pinanol (3) ^{13}C NMR, $CDCl_3$

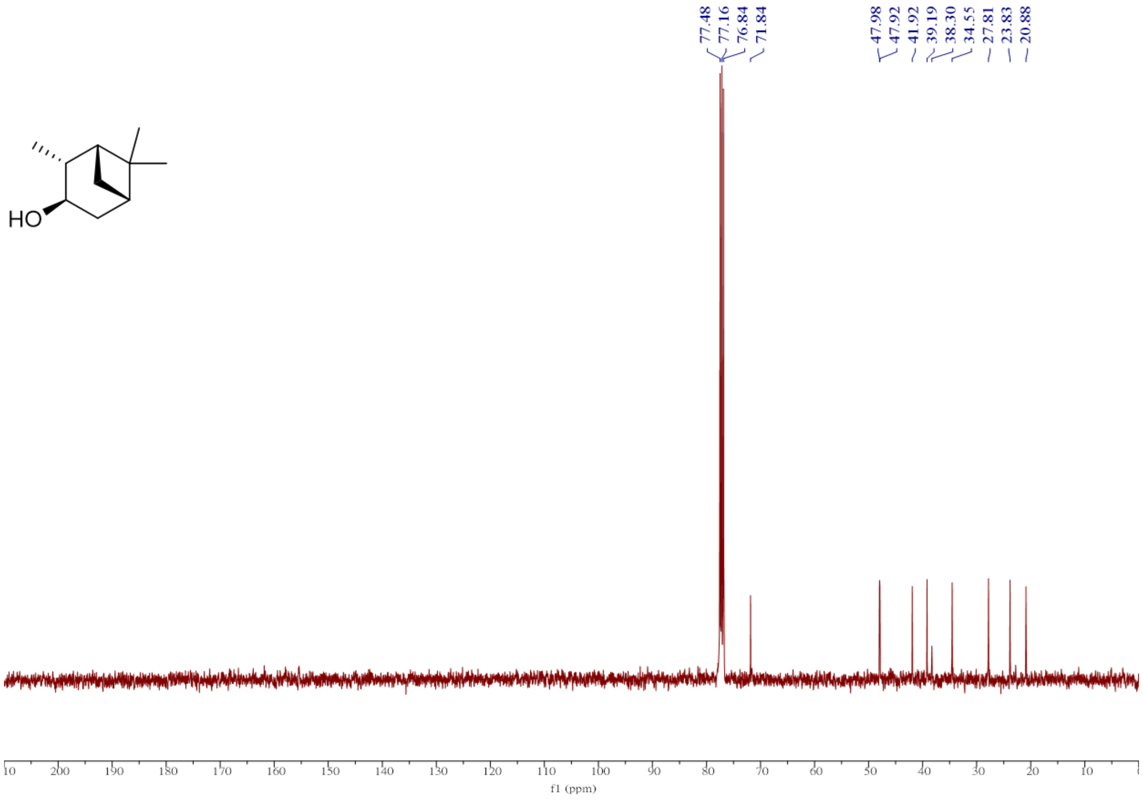

OTs-Pin (4) ^{1}H NMR, $CDCl_3$

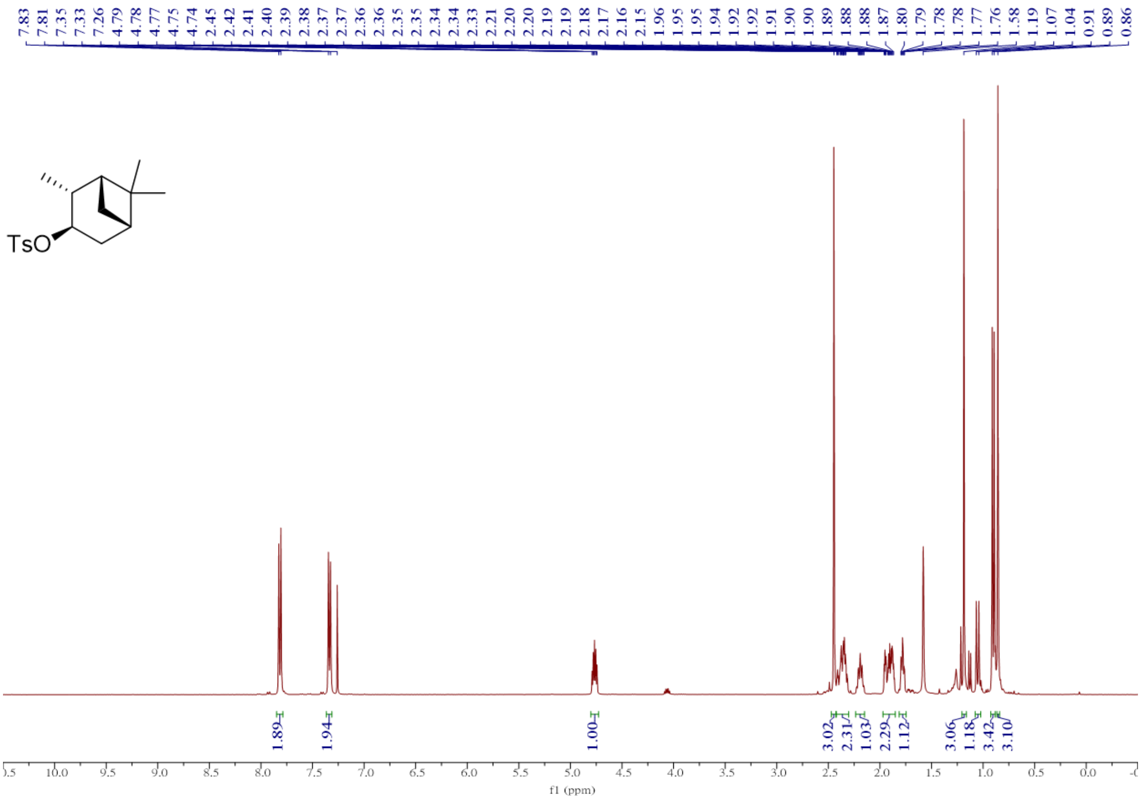

OTs-Pin (4) ^{13}C NMR, $CDCl_3$

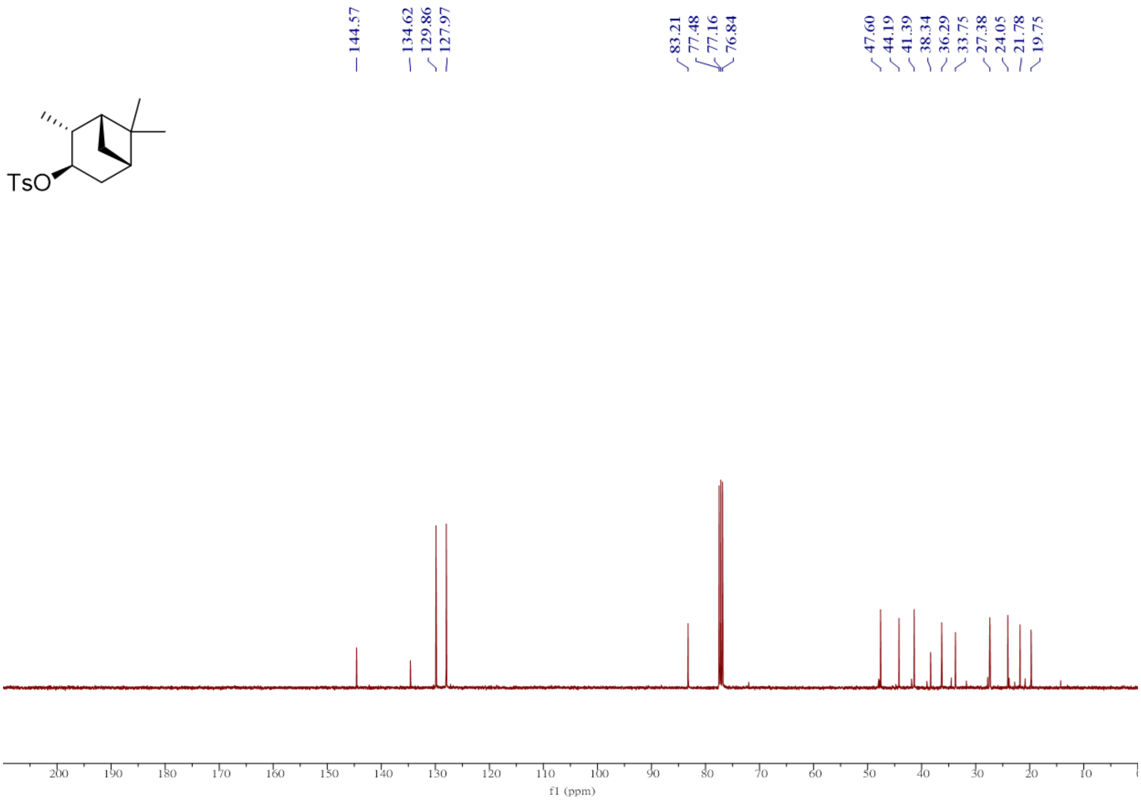

δ-pinene (5) ^{1}H NMR, $CDCl_3$

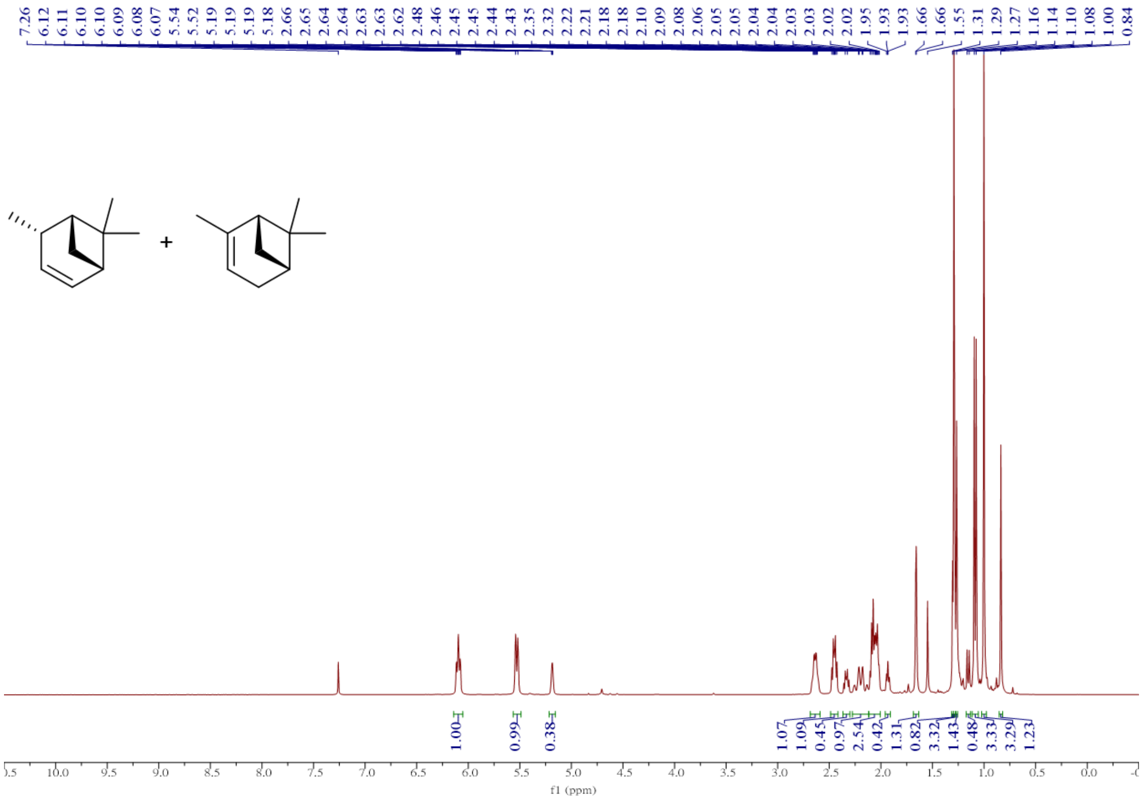

δ-pinene (5) ^{13}C NMR, $CDCl_3$

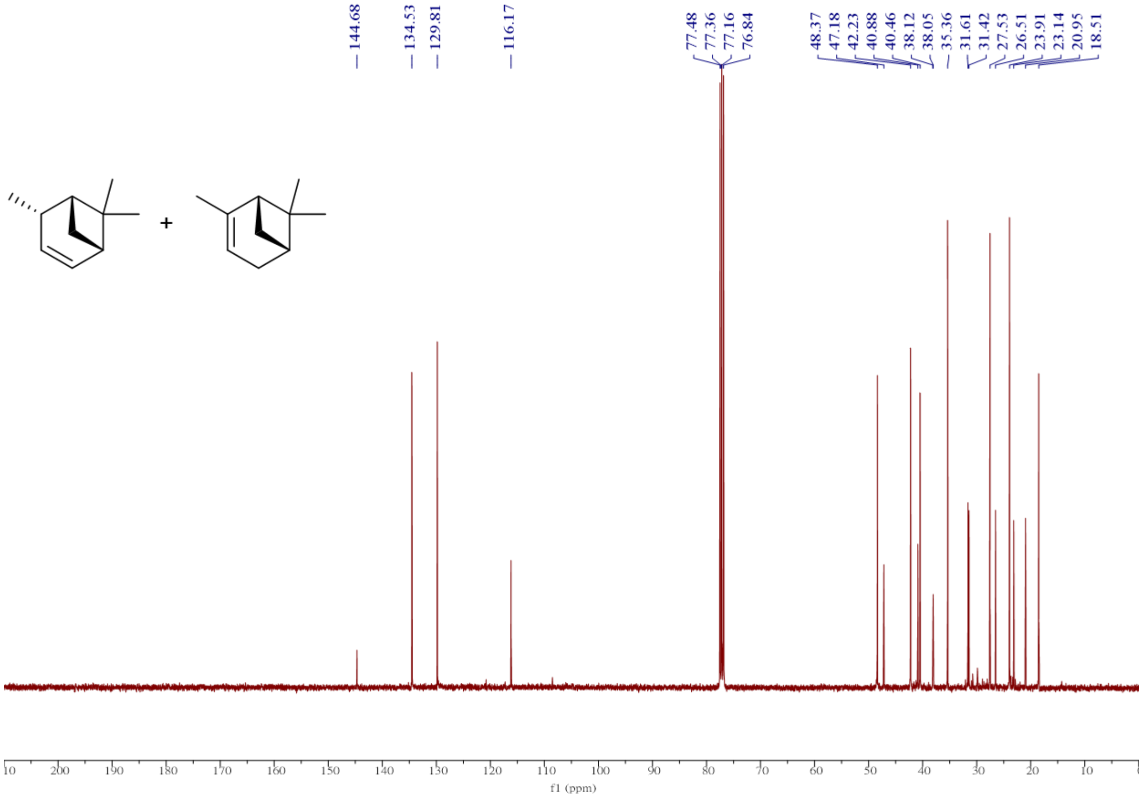

kPin-diol (6) ^{1}H NMR, $CDCl_3$

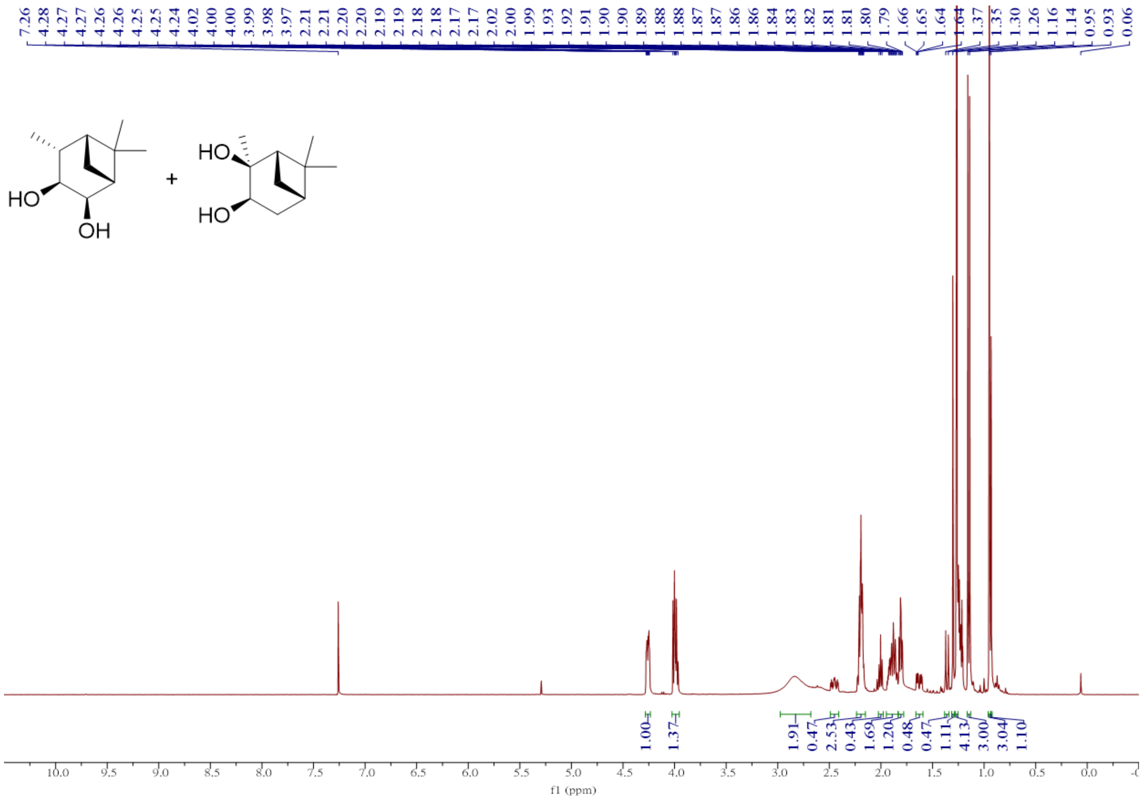

kPin-diol (6) ^{13}C NMR, $CDCl_3$

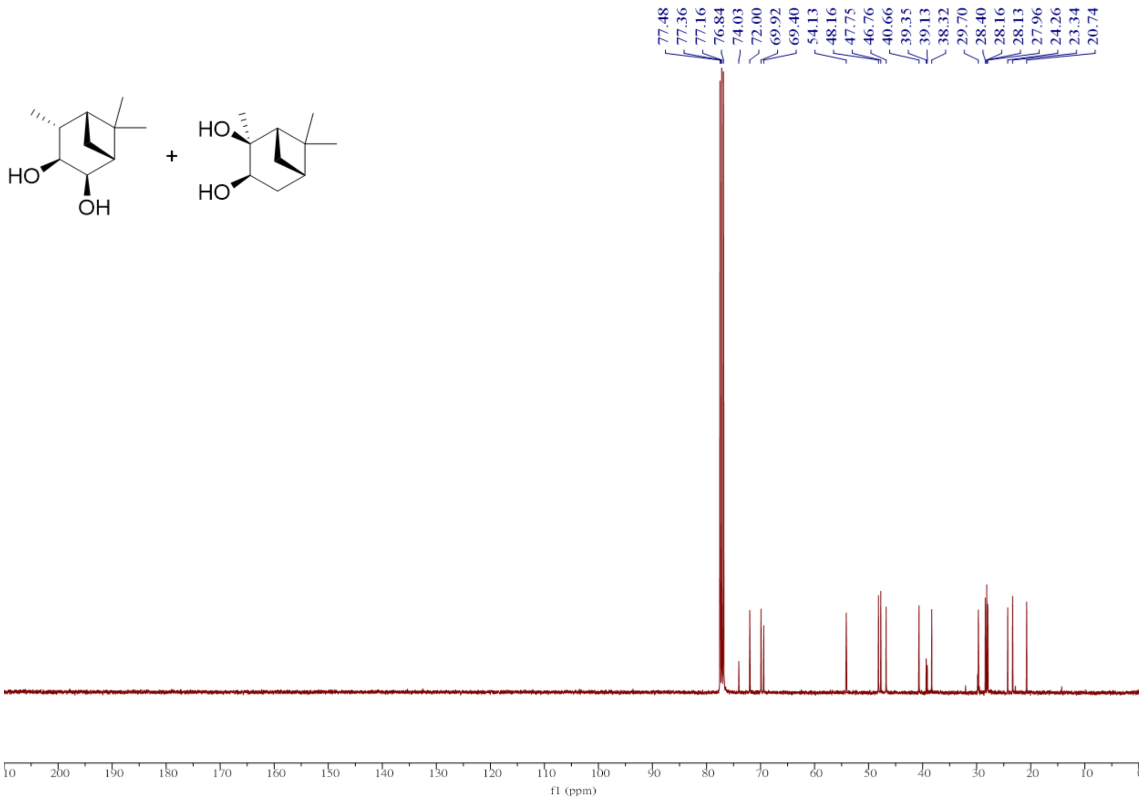

kPin-EtPh (7) ^{1}H NMR, $CDCl_3$

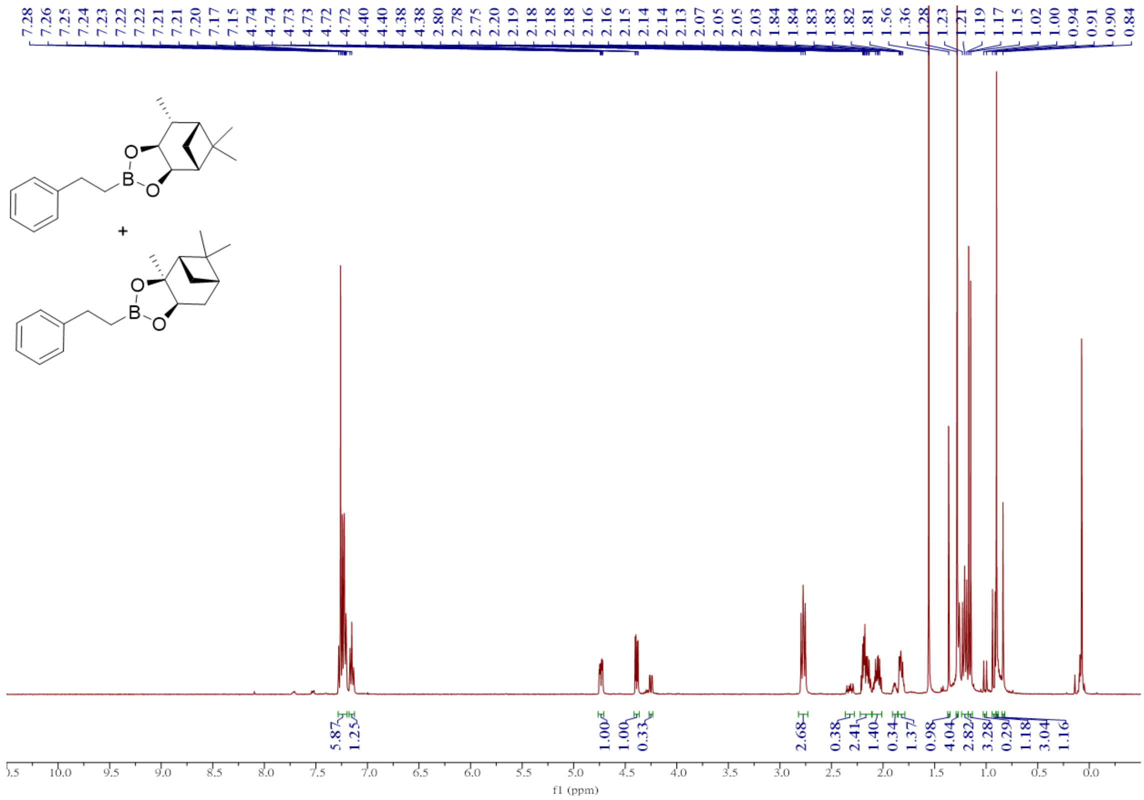

kPin-EtPh (7) ^{13}C NMR, $CDCl_3$

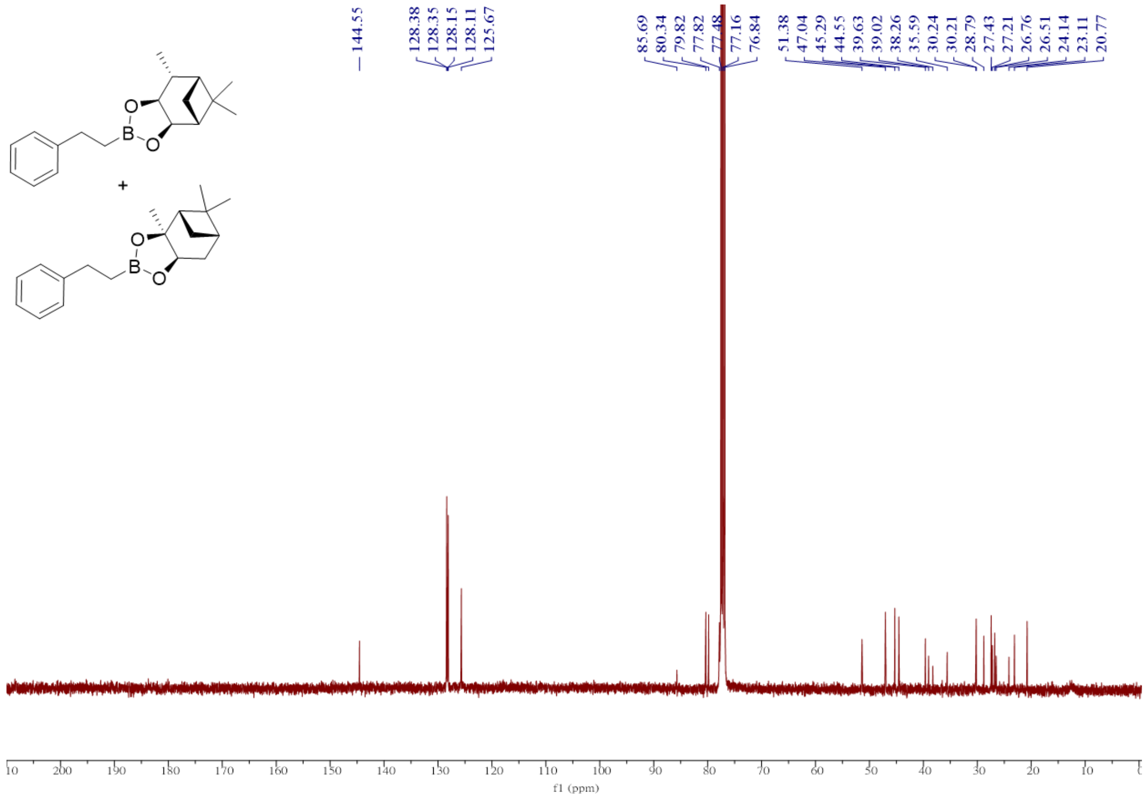

oNB-Br (8) ^{1}H NMR, $CDCl_3$

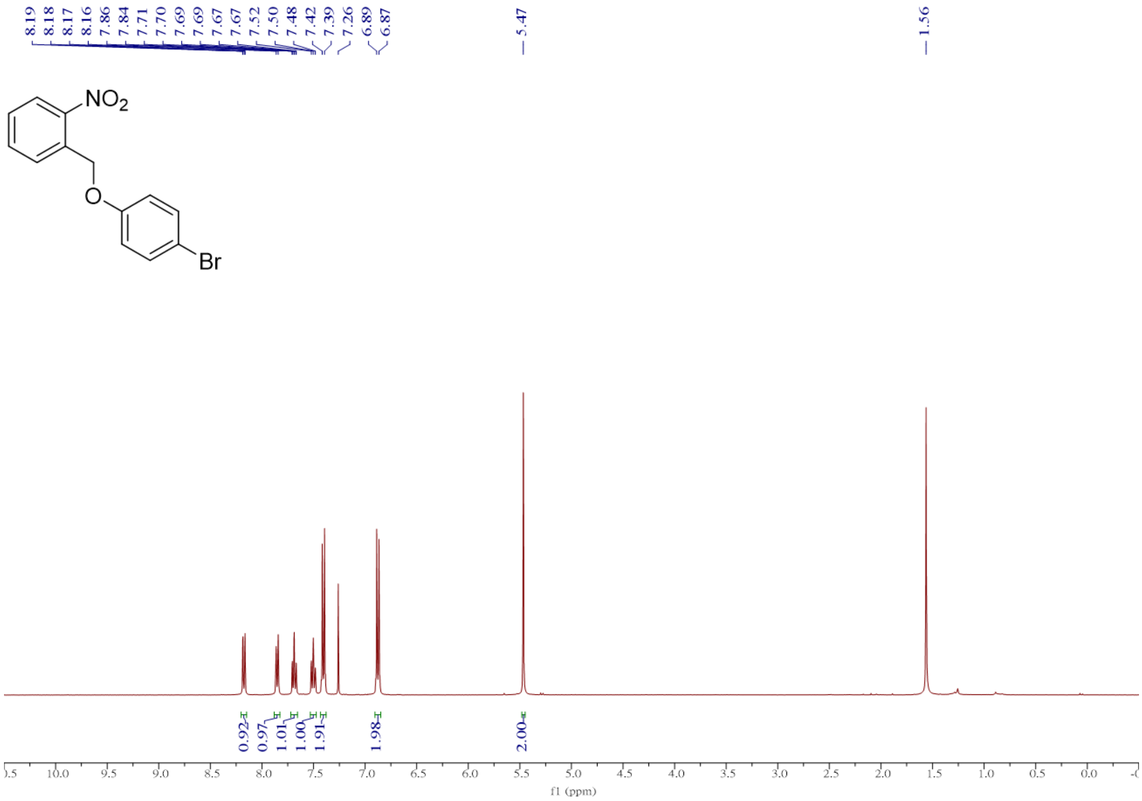

oNB-Br (8) ^{13}C NMR, $CDCl_3$

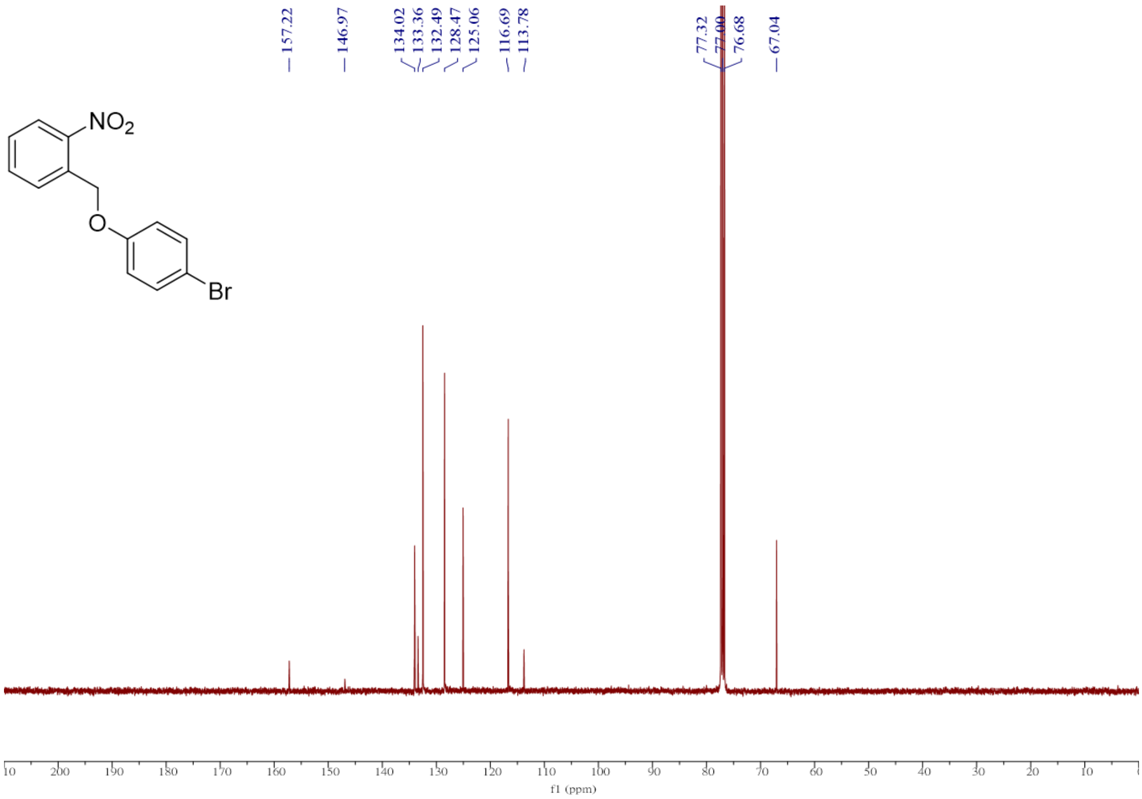

oNB-Pinacol (9) ^{1}H NMR, $CDCl_3$

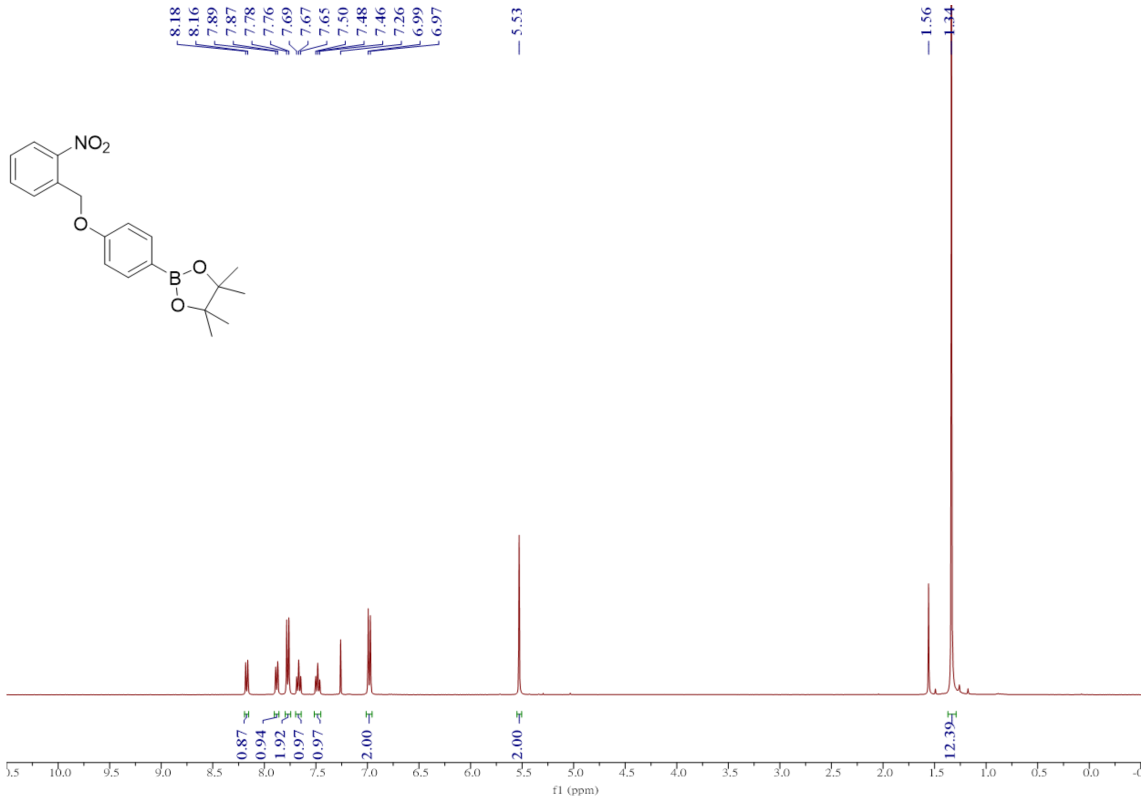

oNB-Pinacol (9) ^{13}C NMR, $CDCl_3$

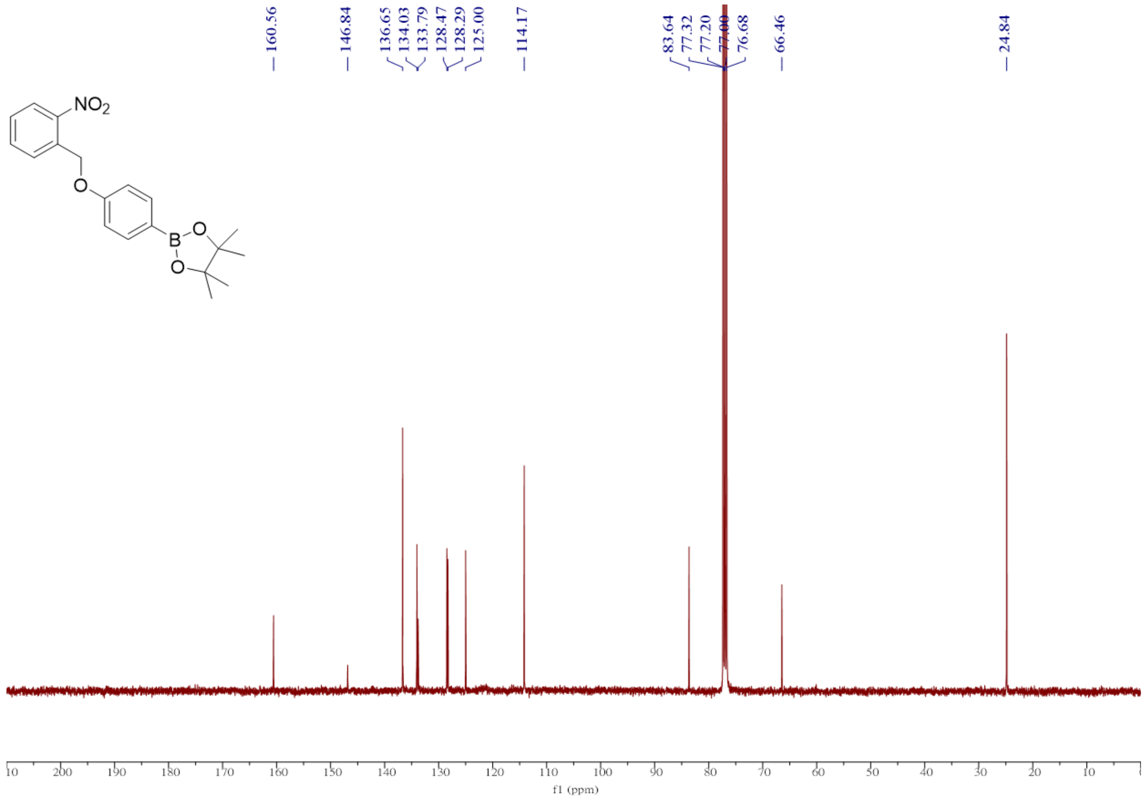

pinanone (10) ^{1}H NMR, $CDCl_3$

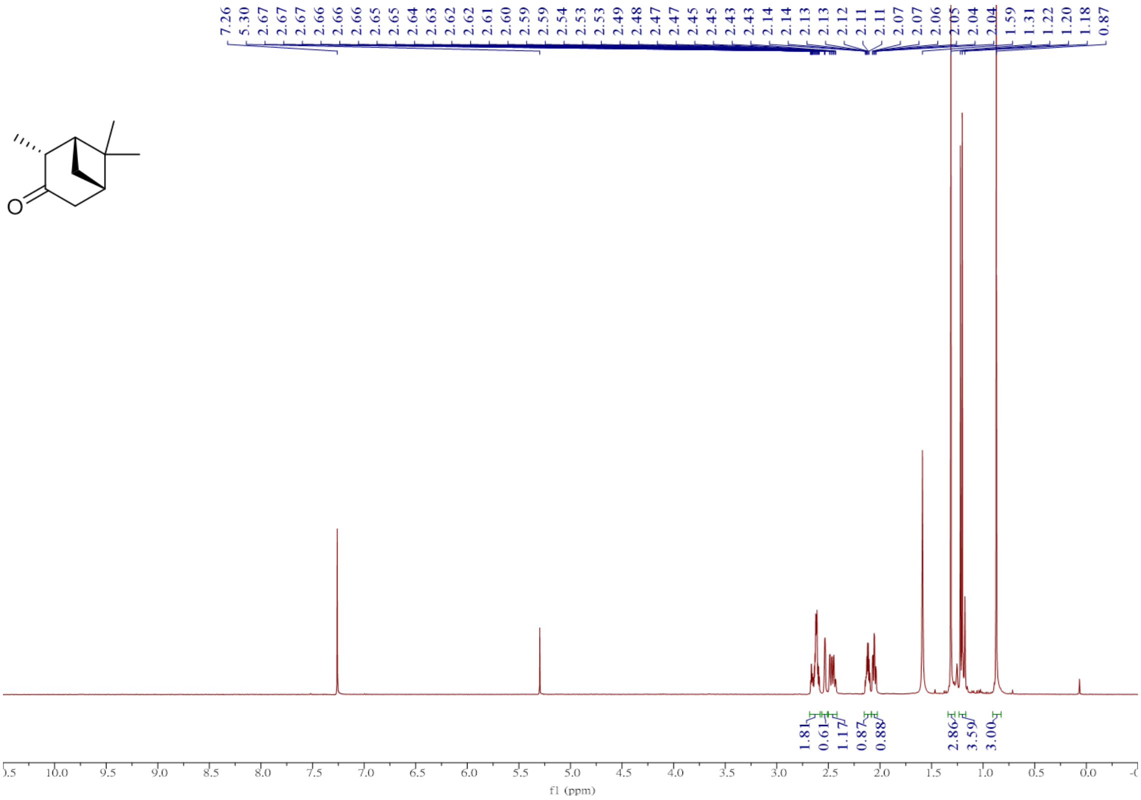

pinanone (10) ^{13}C NMR, $CDCl_3$

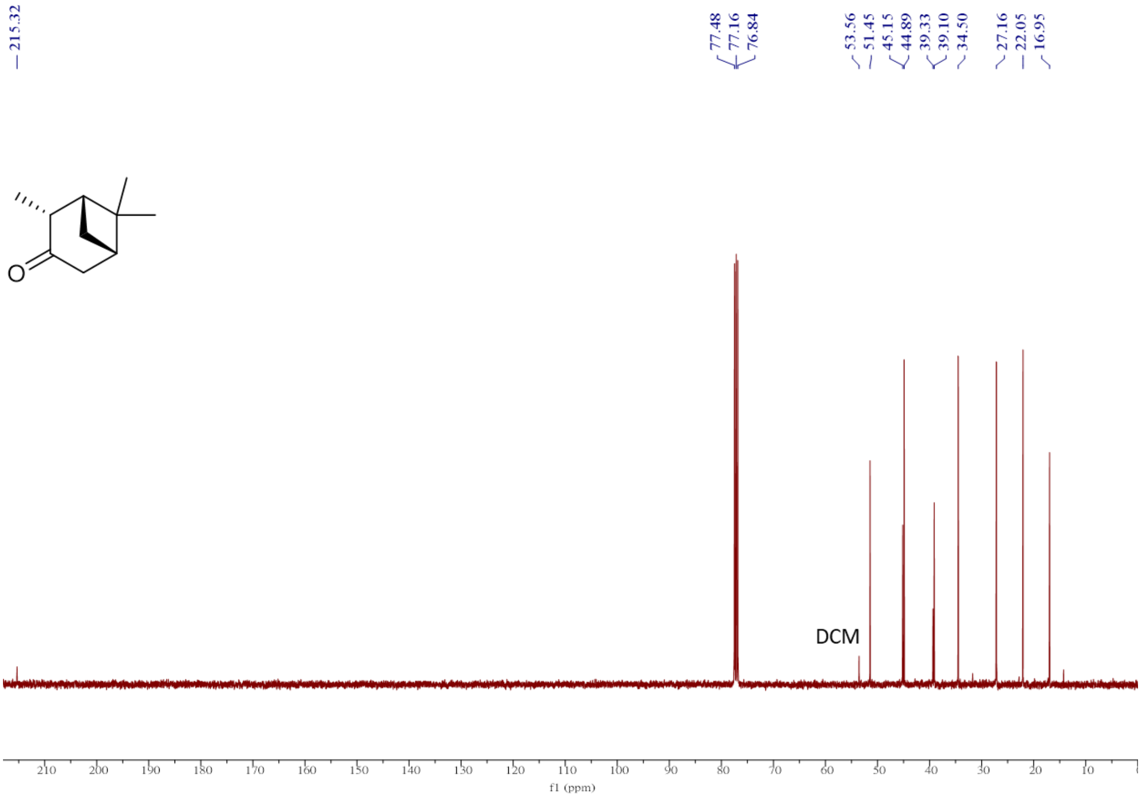

OTf-tPin (11) ^{1}H NMR, $CDCl_3$

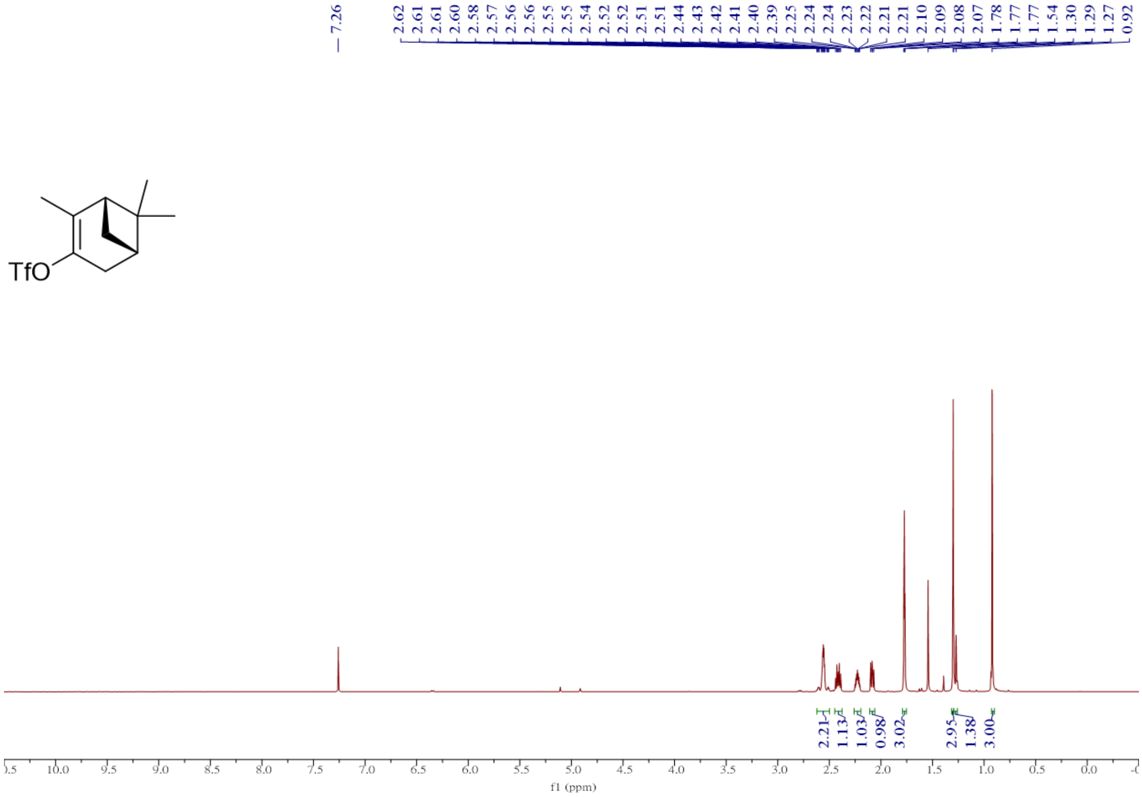

OTf-tPin (11) ^{13}C NMR, $CDCl_3$

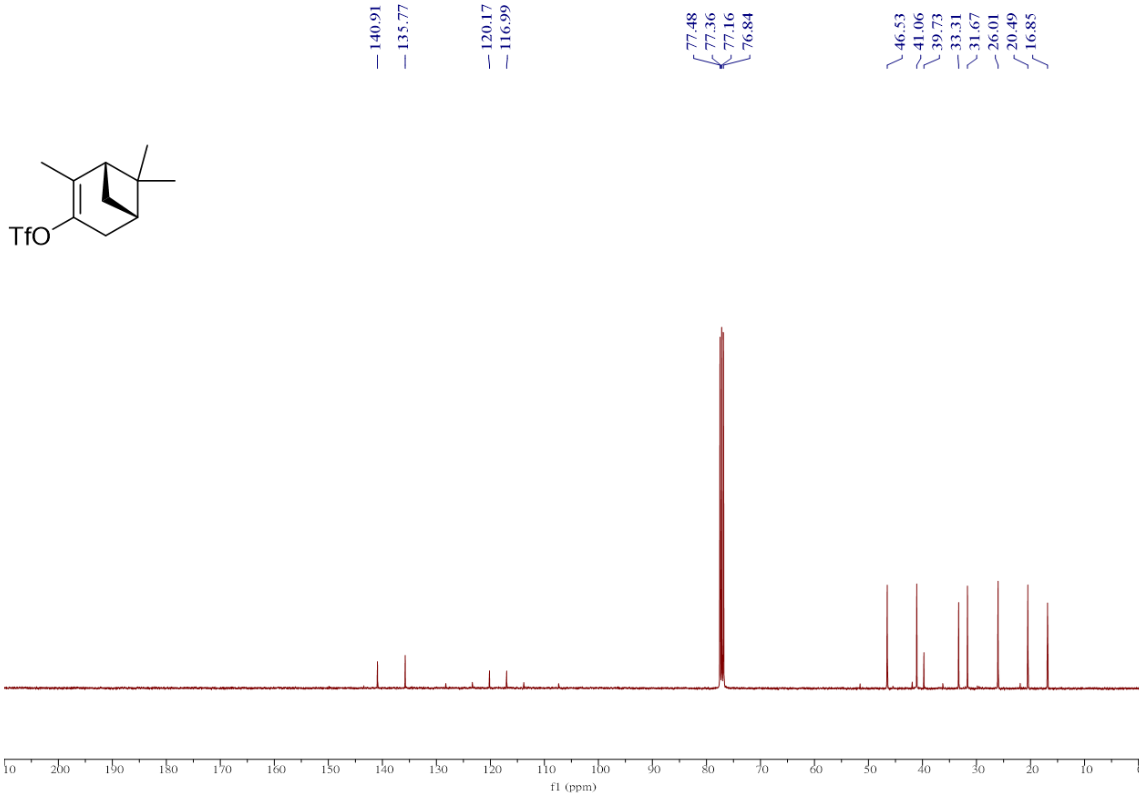

OTf-tPin (11) ^{19}F NMR, $CDCl_3$

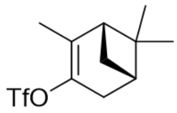

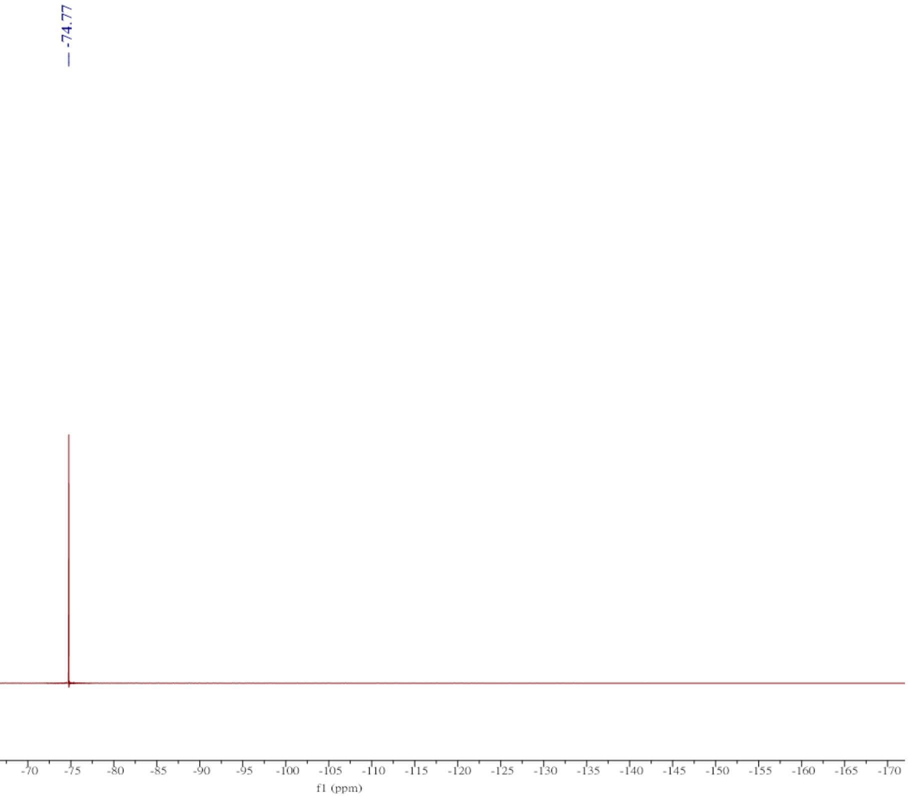

oNB-tPin (12) ^{1}H NMR, $CDCl_3$

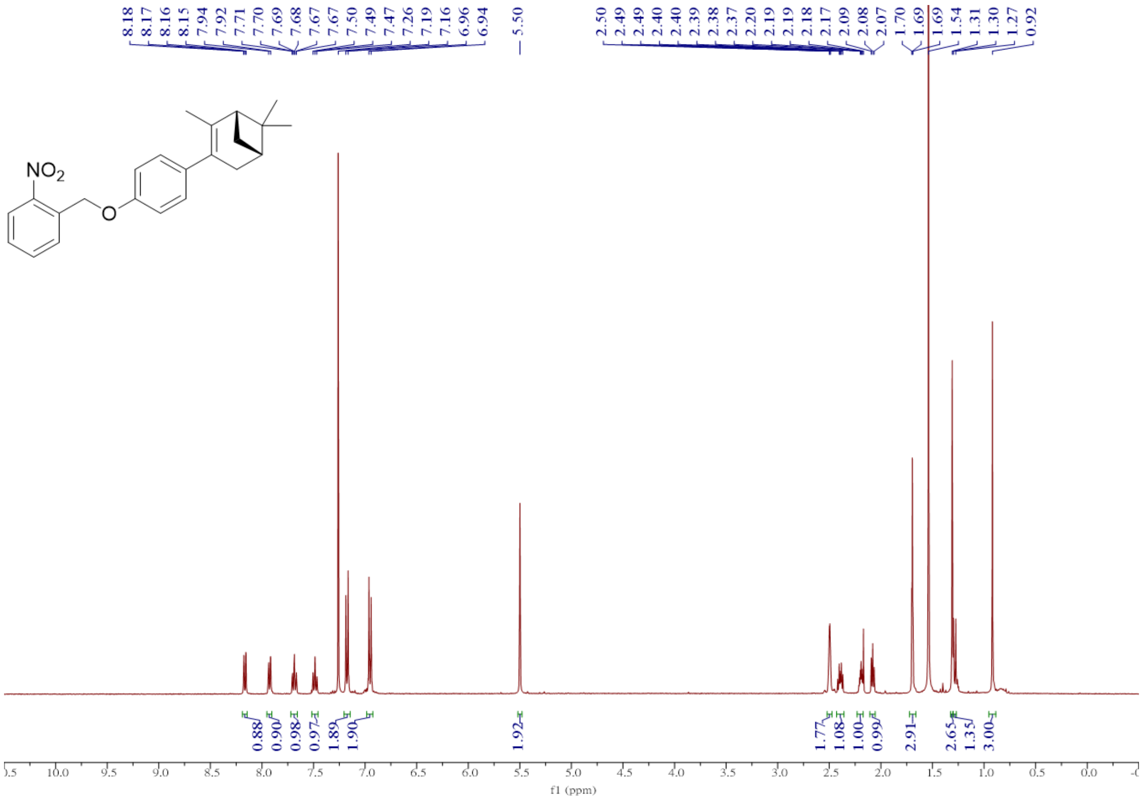

oNB-tPin (12) ^{13}C NMR, $CDCl_3$

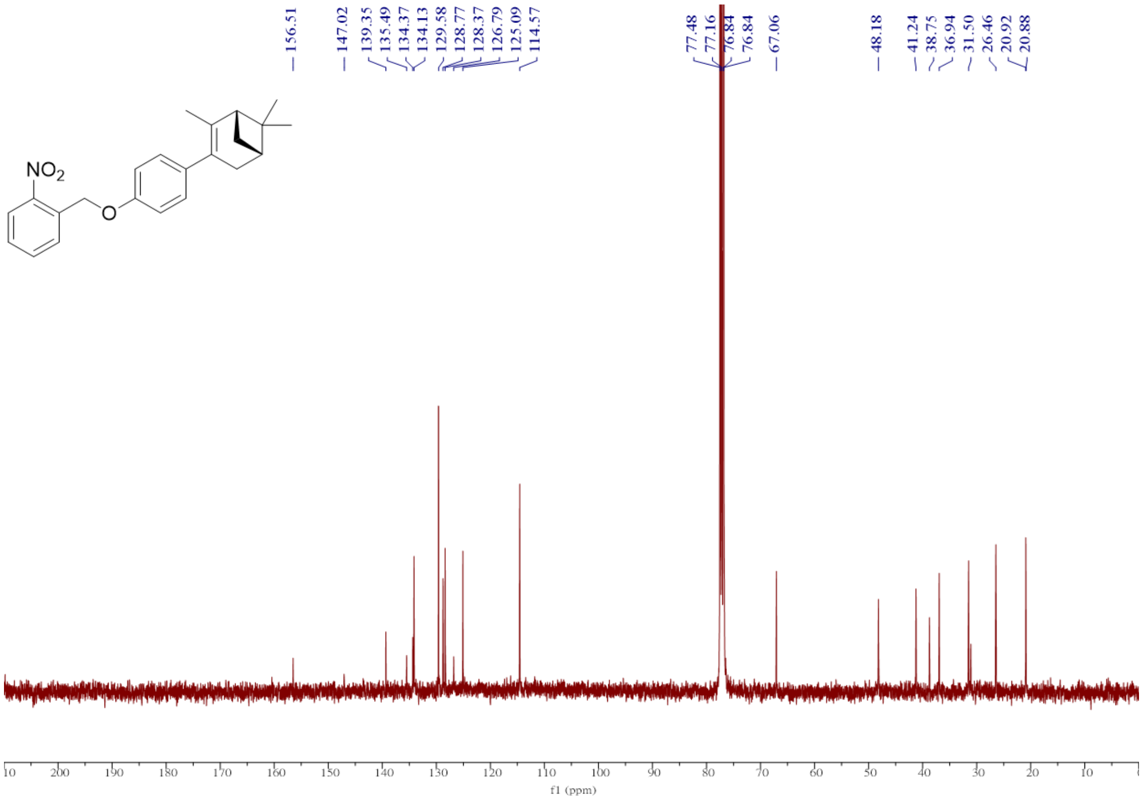

oNB-tPin-diol (13) ^{1}H NMR, $CDCl_3$

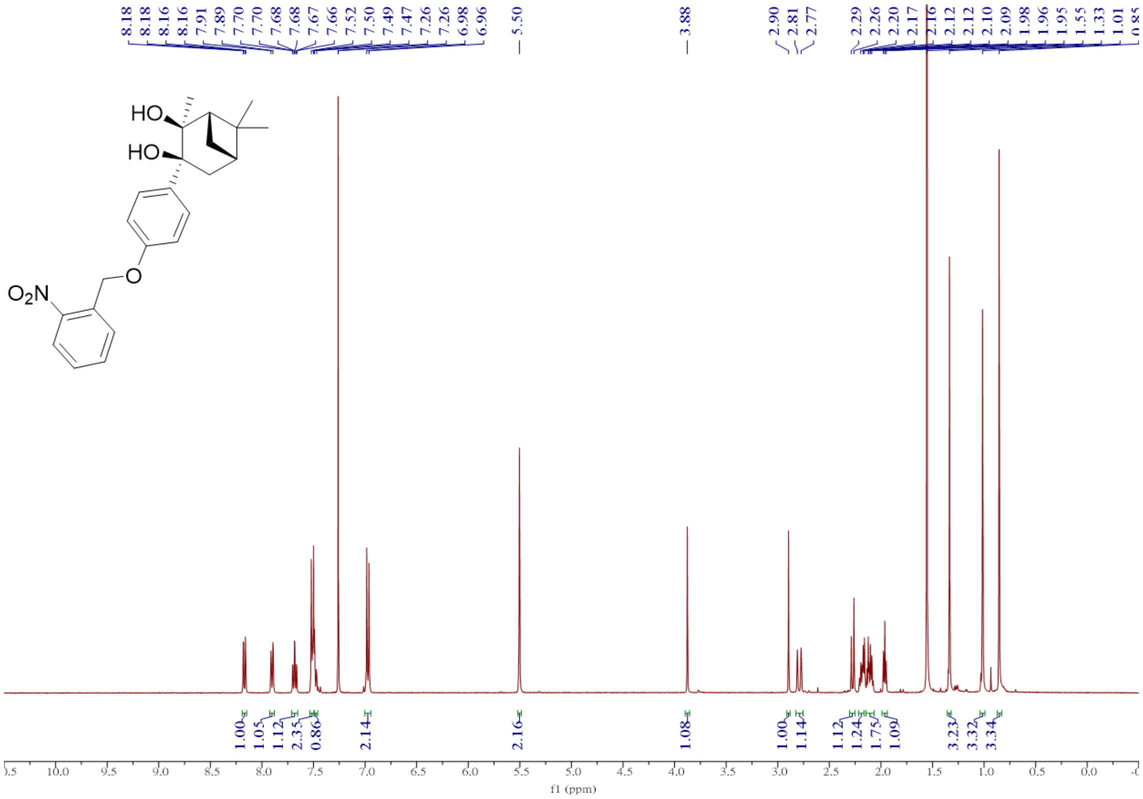

oNB-tPin-diol (13) ^{13}C NMR, $CDCl_3$

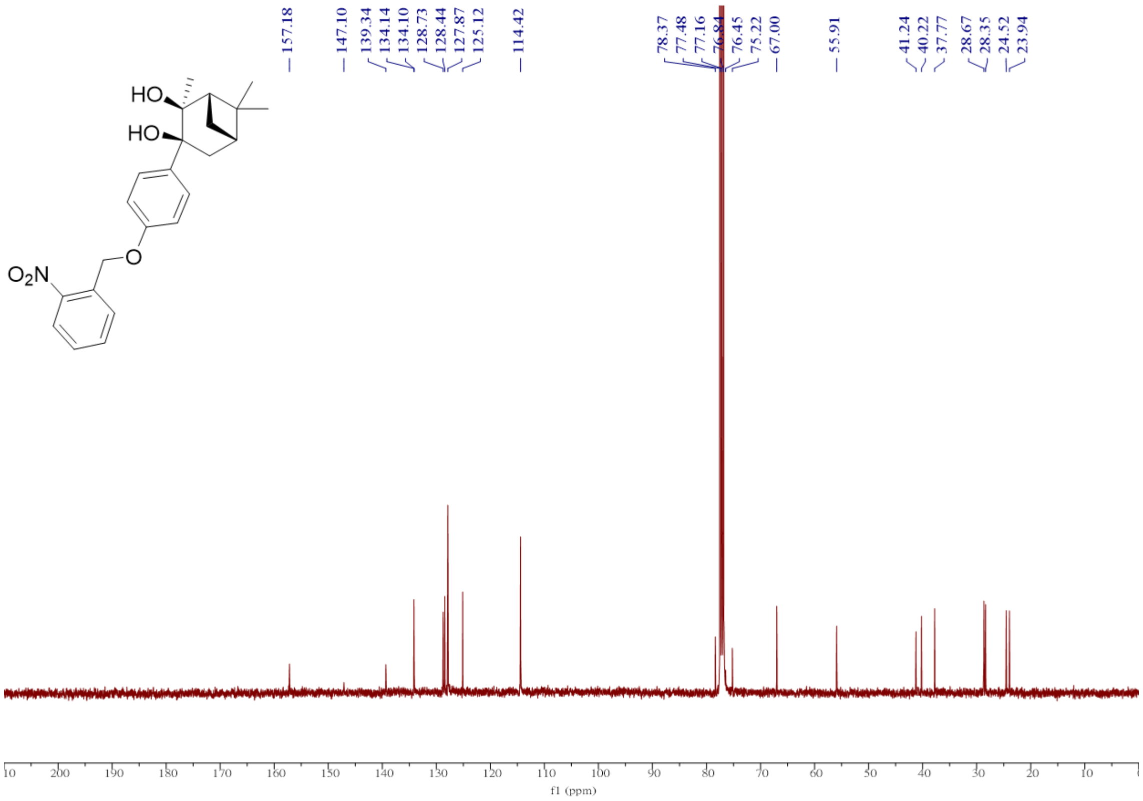

OTf-kPin (14) ^{1}H NMR, $CDCl_3$

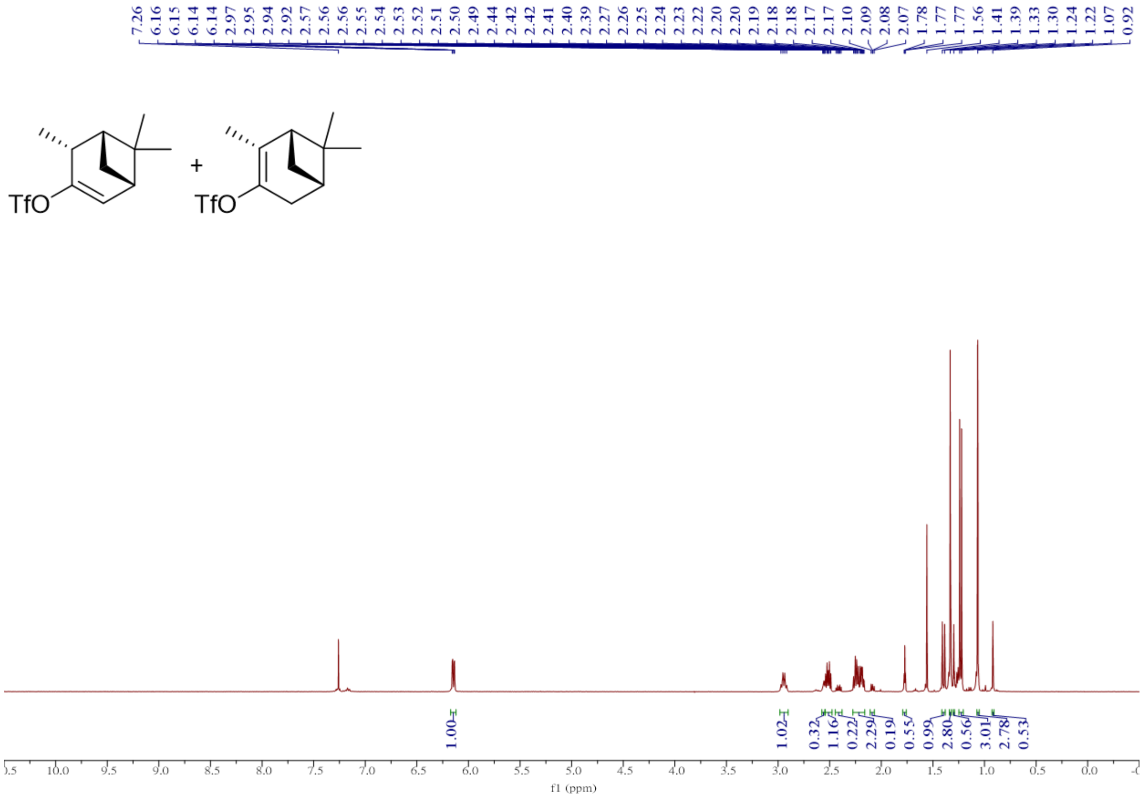

OTf-kPin (14) ^{13}C NMR, $CDCl_3$

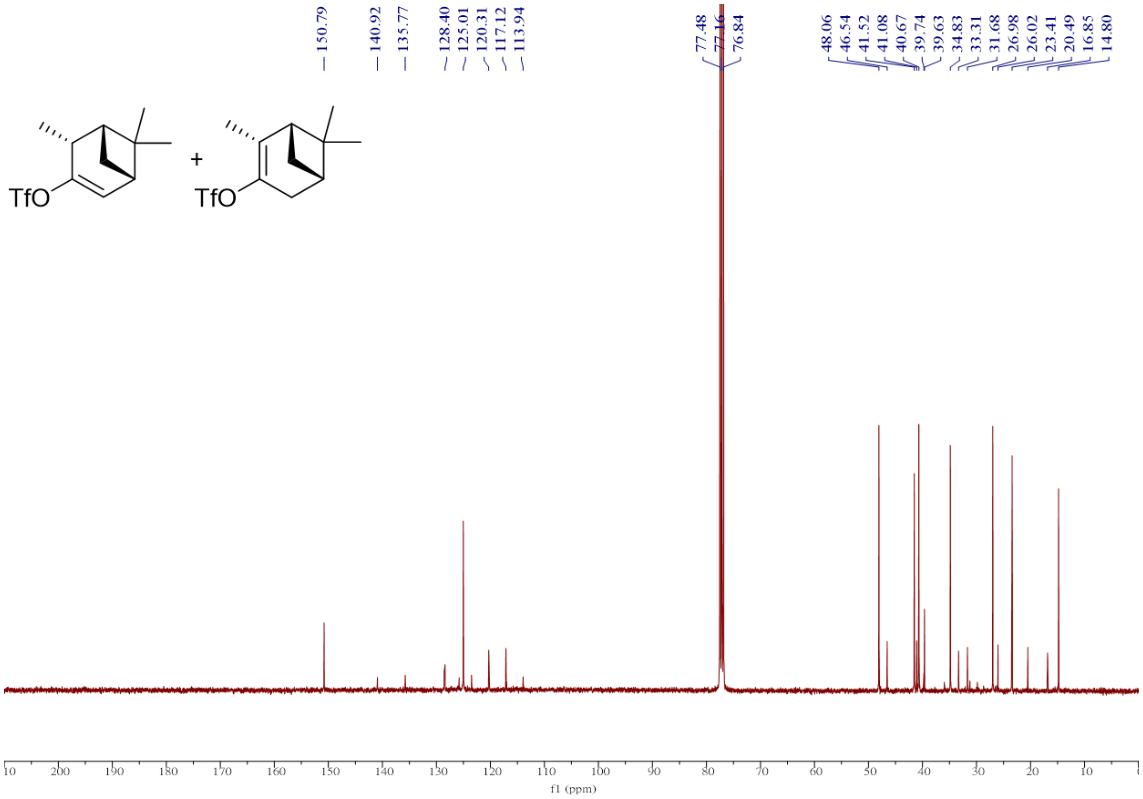

OTf-kPin (14) ^{19}F NMR, $CDCl_3$

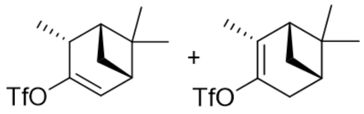

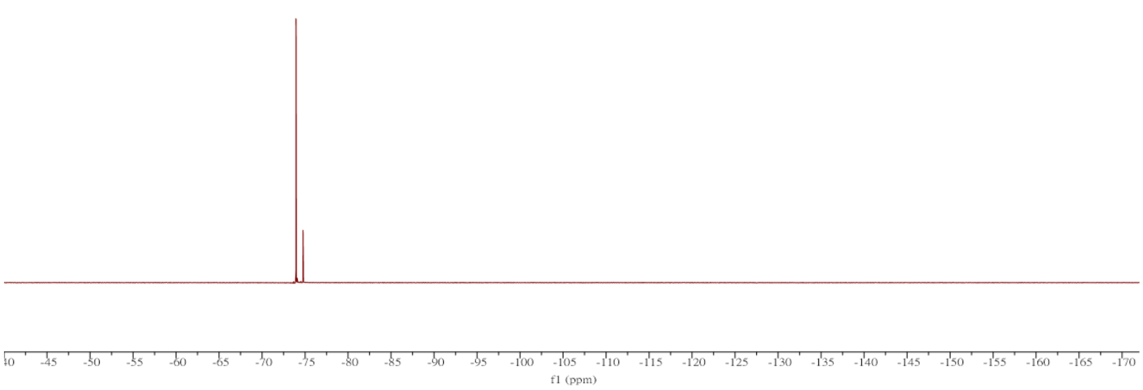

oNB-kPin (15) ^{1}H NMR, $CDCl_3$

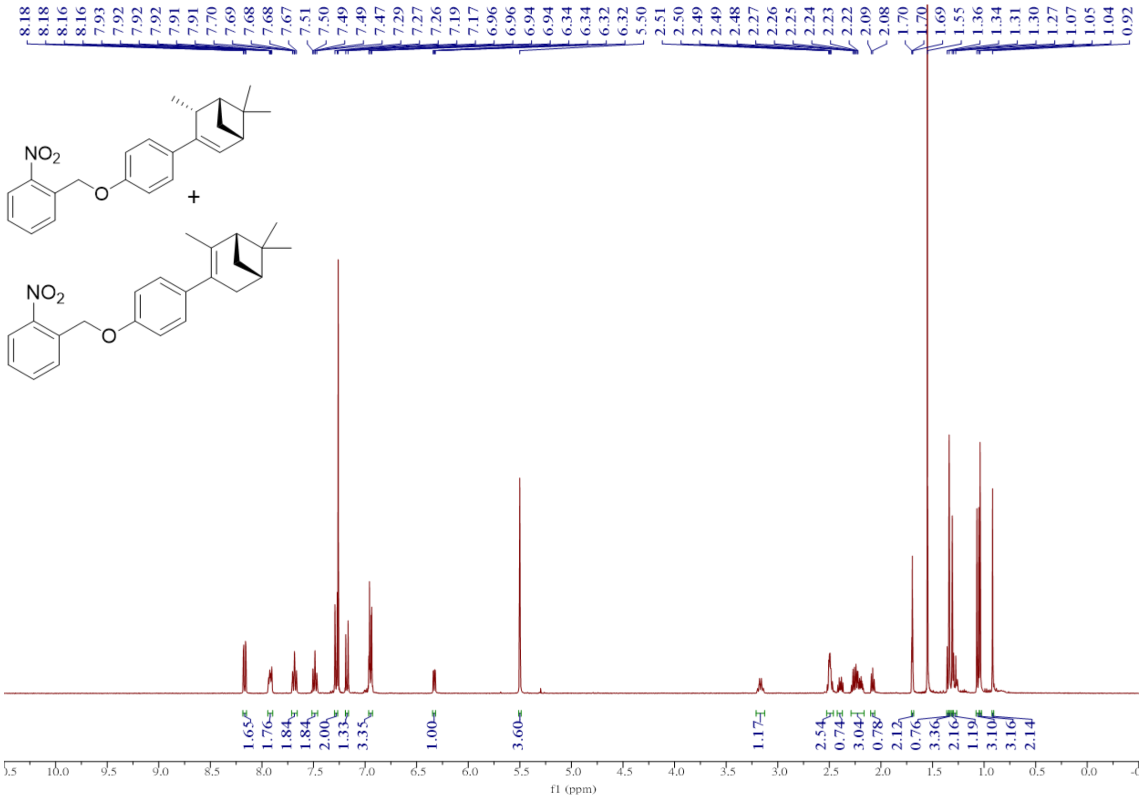

oNB-kPin (15) ^{13}C NMR, $CDCl_3$

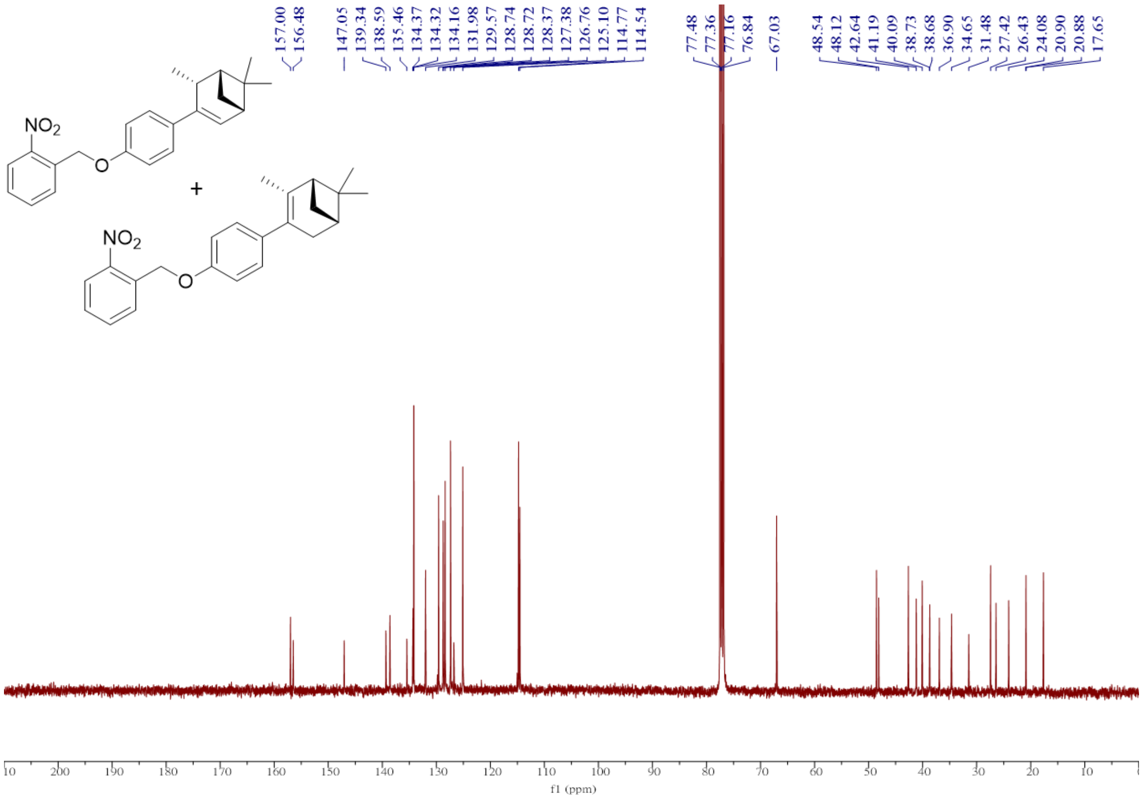

oNB-kPin-diol (16) ^{1}H NMR, CDCl$_3$

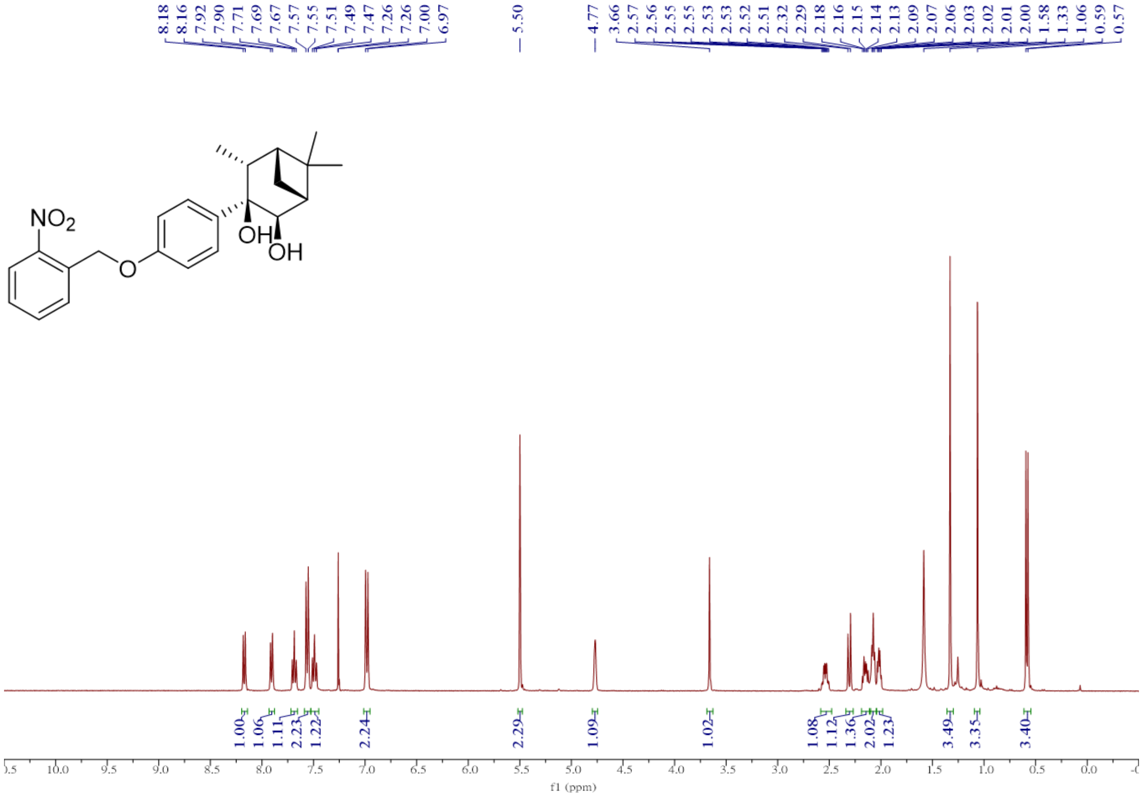

oNB-kPin-diol (16) ^{13}C NMR, CDCl$_3$

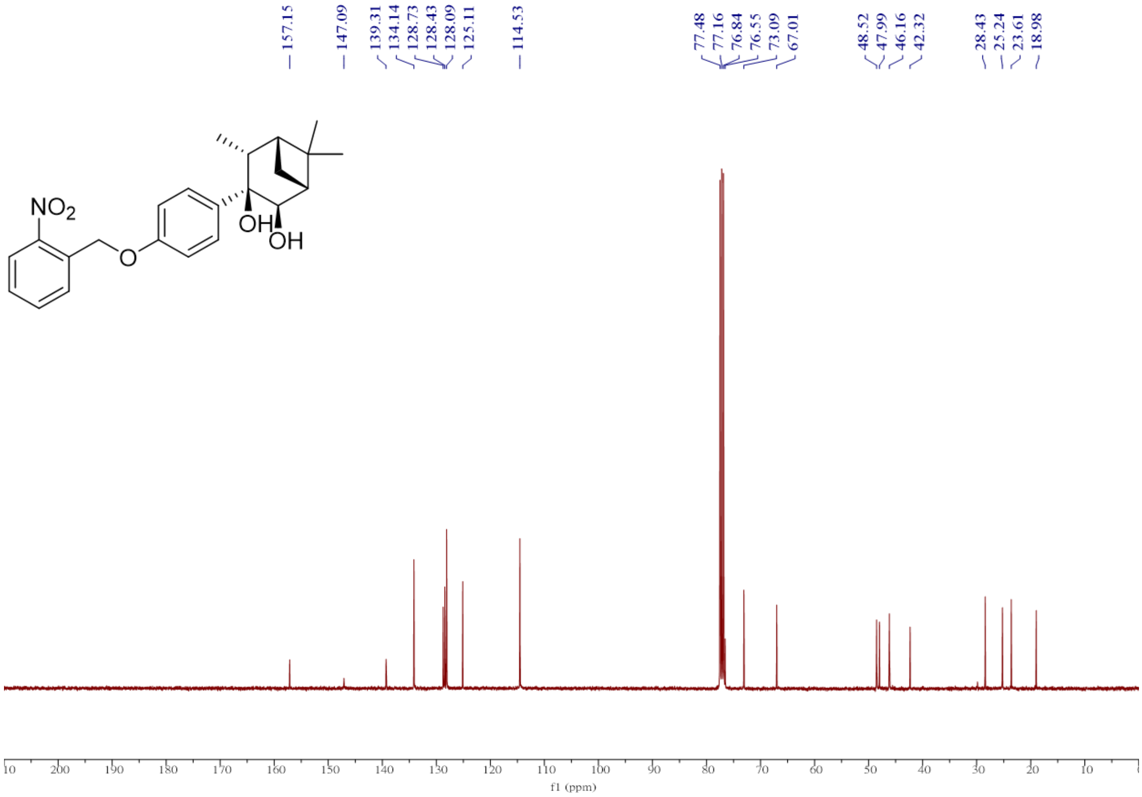

oNB-kPin-EtPh (17) ^{1}H NMR, $CDCl_3$

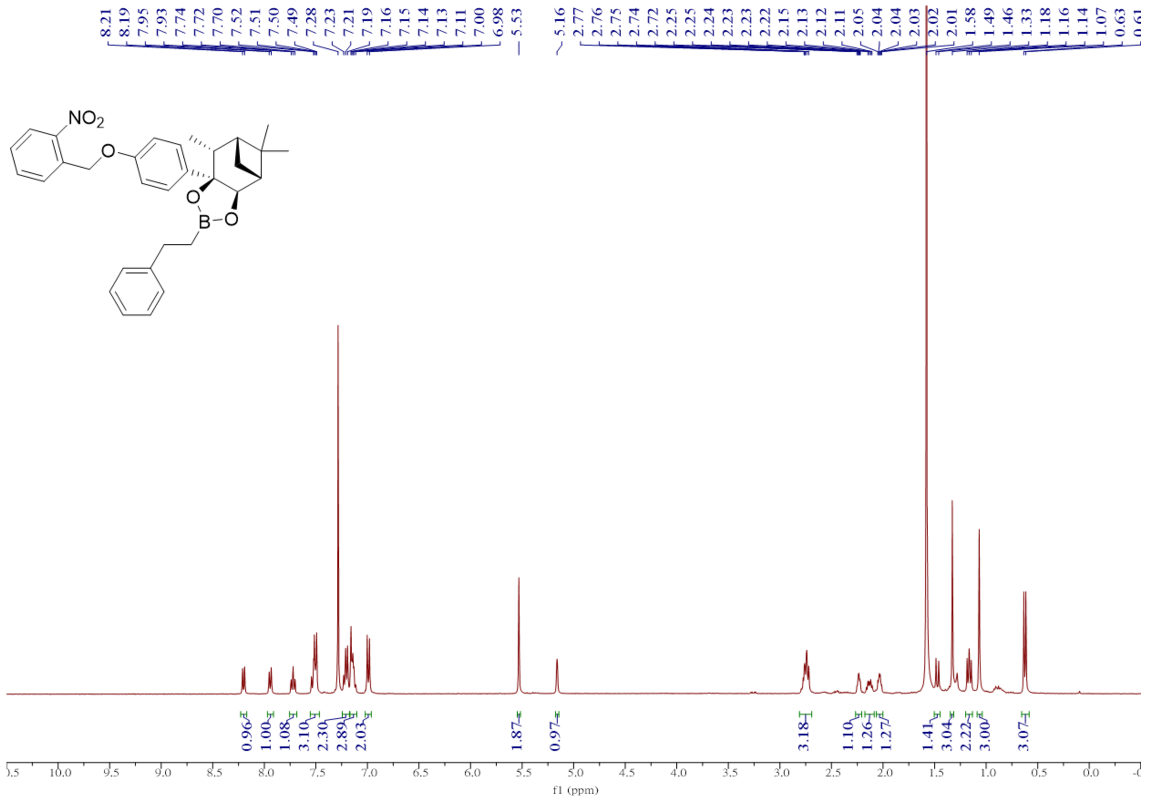

oNB-kPin-EtPh (17) ^{13}C NMR, $CDCl_3$

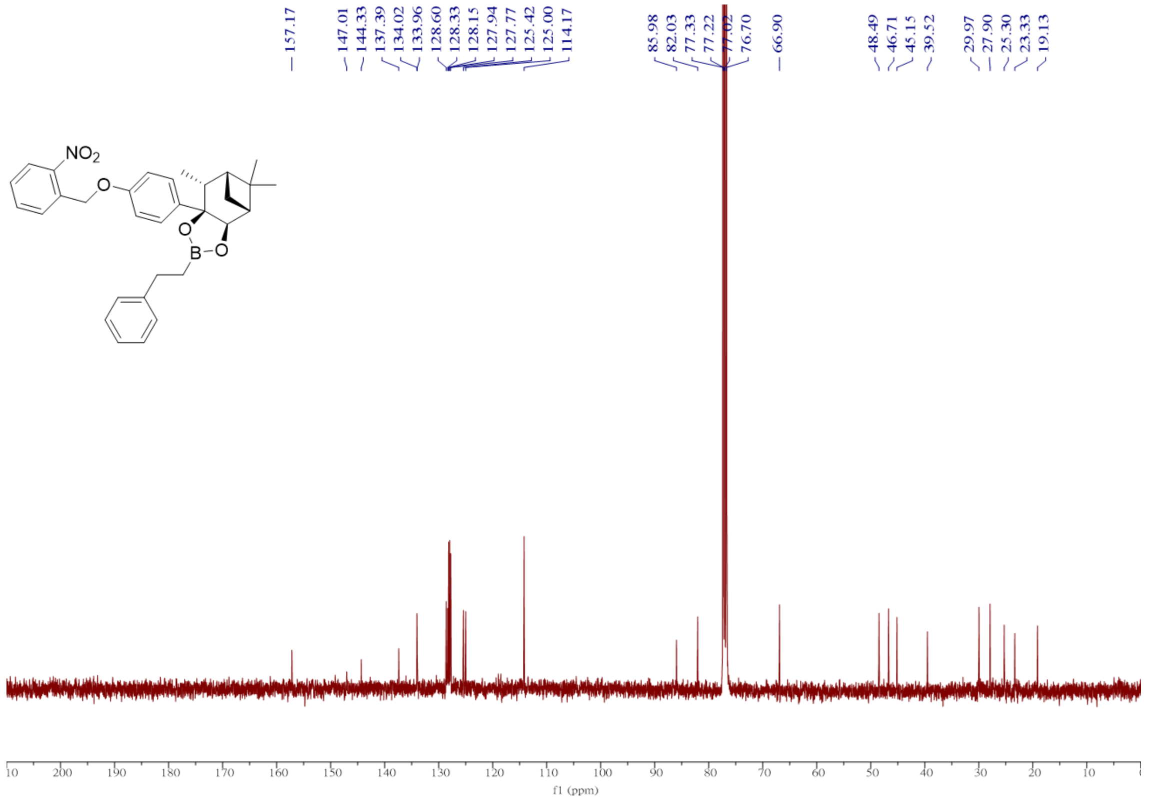

Ph-kPin (19) ^{1}H NMR, $CDCl_3$

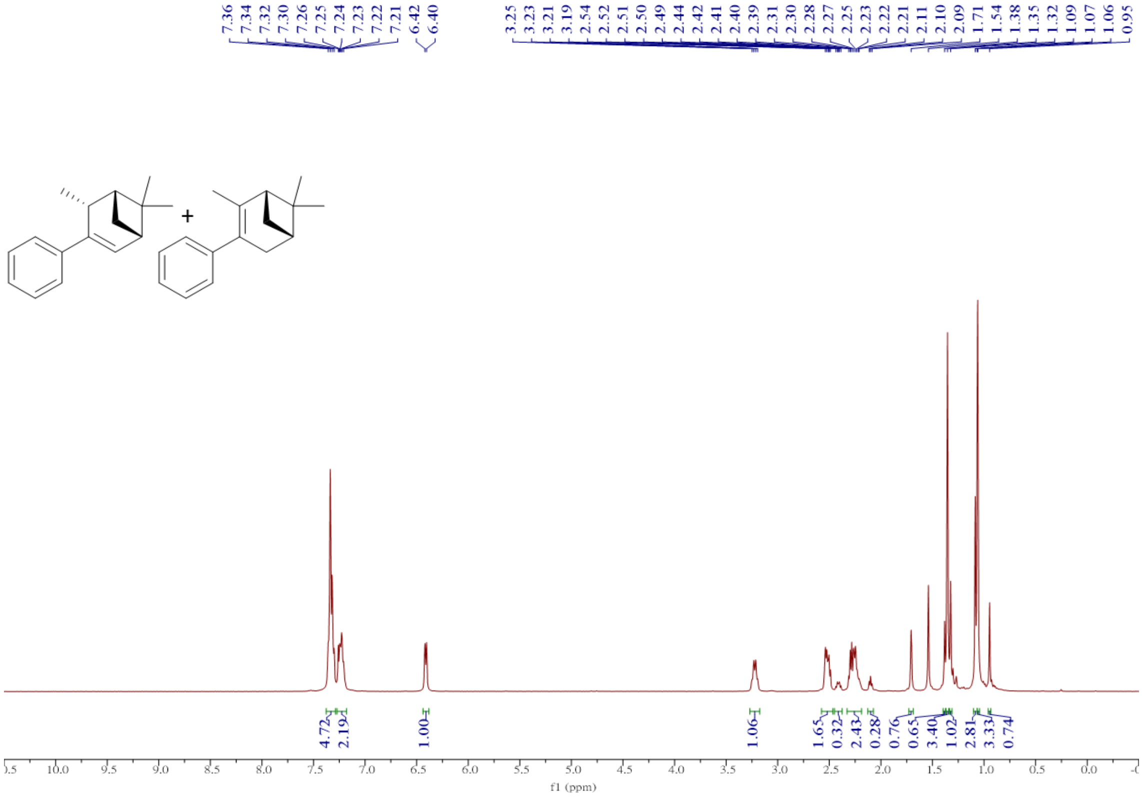

Ph-kPin (19) ^{13}C NMR, $CDCl_3$

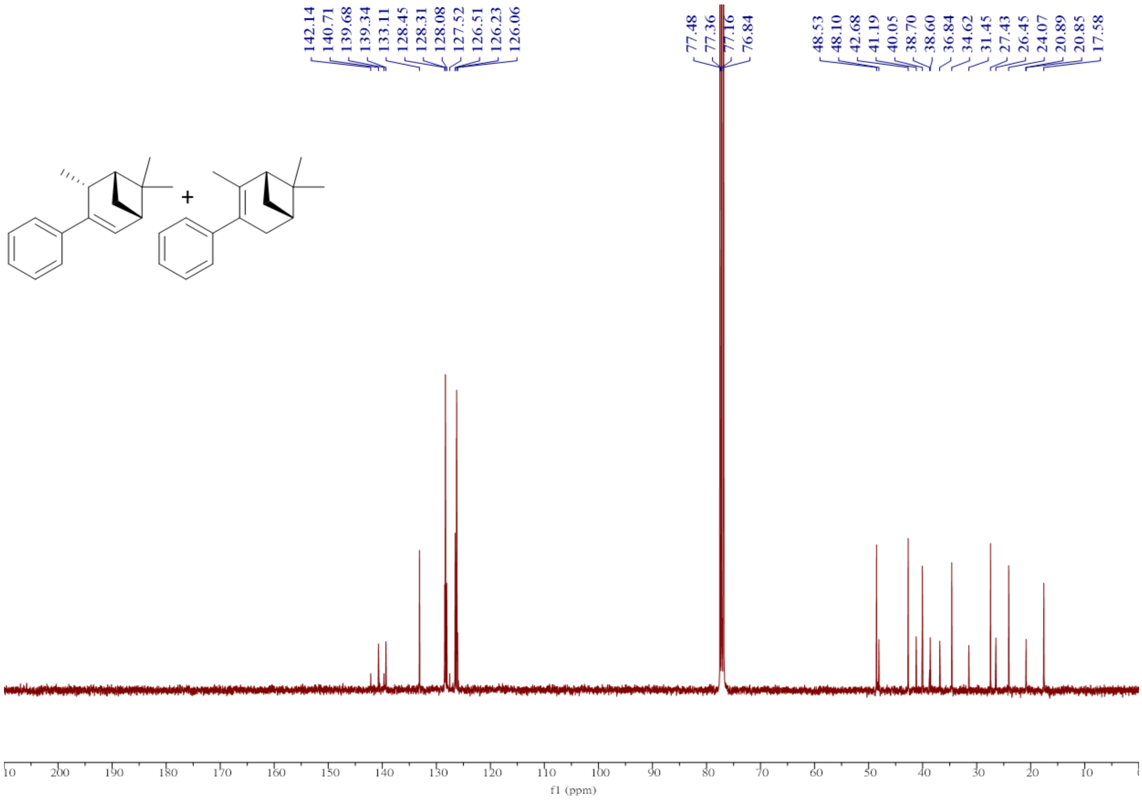

Ph-kPin-diol (21) ^{1}H NMR, $CDCl_3$

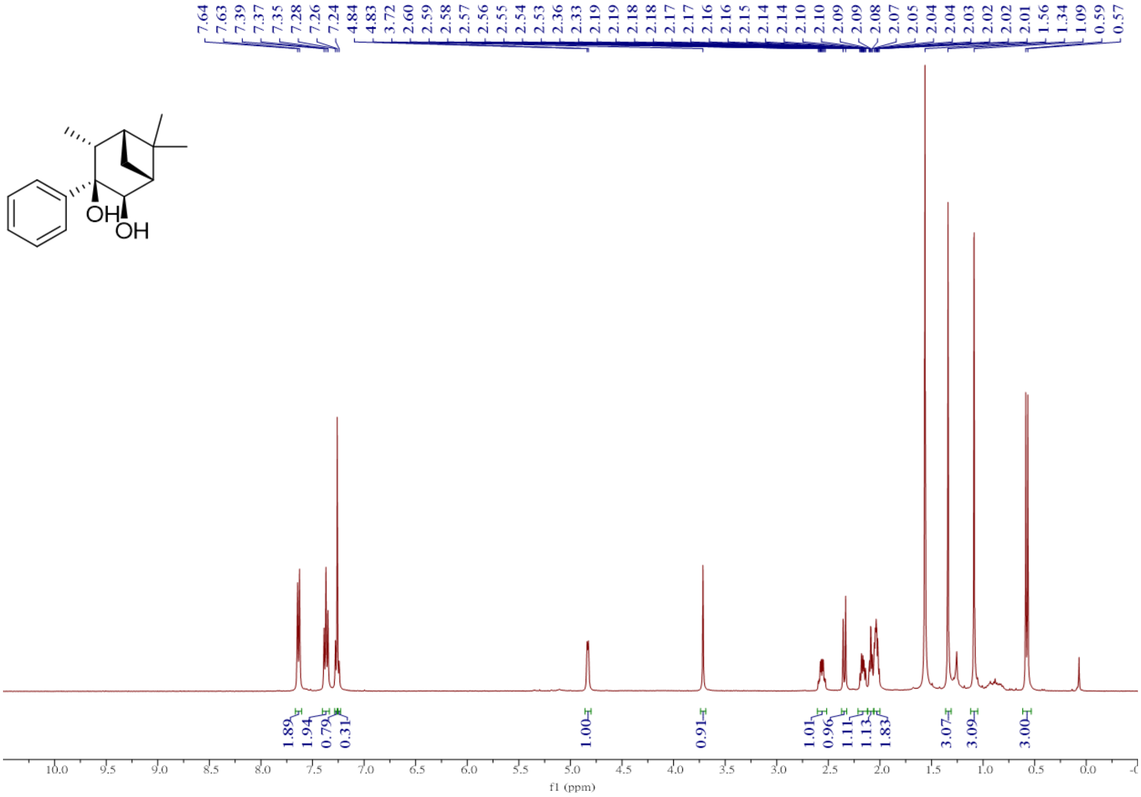

Ph-kPin-diol (21) ^{13}C NMR, $CDCl_3$

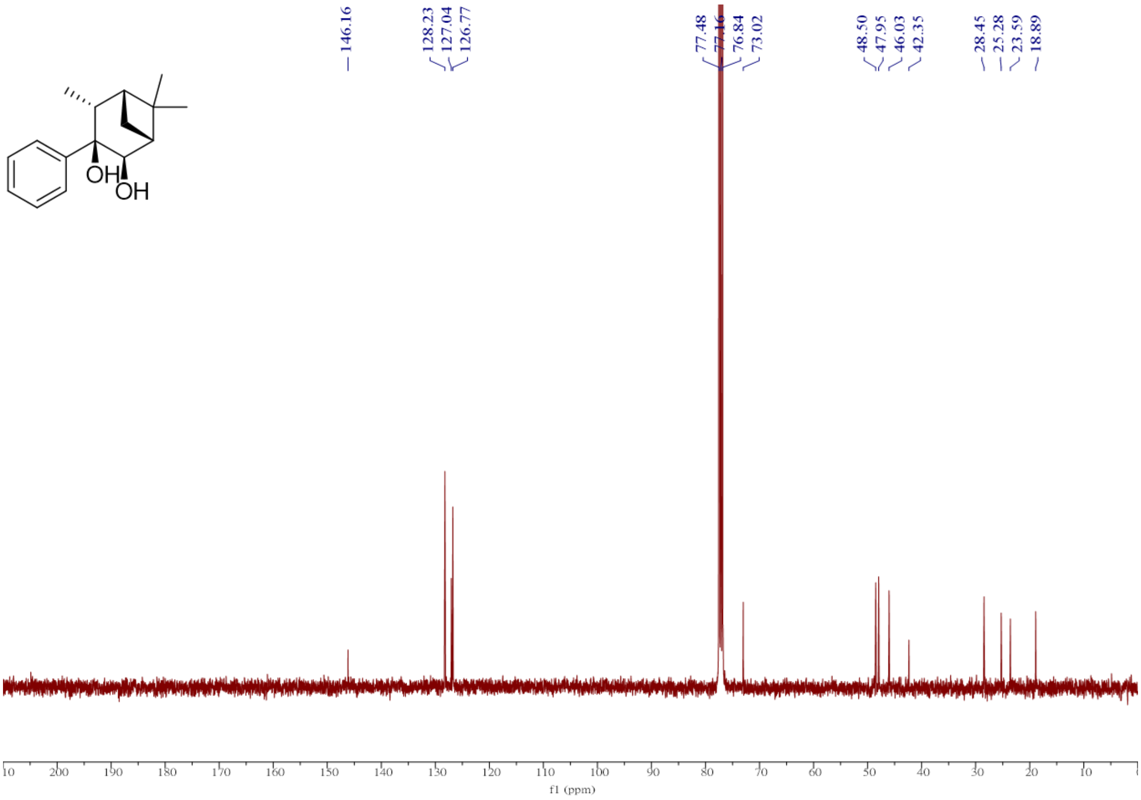

NP-kPin (22) ^{1}H NMR, $CDCl_3$

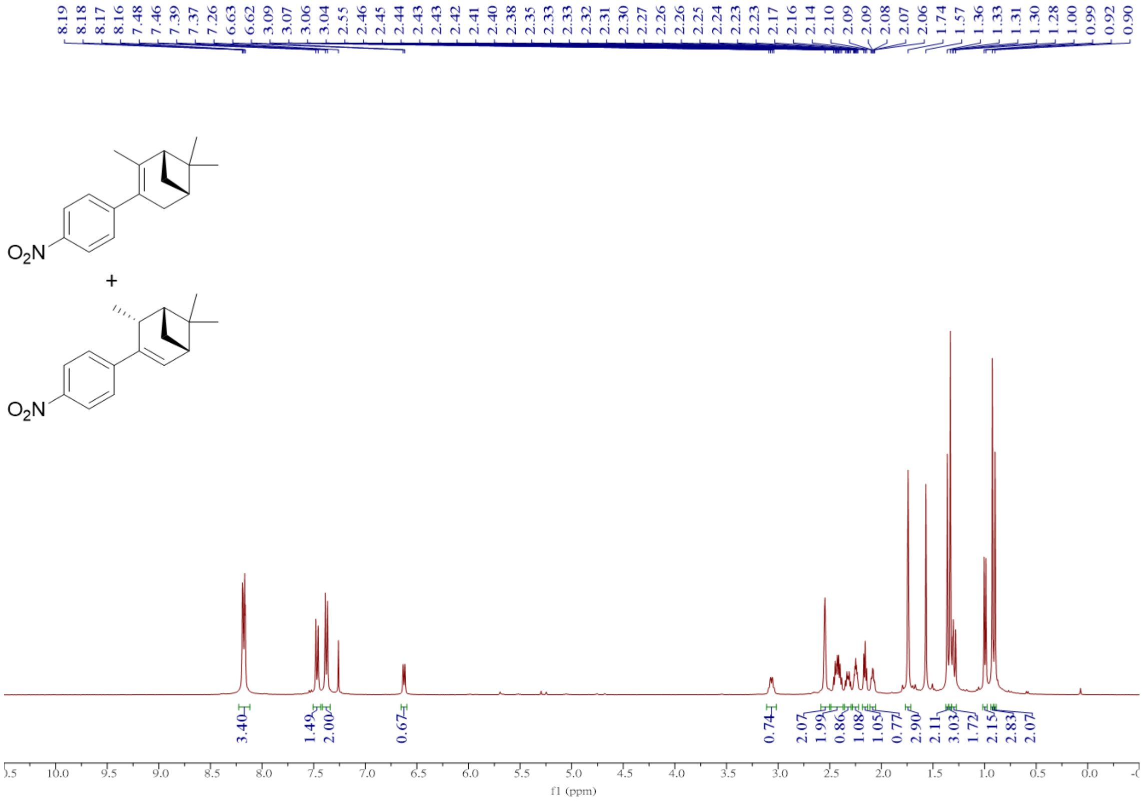

NP-kPin (22) ^{13}C NMR, $CDCl_3$

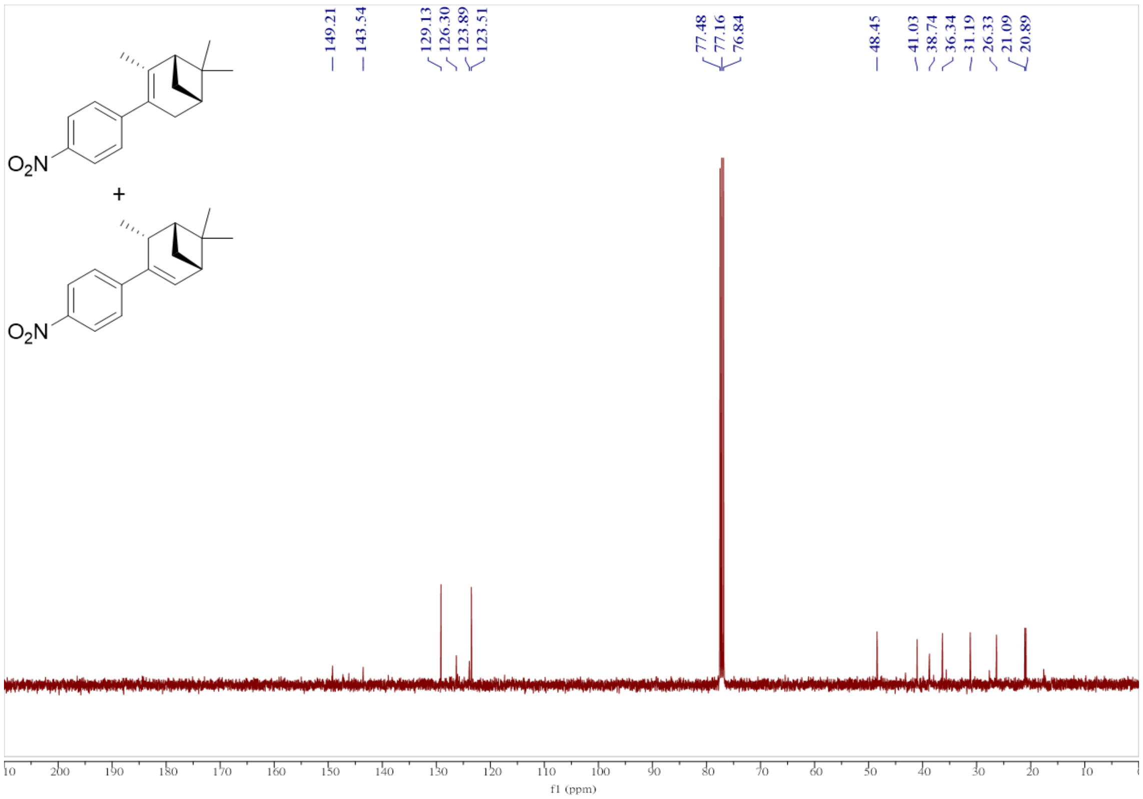

NP-kPin-diol (24) ^{1}H NMR, $CDCl_3$

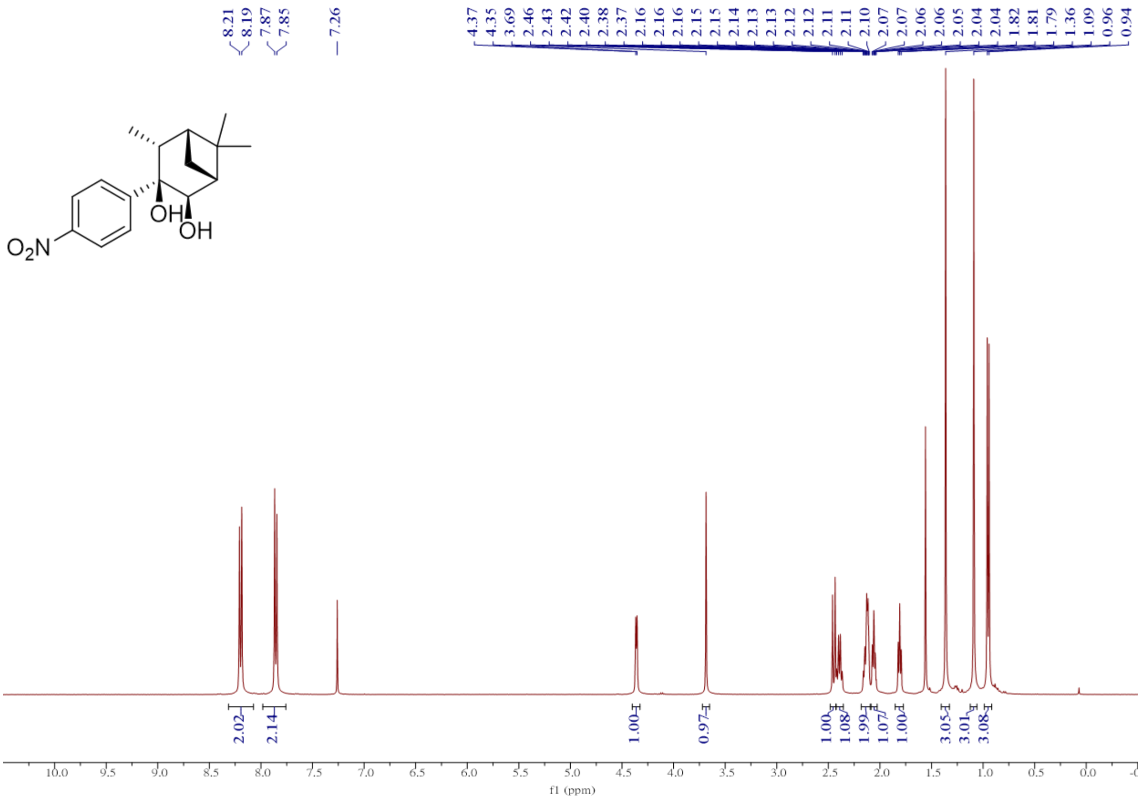

NP-kPin-diol (24) ^{13}C NMR, $CDCl_3$

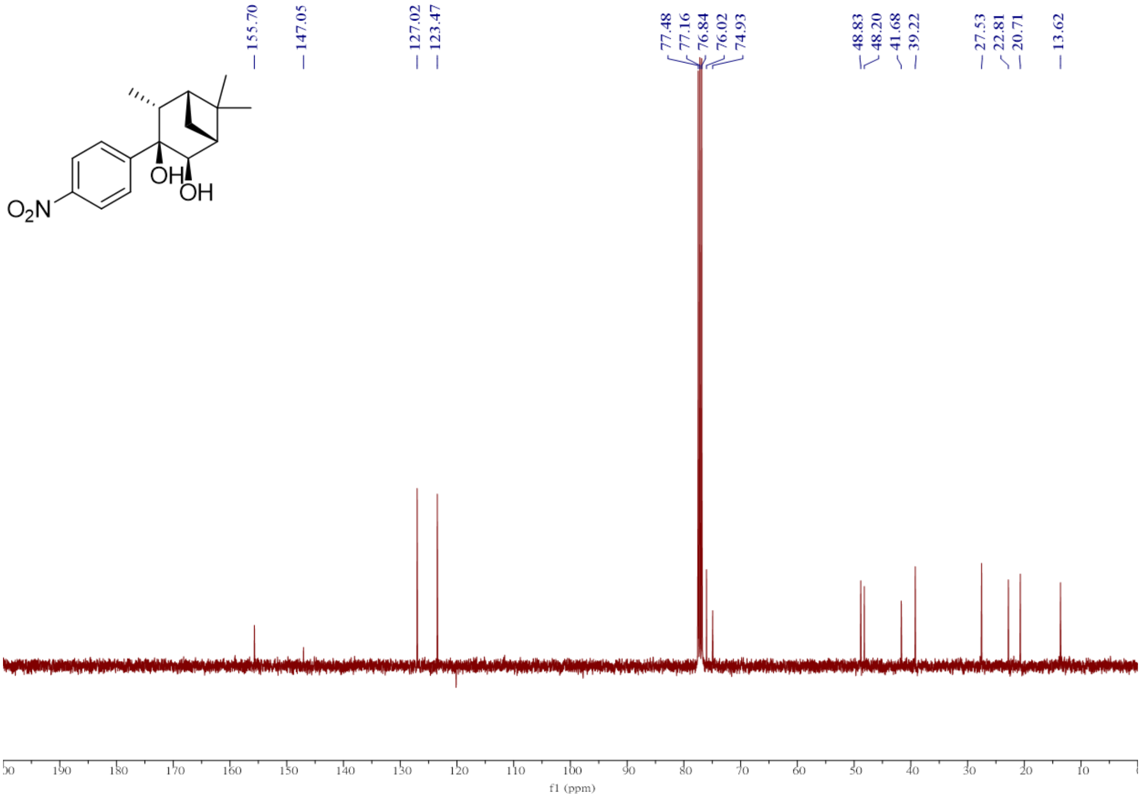

pNB-Br (25) ^{1}H NMR, $CDCl_3$

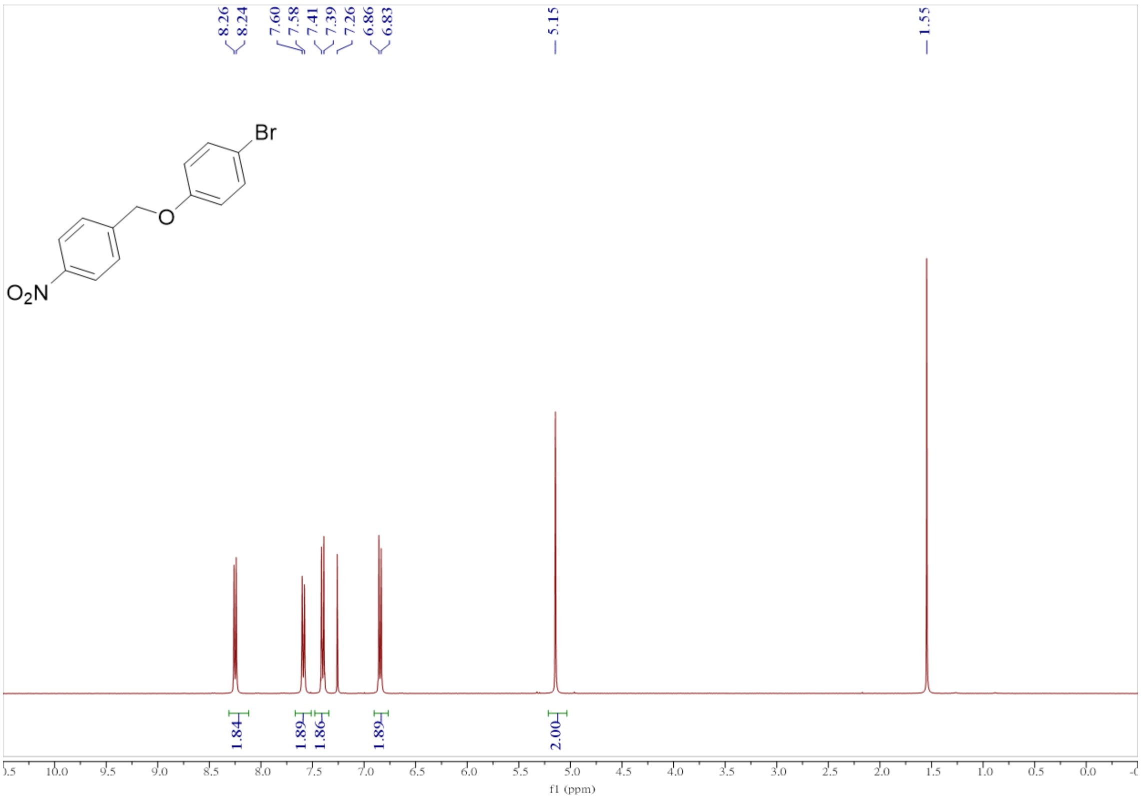

pNB-Br (25) ^{13}C NMR, $CDCl_3$

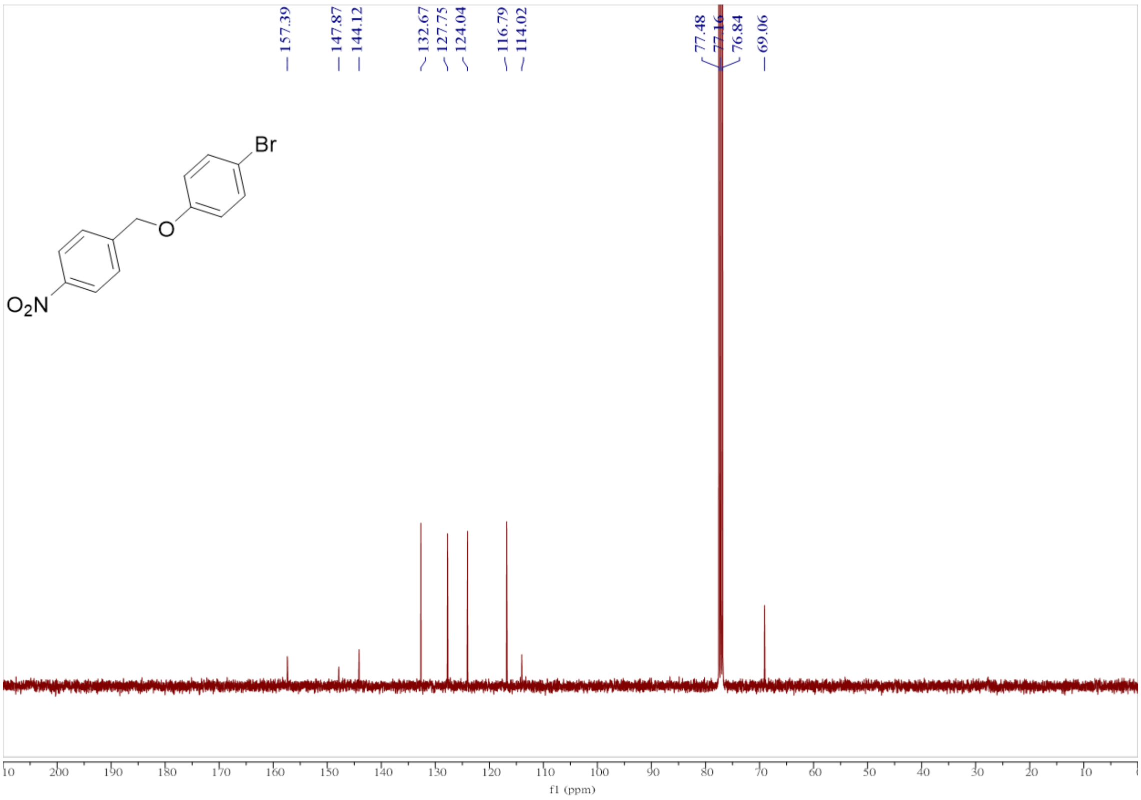

pNB-Pinacol (26) ^{1}H NMR, $CDCl_3$

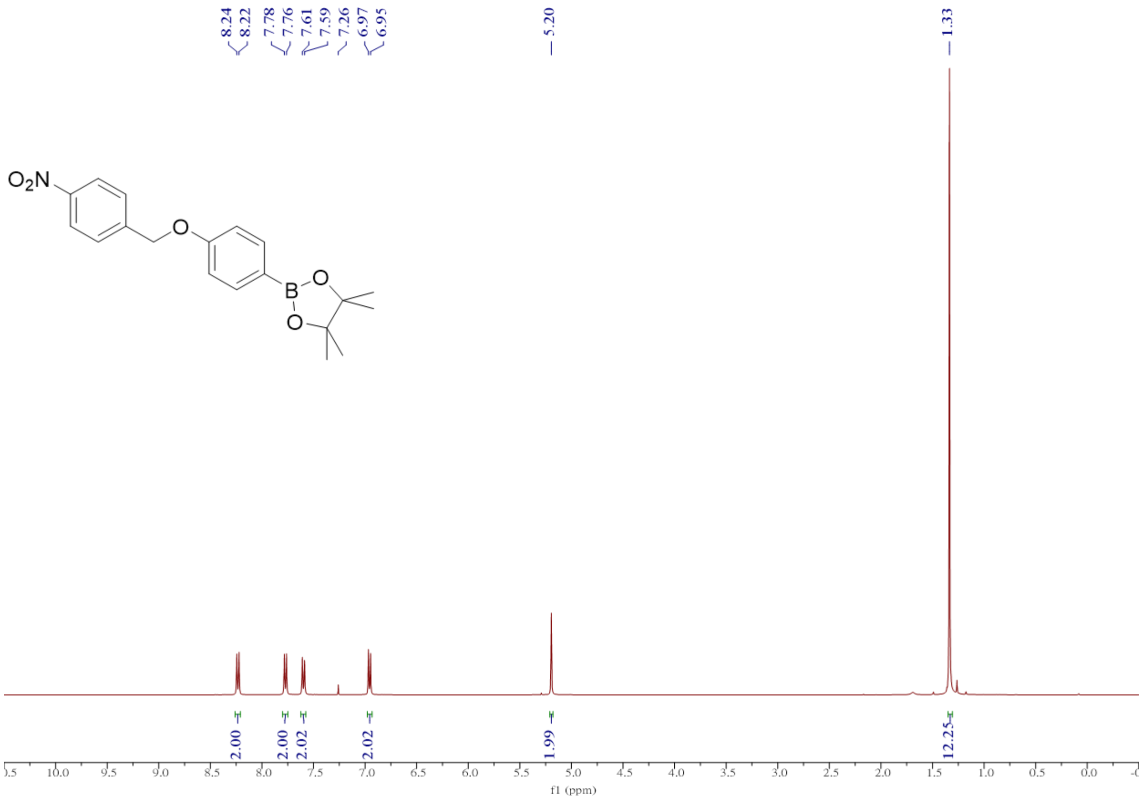

pNB-Pinacol (26) ^{13}C NMR, $CDCl_3$

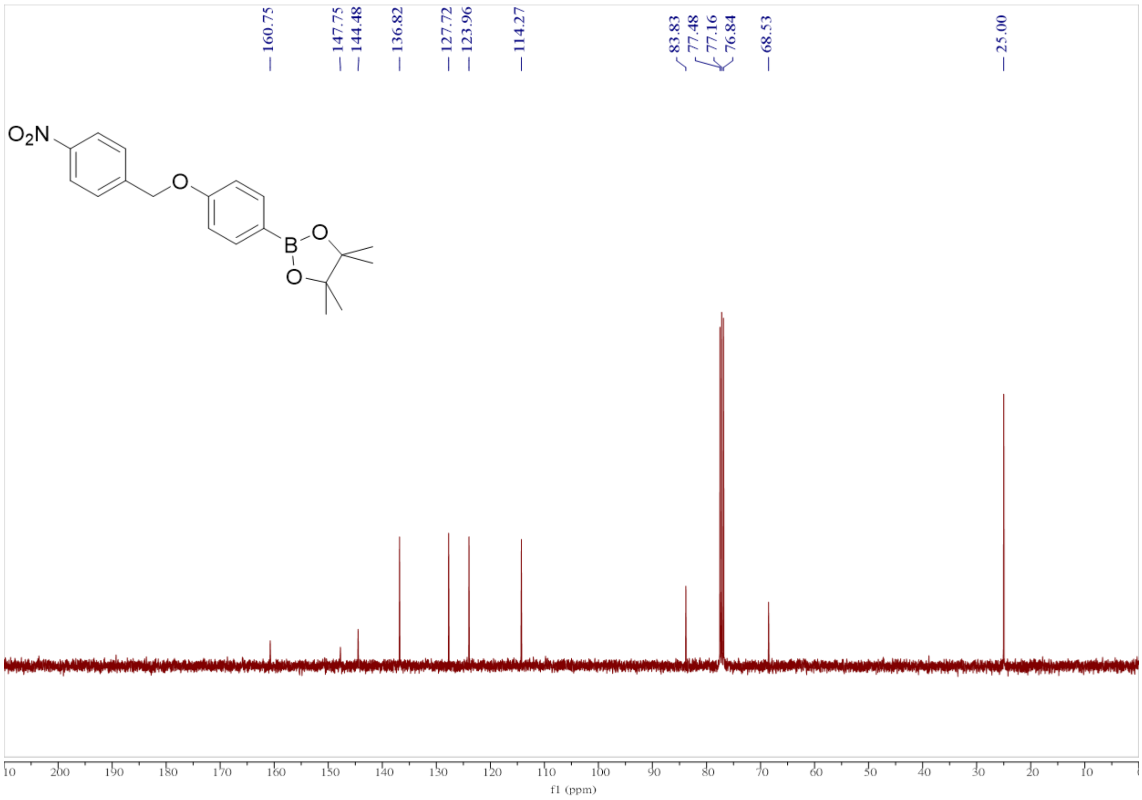

pNB-kPin (27) ^{1}H NMR, $CDCl_3$

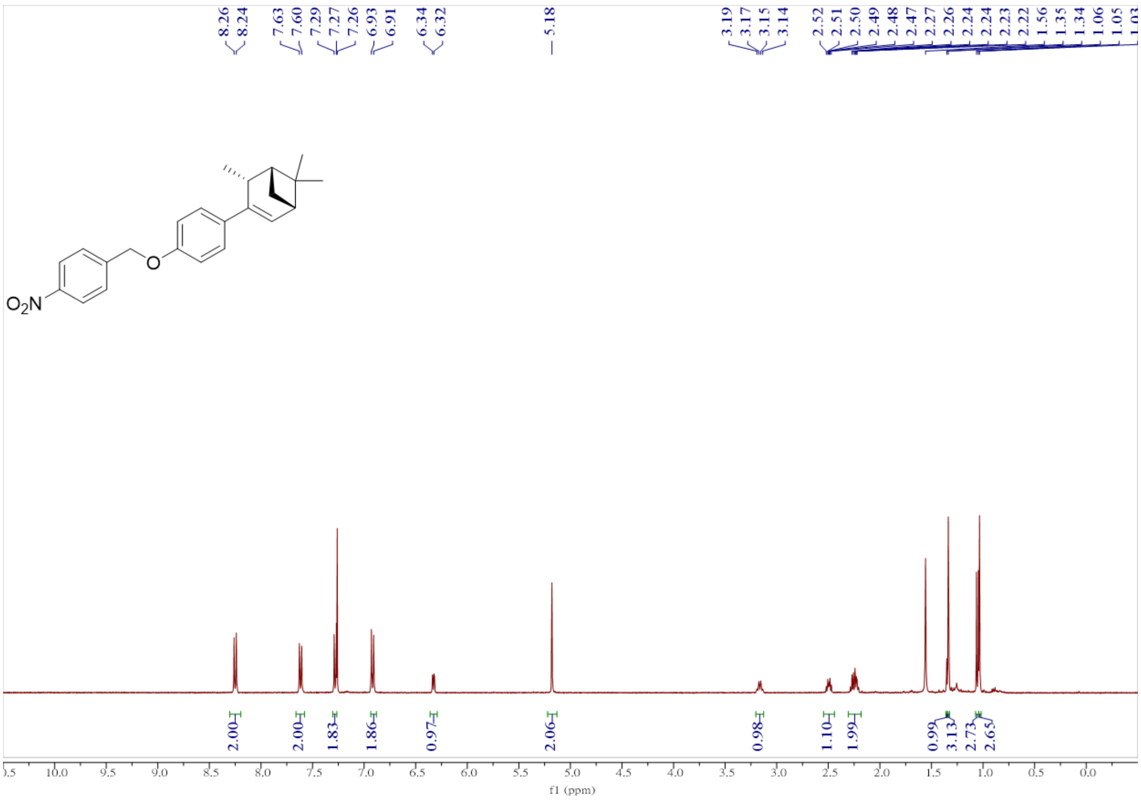

pNB-kPin (27) ^{13}C NMR, $CDCl_3$

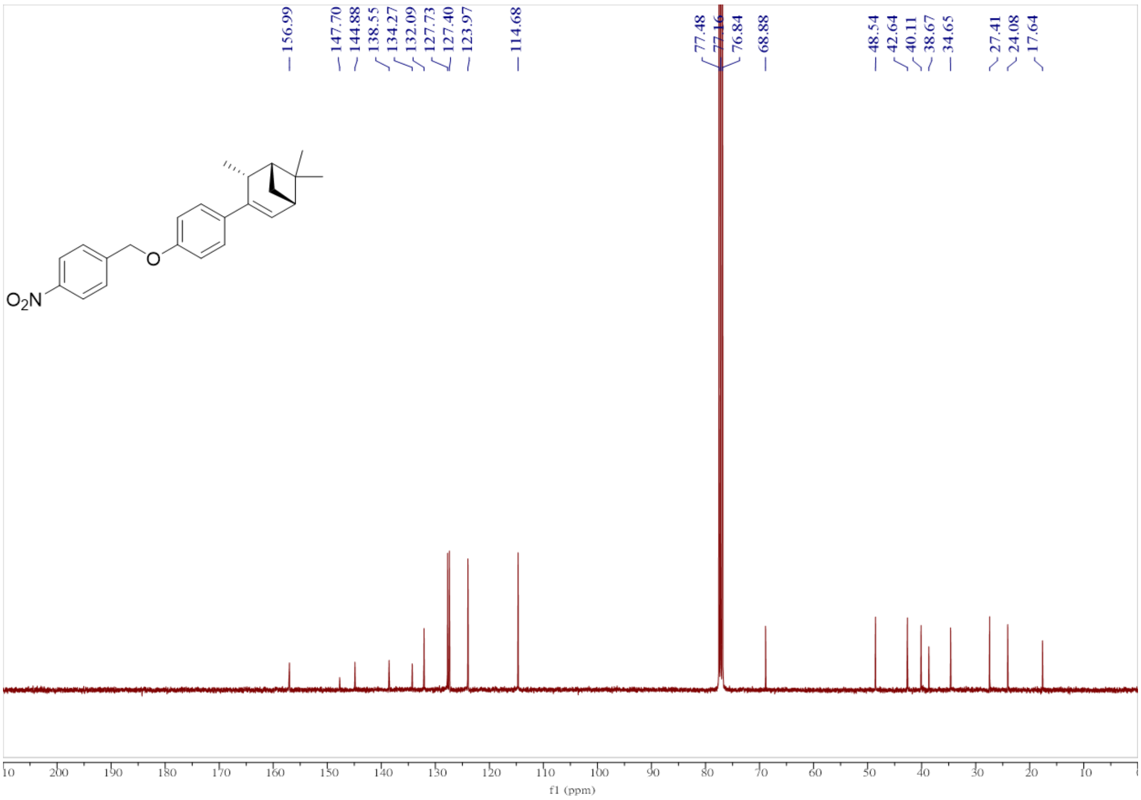

pNB-kPin-diol (28) ^{1}H NMR, $CDCl_3$

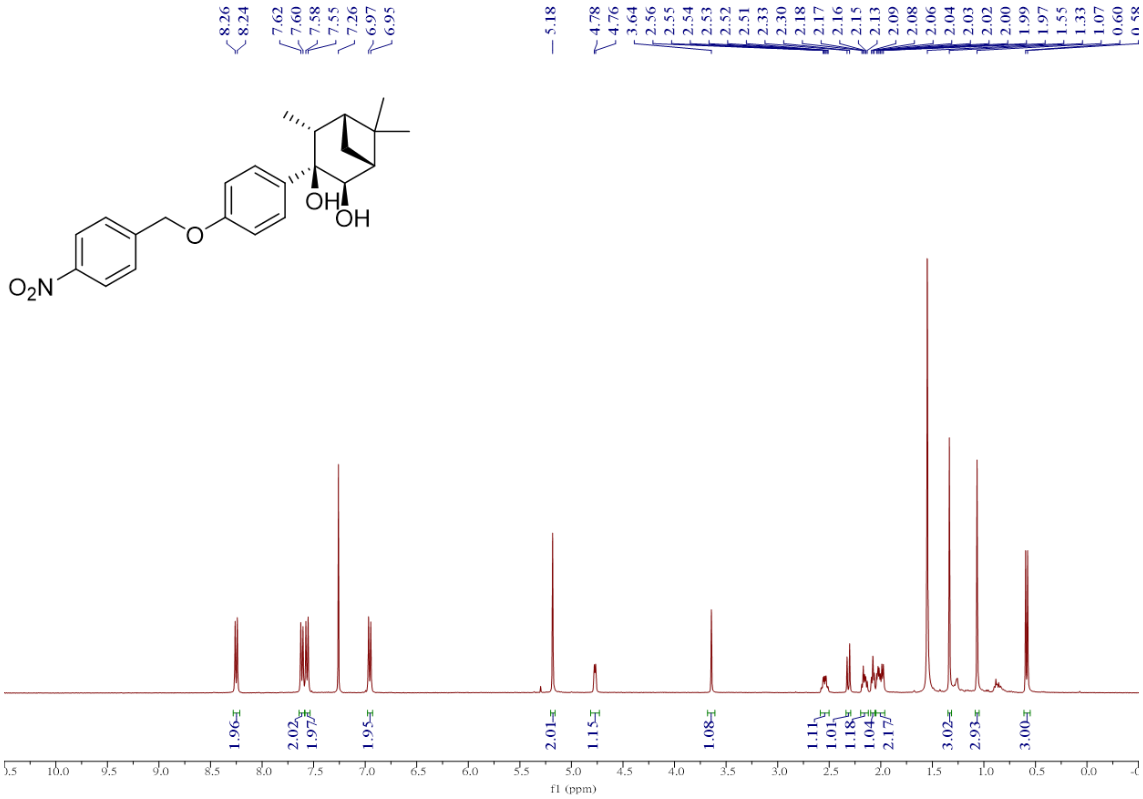

pNB-kPin-diol (28) ^{13}C NMR, $CDCl_3$

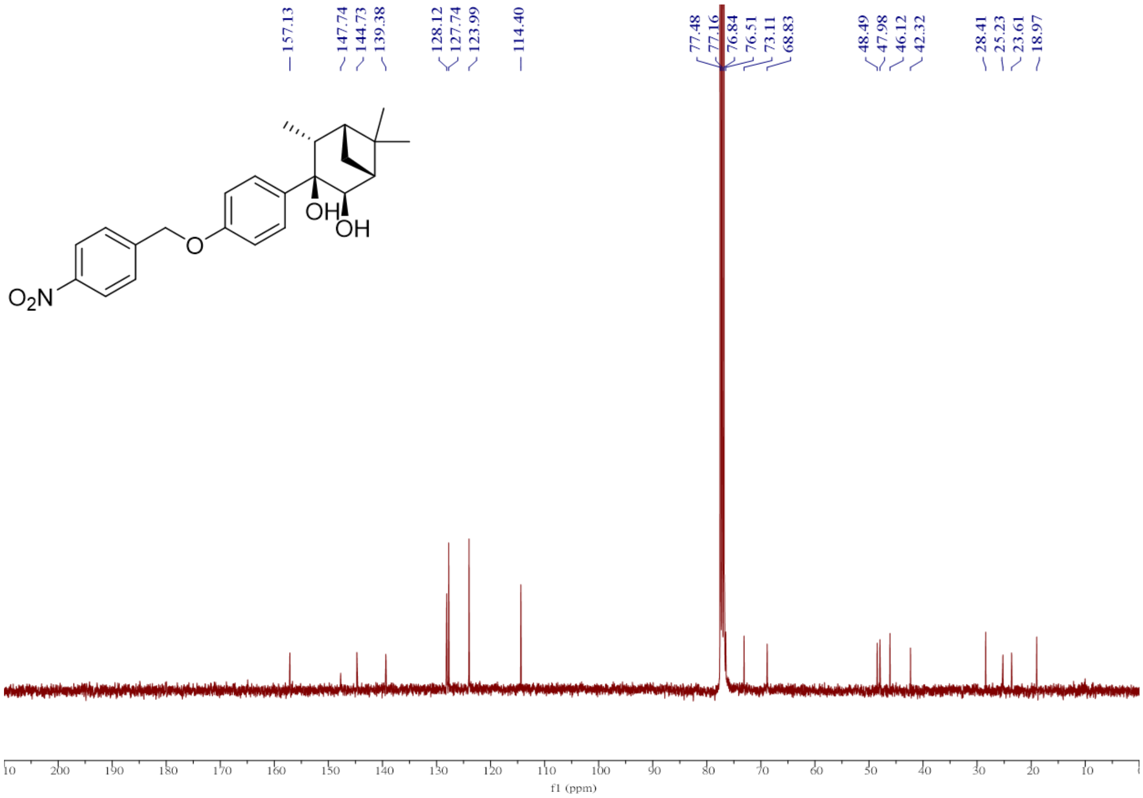

8. References

[1] N. P. Michael, J. K. Brehm, G. M. Anlezark, N. P. Minton, Physical characterisation of the Escherichia coli B gene encoding nitroreductase and its over-expression in Escherichia coli K12, *FEMS Microbiol. Lett.* **1994**, *124*(2), 195-202.

[2] G. M. Anlezark, R. G. Melton, R. F. Sherwood, B. Coles, F. Friedlos, R. J. Knox, The bioactivation of 5-(aziridin-1-yl)-2,4-dinitrobenzamide (CB1954)—I: Purification and properties of a nitroreductase enzyme from Escherichia coli—A potential enzyme for antibody-directed enzyme prodrug therapy (ADEPT), *Biochem. Pharmacol.* **1992**, *44*(12), 2289-2295.

[3] Z. Szakonyi, T. A. Martinek, R. Sillanpää, F. Fülöp, Regio-and stereoselective synthesis of the enantiomers of monoterpene-based β-amino acid derivatives, *Tetrahedron: Asymmetry* **2007**, *18*, 2442-2447.
